# Supplementary material for: International meta-analysis of PTSD genome-wide association studies identifies sex- and ancestry-specific genetic risk loci
Source: Nat Commun. 2019 Oct 8;10:4558. doi: 10.1038/s41467-019-12576-w (PMC6783435; doi:10.1038/s41467-019-12576-w)
Supplement: Supplementary file 1 — Supplementary Information [file 41467_2019_12576_MOESM1_ESM.pdf]

International meta-analysis of PTSD genome-wide association studies identifies sex- and ancestry-specific genetic risk loci

Nievergelt *et al*.

Table of contents **Pages**

1. Supplementary Methods	3-24
2. Supplementary Note 1	25-30
3. Supplementary Note 2	31-39
4. Supplementary Figures	40-67
5. Supplementary Tables	68-81
6. Supplementary References	82-91

Supplementary Methods

Participating studies

The following studies were included, listed with the official name of each study followed by a 4-letter abbreviation corresponding to genotyping files and a study number corresponding to **Supplementary Data 1**:

Army Study to Assess Risk and Resilience in Servicemembers (NSS1, NSS2, PPDS; Supplementary Data 1 #14-16)

See reference for details.[1] Potentially traumatic events in childhood and adult civilian trauma were assessed in all participants, as well as military traumatic experience for participants who had been deployed, using a self-administered questionnaire. The extent of traumatic experiences was summarized, separately, in a non-deployment trauma variable and a deployment trauma variable. Both continuous variables are summaries of frequencies of responses to each of the 11 questions regarding non-deployment or deployment trauma. Responses range from Never (0), Once (1), 2-4 Times (2), 5-9 Times (3) or More than 10 Times (4). A combination of a computer-administered version of the Composite International Diagnostic Interview (CIDI) and the Posttraumatic Stress Disorder Checklist (PCL for DSM-IV)[2] were used to assign diagnoses of lifetime Posttraumatic Stress Disorder (PTSD) according to DSM-IV.[3] DNA for GWAS analysis was isolated from blood. The Institutional Review Boards of all participating institutions approved this study.

Ash Wednesday (BRYA; Supplementary Data 1 #10)

See reference for details.[4] Potentially traumatic events were identified using the Recent Life Events questionnaire.[5] The PTSD Checklist (PCL) was used to assess PTSD over the prior 4 weeks by interviews.[2] Respondents were considered to have a diagnosis if DSM-IV criteria were met. The PCL calculates PTSD symptom severity, which ranged from 0 to 68, by clinical interview. For this cohort (187 Cases, 261 Controls), the mean severity was 3.28 and the standard deviation 11.56. DNA for GWAS analysis was isolated from saliva. The Institutional Review Board of Western Sydney Area Health Service approved this study.

Biological Effects of Traumatic Experiences, Treatment and Recovery (BETR; Supplementary Data 1 #48)

See reference for details.[6] In total, 57 PTSD patients, 29 veteran controls (combat controls) and 32 civilian controls (healthy controls) were included. Patients were recruited from one of four outpatient clinics of the Military Mental Healthcare Organization, The Netherlands. Patients were included after a psychologist or psychiatrist diagnosed PTSD. PTSD diagnosis was confirmed using the Clinician Administered PTSD scale (CAPS ≥45[7]). The Structural Clinical interview for DSM-IV (SCID-I[8]) was applied to diagnose comorbid disorders. Control participants were

recruited via advertisements, and the interviews (SCID and CAPS) were also applied to investigate PTSD symptoms and psychiatric disorders. Inclusion criteria for controls were no current psychiatric or neurological disorder, and no presence of current PTSD symptoms (CAPS ≤15). After receiving a complete written and verbal description of the study all participants gave written informed consent. The Medical Ethical Committee of the UMC Utrecht approved the study, and the study was performed in accordance with the Declaration of Helsinki.

Potentially traumatic events were identified using the Life Events Checklist for DSM-IV (LEC-IV).[9] The CAPS for DSM-IV was used to assess PTSD over the prior month by trained researchers.[7] The CAPS calculates PTSD symptom severity, which ranged from 0 to 107, by calculating the total CAPS score. For this cohort (57 Cases, 61 Controls), the mean severity was 50.05 and the standard deviation 32.86. Respondents were considered to have a lifetime diagnosis if DSM-IV criteria were met. Respondents were considered to have a current diagnosis if DSM-IV were met in the previous month. The Institutional Review Board of Utrecht University Medical Center approved this study.

Bounce Back Now (BOBA; Supplementary Data 1 #18)

See reference for details.[10] Potentially traumatic events were identified using National Survey (NSA) on Adolescents PTSD module which was administered by trained interviewers using computer assisted telephone interview technology. The NSA PTSD Module assessed for exposure to five types of potentially traumatic events, in addition to five specific questions about the impact of the tornado (e.g., did the tornado cause damage to your house or property?).[10,11] The NSA PTSD Module that was administered was used to assess PTSD since the tornado, as well as during any two week period in their lifetimes, and lifetime PTSD was used in the present analyses.[11] Respondents were considered to have a lifetime diagnosis if DSM-IV PTSD criteria (i.e., at least one re-experiencing symptom, three avoidance symptoms, and two or more arousal symptoms) were met for at least a two week period. For this cohort, 127 individuals with lifetime PTSD were matched by sex and race/ethnicity with 127 controls who did not meet criteria for lifetime PTSD. DNA for GWAS analysis was isolated from saliva collected via Oragene kits. The Institutional Review Board of the Medical University of South Carolina approved this study.

Childhood Trauma Study (QIMR; Supplementary Data 1 #30)

See references for details.[12,13] Potentially traumatic events were identified using a semi-structured psychiatric diagnostic telephone assessment that included questions on childhood maltreatment.[14] Lifetime DSM-IV PTSD (binary measure) was assessed using a modified version of the measure from the National Comorbidity Survey by telephone interviewers trained by an experienced clinical psychologist.[15] For respondents who had experienced more than one potentially traumatic event, assessment of lifetime PTSD focused on the event identified by each respondent as most disturbing. DNA for GWAS analysis was isolated from blood. The Queensland

Institute of Medical Research Ethics Committee and the Washington University School of Medicine Human Research Protection Office approved this study.

Child Trauma and Neural Systems Underlying Emotion Regulation (KMCT; Supplementary Data 1 #19)

Potentially traumatic events were identified using The UCLA PTSD Reaction Index,[16] the Childhood Experiences of Care and Abuse Interview,[17] and the Childhood Trauma Questionnaire.[18] The Clinician Administered PTSD Scale for Children[19] was used to assess both lifetime and current PTSD by trained clinical interviewers.[17] Children and a parent or guardian completed the interview, and an or rule was used to assign diagnoses. Respondents were considered to have a diagnosis if DSM-5 criteria were met. Respondents were considered to have a current diagnosis if DSM-5 criteria were met. The UCLA PTSD Reaction Index[16] calculates PTSD symptom severity. Children and a parent or guardian each completed this measure, and we used the highest score from either the child or parent, which ranged from 0 to 67 in our sample. For this cohort (133 Cases, 122 Controls), the mean severity was 17.57 and the standard deviation 17.94. DNA for GWAS analysis was isolated from saliva. The Institutional Review Board of the University of Washington approved this study.

CHOICE (FEEN; Supplementary Data 1 #37)

See reference for details.[20-29] Potentially traumatic events were identified using the standard trauma interview.[30] The PTSD Symptom Scale – Interview (PSS-I) was used to assess PTSD over the prior two weeks for the trauma of interest by postdoctoral or graduate level assessors trained to reliability[8]. The Structured Clinical Interview (SCID-IV) was used to assess lifetime PTSD (not current) for a trauma not the focus of treatment by postdoctoral or graduate level assessors trained to reliability.[31] Respondents were considered to have a current diagnosis if on the PSS-I they met symptom-level DSM-IV diagnostic criteria. The PSS-I also provides PTSD symptom severity, with a range from 0 to 51. For this cohort (104 Cases), the mean severity was 32.63 and the standard deviation 4.87. DNA for GWAS analysis was isolated from blood. The Institutional Review Board of University Hospitals approved this study.

Cohen Veterans Center Study (COM1; Supplementary Data 1 #50)

See reference for details.[32-34] This is a multi-site study that ran through NYUMC and Stanford University and Palo Alto VAMC. The VA Palo Alto Health Care System has an affiliation with Stanford University School of Medicine. Dr. Charles Marmar is the overall PI for this study.

Potentially traumatic events were identified using clinical interview that was administered by a licensed psychologist.[35] The CAPS was administered by clinicians to assess PTSD for two time periods: the preceding 30 days and a one-month period

in the past when symptoms were the worst, by respondent's subjective account.[7] The CAPS *calculates PTSD symptom severity by summing the scores for all items, which ranged from 0 to 80 in the full range* and ranged from 0 to 56 in this dataset (in the Cohen Veterans Center study). For this cohort (232 Cases, 802 Controls), the mean severity was 12.14 and the standard deviation 11.85. Respondents were considered to have a current diagnosis if DSM-5 criteria were met in the preceding 30 days. Respondents were considered to have a lifetime diagnosis if DSM-5 criteria were met in the month when symptoms were the worst. The Institutional Review Board of NYUMC and Stanford University approved this study.

Cortical Excitability: Biomarker and Endophenotype in Combat Related PTSD (WANG; Supplementary Data 1 #59)

Potentially traumatic events were identified using CAPS Life Event Checklist. The CAPS was used to assess PTSD over the prior 4 weeks, by research coordinators/interviewers.[7] Respondents were considered to have a diagnosis if CAPS>45. Respondents were considered to have a current diagnosis if CAPS>45. The CAPS calculates PTSD symptom severity, which ranged from 0 to 123 out of a possible 136, by interview.[7] For this cohort (208 Cases, 87 Controls), the mean severity of cases was 76.22 and the standard deviation was 16.85. DNA for GWAS analysis was isolated from whole blood. The Institutional Review Board of Medical University of South Carolina approved this study.

Danish military study (DAMI; Supplementary Data 1 #28)

Potentially traumatic events were identified with 11 single items listing potentially traumatic events occurring during deployment. A scale developed for the Danish military, the PRIM-PTSD, was used to assess PTSD-symptoms over the previous 3 months[36]. The PRIM-PTSD calculates PTSD symptom severity, with a possible range of 12-48. For this cohort (462 Cases, 2019 Controls after quality control), the mean severity was 17.97 and the standard deviation 5.92. PTSD cases were defined as having a PRIM-PTSD score at or above 25, equaling a score of 44 on the PTSD Checklist. DNA for GWAS analysis was isolated from neonatal blood spots. The Regional Committee on Health Research Ethics, Region Zealand, approved this study.

Danish iPSYCH PTSD samples (DAIP; Supplementary Data 1 #29)

See reference for details.[37-39] The Danish iPSYCH PTSD samples were identified for analysis using infrastructure provided by the iPSYCH project.[40] The iPSYCH project is a case cohort study, drawing individuals born in Denmark between 1981 and 2005, and obtaining all cases diagnosed with six disorders plus 30,000 random individuals from the same population cohort as controls. PTSD was not one of the original six diagnoses within iPSYCH, but cases were identified in linked records (i.e. PTSD diagnosis comorbid with one of the six ascertained disorders or

PTSD diagnosis in one of the 30,000 iPSYCH "controls"). PTSD was assessed via clinician diagnosis according to ICD-10 (F43.1), as obtained from either of two registers in Denmark: the Danish Psychiatric Central Research Register and/or the Danish National Patient Register. Diagnoses in the registers are for current disorders (i.e. not lifetime). PTSD severity was not available. DNA for GWAS analysis was isolated from bloodspots from the Danish Neonatal Screening Biobank hosted by the Statens Serum Institut, as described previously.[38,39] The study was approved by the Regional Danish Ethics Committee and the Danish Data Protection Agency.

DCS Rothbaum Study (DCSR; Supplementary Data 1 #38)

See reference for details.[41,42] The authors examined the effectiveness of virtual reality exposure augmented with D-cycloserine or alprazolam, compared with placebo, in reducing PTSD due to military trauma.

After an introductory session, five sessions of virtual reality exposure were augmented with D-cycloserine (50 mg) or alprazolam (0.25 mg) in a double-blind, placebo-controlled randomized clinical trial for 156 Iraq and Afghanistan war veterans with PTSD.[43]

PTSD symptoms significantly improved from pre- to posttreatment across all conditions and were maintained at 3, 6, and 12 months. There were no overall differences in symptoms between D-cycloserine and placebo at any time. Alprazolam and placebo differed significantly on the Clinician-Administered PTSD Scale (CAPS) score at posttreatment and PTSD diagnosis at 3 months posttreatment; the alprazolam group showed a higher rate of PTSD (82.8%) than the placebo group (47.8%).[7] Between-session extinction learning was a treatment-specific enhancer of outcome for the D-cycloserine group only. At posttreatment, the D-cycloserine group had the lowest cortisol reactivity and smallest startle response during virtual reality scenes.

A six-session virtual reality treatment was associated with reduction in PTSD diagnoses and symptoms in Iraq and Afghanistan veterans, although there was no control condition for the virtual reality exposure. There was no advantage of D-cycloserine for PTSD symptoms in primary analyses. In secondary analyses, alprazolam impaired recovery and D-cycloserine enhanced virtual reality outcome in patients who demonstrated within-session learning. D-cycloserine augmentation reduced cortisol and startle reactivity more than did alprazolam or placebo, findings that are consistent with those in the animal literature.

Defining Essential Features of Neural Damage (DEFE; Supplementary Data 1 #12)

See reference for details.[44,45] Potentially traumatic events were identified using the Clinician-Administered PTSD Scale for DSM-IV (CAPS-IV), the Combat Exposure Scale (CES), and items from the Deployment Risk and Resilience Inventory (DRRI).[7,35,46] The CAPS-IV was also used to assess PTSD currently (over the prior 2 weeks) and over the lifetime by trained interviewers.[7] The CAPS-IV calculates PTSD

symptom severity, which ranged from 0 to 120. For this cohort (88 Cases, 62 Controls), the mean severity was 31.43 and the standard deviation 25.01. Respondents were considered to have a lifetime diagnosis if they met DSM-IV criteria for PTSD (measured using CAPS-IV) either current or past. Respondents were considered to have a current diagnosis if they met DSM-IV criteria for PTSD (measured using CAPS-IV) at the time of the assessment. The Institutional Review Board of the Minneapolis VA Health Care System approved this study.

Detroit Neighborhood Health Study (DNHS, ADNH; Supplementary Data 1 #4 and #45)

See reference for details.[47] Potentially traumatic events (PTEs) were identified using a list of 19 PTEs.[48] The PTSD Checklist-Civilian (PCL-C) was used to assess PTSD over the lifetime by self-report during structured telephone interviews by referencing two traumatic events; one the respondent regarded as the worst and a second randomly selected event from the list of remaining PTEs (if the respondent experienced more than one traumatic event).[49] Respondents were considered to have a diagnosis if all six DSM-IV criteria were met for either the worst or random event. Additional questions assessed the timing, duration, severity or illness, and disability resulting from symptoms. PTSD symptom severity, which ranged from 17 to 85, was assessed by summing the respondents' ratings of the 17 post-traumatic symptoms on a scale indicating the degree to which the respondent was bothered by a particular symptom as a result of the worst trauma, ranging from 1 (not at all) to 5 (extremely). All DNHS participants, include N = 2081, of which cases N = 408 and controls N=1532. DNA for GWAS analysis was isolated from peripheral blood or saliva. The Institutional Review Board at the University of Michigan and University of North Carolina Chapel Hill approved this study.

Drakenstein Child Health Study - South African sample (SAFR; Supplementary Data 1 #3)

See reference for details.[50] [51] [52] The modified PTSD Symptom Scale (PSS) was used to assess PTSD.[31] Specifically, the re-experiencing symptom cluster was considered met if the sum of reported symptoms totaled greater than or equal to 1; the avoidance/emotional numbing reported symptoms were greater than or equal to 3; and increased arousal cluster reported symptoms were greater than or equal to 2. Participants who scored above threshold in each of the clusters and had symptom duration for at least 1 month were classified as PTSD cases. The Faculty of Health Sciences human research ethics committee of the University of Cape Town (UCT) approved this study.

EA CRASH (EACR; Supplementary Data 1 #42)

See reference for details.[53] European American individuals were enrolled in the Emergency Department within 24 hours following motor vehicle collision (MVC) trauma/stress.[54] The Impact of Events Scale-Revised (IES-R) was used to assess

PTS symptom severity over the past week by research assistants 1 year following MVC.[54] Respondents were considered to have a diagnosis if they scored 33 or higher on the IES-R questionnaire. The IES-R inventory calculates PTS symptom severity, which ranged from 0 to 88, by scoring a participant's answers to 22 questions on a scale of 0-4 about symptoms of avoidance, intrusions, and hyperarousal. For this cohort (88 Cases, 276 Controls), the mean PTS symptom severity was 19.2 and the standard deviation was 18.4, measured 1-year after the MVC. DNA for GWAS analysis was isolated from blood collected in DNA PAXgene tubes. The Institutional Review Board of The University of North Carolina at Chapel Hill approved this study.

Family Study of Cocaine Dependence and Collaborative Genetic Study of Nicotine Dependence (FSCD, COGA, COGB; Supplementary Data 1 #7-9)

See references for details.[55,56] A module from the Diagnostic Interview Schedule for DSM-IV (DIS-IV),[57] a structured assessment that evaluated the presence or absence of psychiatric disorders according to the DSM-IV[58] criteria was used to evaluate PTSD in a sample of 471 cases and 3,568 controls. A history of fifteen specific traumatic events were queried including rape or sexual assault, assaultive violence (e.g., shot, stabbed), witnessing trauma to others, and non-violent trauma (e.g., serious accident, sudden death of a loved one). The traumatic events were assessed using closed-ended questions (e.g., Have you ever been raped or sexually assaulted?) with nominal response options (i.e., Yes or No). Participants were asked to select the most distressing event and were subsequently evaluated for symptoms of PTSD. A diagnosis of PTSD was dependent on Criterion A, which required intense fear, helplessness, or horror in association with the most distressing event. Interview data were checked for consistency by a senior editor and entered into a computerized data file. Lifetime psychiatric diagnoses were made by a computer algorithm that analyzed responses to the interview using DSM-IV criteria. The Washington University School of Medicine IRB approved the studies.

Fort Campbell study (FTCB; Supplementary Data 1 #52)

Fort Campbell is a United States Army installation located astride the Kentucky-Tennessee border between Hopkinsville, Kentucky, and Clarksville, Tennessee. Fort Campbell is home to the 101st Airborne Division and the 160th Special Operations Aviation Regiment. One thousand, seven hundred and ninety-three (N=1793) active duty members of the Army's 101st Airborne Division who deployed to Afghanistan participated in the study. Each participant was evaluated one-three times at the Fort Campbell U.S. Army military installation. The first evaluation took place prior to deployment in January-February, 2014, the second evaluation took place 3 days upon return from deployment and the third evaluation occurred 90-180 days upon return from deployment.

Potentially traumatic events were identified using self-report questionnaire that included PCL 5.[59] The PTSD Checklist for DSM 5 (PCL-5) was used to assess PTSD during each phase of the study (pre-deployment, 3 days post deployment and 90-180

days post deployment).[59] The PCL-5 score calculates PTSD symptom severity by summing the scores for all items, which ranged from 0 to 80 in the full range and ranged from 0 to 75 in this dataset. For this cohort (114 Cases, 1624 Controls), the mean severity was 6.81 and the standard deviation 11.24. Respondents were considered to have a current diagnosis if PCL5 score is at least 33. The Institutional Review Board of NYUMC and HARPO (DoD IRB) approved this study.

Genetic and Environmental Predictors of Combat-Related PTSD (STRO; Supplementary Data 1 #35)

See reference for details.[60] Subjects in this study were included from a larger STRONG STAR pre-/post-deployment study of deploying soldiers from Fort Hood in Killeen, Texas. The data included in this analysis are from the pre-deployment assessment. Potentially traumatic events were identified using the Life Events Checklist (LEC).[9] The PTSD Checklist–Military Version (PCL-M) was used to assess PTSD over the prior month by self-repor.[2] Subjects were considered to have a current diagnosis if the total score on the PCL was ≥ 50 and classified as a control if their PCL total score was < 50. In addition, both cases and controls were required to report having directly experienced or witnessing a traumatic event on the LEC. Based on these criteria, N=607 subjects were classified as having current PTSD and N=3,390 were classified as controls. DNA for GWAS analysis was isolated from blood. The Institutional Review Board of University of Texas Health Science Center at San Antonio approved this study.

Genetic Risk for PTSD (YEHU; Supplementary Data 1 #56)

See reference for details.[61,62] Potentially traumatic events were identified using the CAPS, SCID, MINI, and the Life Events Checklist.[7,9,63,64] The CAPS, SCID, and MINI were used to assess PTSD during the past month or over the prior lifetime by a PhD level clinical psychologist.[7,63,64] Respondents were considered to have a diagnosis if at least one Criterion B symptom, at least three Criterion C symptoms, at least two Criterion D symptoms, and Criterion A, E, and F (CAPS), J1, J2, and J3 are coded “yes”, at least three or more J4 questions are coded “yes”, at least two J5 answers are coded “yes”, and J6 is coded “yes” (MINI), and/or subject experienced a traumatic event and adverse consequences were experienced, both A criteria were coded 3, at least one B criteria was coded 3, at least three C criteria were coded 3, and at least two D criteria were coded 3 (SCID). Respondents were considered to have a current diagnosis if criteria for PTSD diagnosis were met within the past month. The CAPS calculates PTSD symptom severity, which ranged from 0 to 136. For this cohort (123 cases, 43 controls), the mean severity was 80.19 and the standard deviation 2.23. DNA for GWAS analysis was isolated from whole blood. The Institutional Review Board of James J. Peters VA Medical Center and Icahn School of Medicine at Mount Sinai approved this study.

Genetics of Posttraumatic Stress Disorder/Substance Use Disorder Comorbidity (KSUD; Supplementary Data 1 #17)

See reference for details.[65] Potentially traumatic events were identified using the Life Event Checklist for DSM-5 (LEC-5).[66] The self-report PTSD Checklist for DSM-5 (PCL-5)[59] was used to assess PTSD symptoms over the prior month. The PCL-5 contains 20 items which summed provide a measure of PTSD symptom severity (scores range from 0 to 80). For this cohort (137 Cases, 106 Controls), the mean severity was 40.57 and the standard deviation 20.96. Respondents were considered to have a current diagnosis if total symptom severity was at or above 38. The Institutional Review Board of Kent State University approved this study.

GMRF-QUT (GMFR; Supplementary Data 1 #55)

See reference for details.[67] Potentially traumatic events were identified using Criterion A event. The Clinician Administered PTSD Scale for DSM 5 (CAPS-5) was used to assess PTSD over the prior 2 weeks and lifetime by clinical psychologists.[68] Respondents were considered to have a current diagnosis if CAPS-5 criteria were met. The CAPS-5 calculates PTSD symptom severity, which ranged from 0 to 56. For this cohort (100 Cases, 124 Controls), the mean severity was 9.63 and the standard deviation 10.05. DNA for GWAS analysis was isolated from peripheral blood. Ethics approval for the project was obtained from the Department of Veterans' Affairs, Greenslopes Private Hospital, and Queensland University of Technology Human Research Ethics Committees. This study was carried out in accordance with The Code of Ethics of the World Medical Association (Declaration of Helsinki).

Genetics Research and the Childbearing Year (GRAC; Supplementary Data 1 #54)

See reference for details.[69] A total of 29 potentially traumatic events were identified using the Life Stressor Checklist[70]. PTSD symptoms were assessed using the National Women's Study PTSD Module (NWS-PTSD), a widely used scale designed for use by lay interviewers, that consists of 20 items that assess DSM-III-R PTSD Criteria B, C, and D symptoms. The NWS-PTSD was performed by trained lay interviewers using computer-aided telephone interviewing and epidemiological methods (forced choice yes or no).[71,72] Respondents were considered to have a diagnosis if lifetime DSM-IV PTSD criteria were met. Respondents were considered to have a current diagnosis if past month DSM-IV criteria were met. The NWS-PTSD assesses the number of PTSD symptoms endorsed, which ranged from 0 to 17. For this cohort (140 Cases, 138 Controls), the mean PTSD symptom count (out of 17 possible symptoms) was 6.4 and the standard deviation 5.5. DNA for GWAS analysis was isolated from saliva (Oragene tube). The Institutional Review Board of the University of Michigan approved this study.

Grady Trauma Project (EGHS, GTPC; Supplementary Data 1 #44 and #47)

See reference for details.[73] The modified PTSD Symptom Scale (PSS), a psychometrically valid 17-item self-report scale assessing PTSD symptomatology over the prior 2 weeks, was used to assess PTSD.[74] Consistent with prior literature, the PSS frequency items (0 indicates not at all to 3 indicates ≥5 times a week) to obtain a continuous measure of PTSD symptom severity ranging from 0 to 51. For this sample, the PSS frequency items had standardized α=.90 (mean [SD], 13.81 [11.96]). No clearly established PSS cutoff score for PTSD diagnosis has been established; however, DSM-IV criteria for PTSD can be applied to PSS frequency items to create a proxy variable for PTSD diagnostic status. The Institutional Review Boards of Emory University School of Medicine and Grady Memorial Hospital approved this study.

Growing Up Today Study (GUTS; Supplementary Data 1 #21)

See reference for details.[75] Potentially traumatic events were identified using the Brief Trauma Questionnaire,[76] plus questions on stalking and intimate partner violence (specific events queried included: witness an attack, get attacked, disaster, serious accident, attack on family member, stalked, family member killed in violence, served in war zone/saw war casualties, serious injury to self, physical intimate partner violence, sexual intimate partner violence). Breslau's Short Screening Scale for DSM-IV PTSD was used to assess PTSD over the lifetime by self-report of symptoms.[77] Respondents were considered to have a lifetime diagnosis if they reported experiencing 4 or more symptoms. Current diagnosis was not assessed. Breslau's Short Screening Scale for DSM-IV PTSD calculates PTSD symptom severity, which ranged from 0 to 7, by counting the number of symptoms. For this cohort (312 Cases, 312 Controls), the severity was mean=2.63, SD=2.31 (cases: mean=4.93, SD=0.94; controls: mean=0.34, SD=0.47). DNA for GWAS analysis was isolated from saliva. The Institutional Review Board of Brigham and Women's Hospital approved this study.

Injury and Traumatic Stress Consortium (INTR; Supplementary Data 1 #27)

Subjects were participants in studies of the INTRuST Consortium, some of which are cited.[78-80] Potentially traumatic events were identified using the Life Events Checklist for DSM-IV (LEC)[2] and/or the Deployment Risk and Resilience inventory (DRRI).[81] The PTSD Checklist for DSM-IV (PCL)[2] was used to indicate a likely diagnosis of PTSD (or healthy control status); in some cases, this diagnosis was corroborated by the Clinician-Administered PTSD Scale (CAPS).[7,82] The PCL was used as an indicator of PTSD symptom severity, which ranged from 17-85. DNA for GWAS analysis was isolated from whole blood. The Institutional Review Boards of UCSD (the Coordinating Center) and all the participating institutions approved this research.

IVS (BRYA; Supplementary Data 1 #10)

See reference for details.[83] Potentially traumatic events were identified using the Recent Life Events questionnaire.[5] The Clinician Administered PTSD Scale (CAPS) was used to assess PTSD over the prior 4 weeks by psychologist interviewers.[7] Respondents were considered to have a current diagnosis if DSM-IV were met. The CAPS calculates PTSD symptom severity, which ranged from 0 to 136, by clinical assessment. For this cohort (90 Cases, 312 Controls), the mean severity was 25.10 and the standard deviation 23.98. DNA for GWAS analysis was isolated from saliva. The Institutional Review Board of Western Sydney Area Health Service approved this study.

Marine Resiliency Study (MRSC, BAKE; Supplementary Data 1 #1 and #57)

See reference for details.[84,85] Participants were recruited from two studies including military personnel: (1) the Marine Resiliency Study, a prospective PTSD study with longitudinal follow-up (pre- and post-exposure to combat stress) of U.S. Marines bound for deployment to Iraq or Afghanistan, and (2) a cross-sectional study involving a cohort of combat-exposed active duty or previously deployed service members (CAVC), including PTSD cases and controls with comparable psychosocial and clinical phenotypes. PTSD was diagnosed up to 3 times, once before deployment and 3 and/or 6 month post deployment. Post-traumatic stress (PTS) symptoms were assessed using a structured diagnostic interview, the Clinician Administered PTSD Scale (CAPS), and PTSD diagnosis followed the DSM-IV criteria.[7] All participants included in this study met the *DSM-IV* criteria A1 event. For participants assessed at multiple timepoints, the timepoint with the highest CAPS score was used. Genomic DNA was prepared from blood leukocytes and genotyping was carried out by Illumina (http://www.illumina.com/) using the HumanOm-niExpressExome (HOEE) array with 951,117 loci and by RUCDR (http://www.rucdr.org) using the HOEE array with 967,537 loci. The study was approved by the University of California San Diego Institutional Review Board, and all participants pro-vided written informed consent to participate.

McLean Trauma Sample (TEIC; Supplementary Data 1 #39)

The McLean Trauma Sample consists of three separate studies lead by Drs. Milissa Kaufman and Martin Teicher. See references for details.[86-88] The first study was conducted at McLean Hospital’s Developmental Biopsychiatry Research Program (PI: Martin Teicher, MD, PhD) entitled “Sensitive Periods, Brain Development and Depression Study”. The group aimed to test the hypothesis that there are discrete sensitive periods when exposure to abuse or loss is maximally associated with risk for developing psychiatric disorders, specifically major depression and that risk for developing depression coincided with exposure to abuse during sensitive periods of hippocampal and prefrontal cortex vulnerability. These hypotheses were tested in a sample of 517 individuals (20-25 years of age) recruited from the community. Degree and timing of developmental exposure to abuse and loss across each childhood stage was quantified retrospectively using the

Maltreatment and Abuse Chronology of Exposure (MACE) scale,[89] as well as Traumatic Antecedent Interview,[90] Childhood trauma Questionnaire[91] and Adverse Childhood Experiences scale.[92] Lifetime and current psychopathology including PTSD was assessed by trained psychologists, psychiatrists and clinical nurse specialists, using the Structured Clinical Interview for DSM-IV-TR.[93] Respondents were considered to have a current diagnosis if DSM-IV-TR criteria were met in the preceding 30 days.

The second study was also conducted at McLean Hospital's Developmental Biopsychiatry Research Program (PI: Martin Teicher). The key aims of the project were to test in a prospective study whether neurobiological correlates such as T2-relaxation time in dorsolateral prefrontal and anterior cingulate cortex and a large cerebellar lingual size can predict degree of drug and alcohol use in individuals with histories of childhood abuse and neglect, with an emphasis on sensitive periods of maximal exposure. These hypotheses were tested in a sample of 157 individuals (18-19 years of age) recruited from the community. Structured Clinical Interviews for DSM-IV (SCID-IV) Axis I and II psychiatric disorders were used for diagnoses.[93] Mental health professionals (psychiatrists, Ph.D. psychologists, clinical nurse specialists) performed all the interviews and psychological assessments. In addition to the Maltreatment and Abuse Chronology of Exposure (MACE) scale,[89] the 100-item semi-structured Traumatic Antecedents Interview[90] was also used to assess maltreatment history, as well as the Childhood Trauma Questionnaire[91] and the Adverse Childhood Experience score.[92] PTSD was diagnosed using the SCID-IV-TR and the CAPS.[7]

The third study was conducted at McLean's Dissociative Disorders and Trauma Research Program (PI: Milissa Kaufman, MD, PhD) entitled "Evaluating the Neurobiological Basis of Traumatic Dissociation in a Cross-Diagnostic Sample of Women with Histories of Childhood Abuse and Neglect". Patients were recruited from inpatient and partial/residential treatment programs at McLean Hospital as part of a larger study on trauma-related dissociation comprised of diagnostic interviews, self-reports, neuropsychological testing, and neuroimaging protocols. All individuals endorsed a history of childhood trauma exposure, as assessed by the Traumatic Events Interview and Childhood Trauma Questionnaire. All participants also met criteria for DSM-5 PTSD as assessed by the Clinician-Administered PTSD Scale for DSM-5.

The Institutional review Board of McLean Hospital approved all studies. All saliva samples were collected using Oragene DNA collection kits (DNA Genotek) according to the instructions of the manufacturer.

Mid-Atlantic Mental Illness Research Education and Clinical Center the study of Post-Deployment Mental Health Study (MIRE; Supplementary Data 1 #26)

See reference for details of the study of Post-Deployment Mental Health (1,308 cases, 1,914 controls).[94,95] PTSD was diagnosed using the Structured Clinical Interview for DSM-IV Disorders (SCID) administered by trained interviewers.[8] In

accordance with the DSM-IV, PTSD consisted of three symptom clusters. These included re-experiencing symptoms (B symptoms), avoidance and numbing symptoms (C symptoms) and hyperarousal symptoms (D symptoms). Total PTSD symptoms and symptom clusters (B, C, or D) were measured using the Davidson Trauma Scale for all veterans, including individuals with current PTSD diagnosis and controls. The research was reviewed and approved by the Institutional Review Boards at the Salisbury, NC VA, Hampton, VA VA, Richmond, VAVA, Durham, NC VA and Duke University Medical Centers.

National Centre for Mental Health (NCMH; Supplementary Data 1 #41)

See www.ncmh.info for details. Potentially traumatic events were identified by participant self-report. The Trauma Screening Questionnaire (TSQ) was used to assess PTSD over the prior 2 weeks by self-report.[96] Respondents were considered to have a current diagnosis if a score of 6 or more was obtained. The TSQ calculates PTSD symptom severity, which ranged from 0 to 10, by self-report. For this cohort (631 Cases, 653 Controls), the mean severity was 5.32 and the standard deviation 3.47. DNA for GWAS analysis was isolated from blood or saliva. The study was given a favorable ethical opinion by Wales Research Ethics Committee (REC) 2.

National Health and Resilience in Veterans Study (NHRV; Supplementary Data 1 #13)

See reference for details.[97] Potentially traumatic events were identified using the Trauma History Screen.[98] The PTSD Checklist-Specific (PCL-S) was used to assess both lifetime and past-month PTSD symptoms related to respondent's 'worst' traumatic event over their lifetimes by survey.[2] Respondents were considered to have screen positive for PTSD if their PCL-S score was ≥50. The PCL-S calculates PTSD symptom severity, which ranged from 17 to 85, by self-report. For this cohort (95 Cases, 1490 Controls), the mean PCL-S severity score was 26.9 and the standard deviation 11.3. DNA for GWAS analysis was isolated from saliva. The Human Subjects Subcommittee of VA Connecticut Healthcare System approved this study.

NIU Trauma Study (NIUT; Supplementary Data 1 #40)

See reference for details.[99,100] Potentially traumatic events were identified using the Traumatic Life Events Questionnaire and 12 questions regarding level of exposure to the campus shooting.[101] The Distressing Events Questionnaire was used to assess PTSD immediately following the mass shooting (average 3.2 weeks) by self-report.[102] Respondents were considered to have a diagnosis if their DEQ score was ≥ 18. The DEQ calculates PTSD symptom severity, which ranged from 0 to 66, by self-report. For this cohort (280 Cases, 411 Controls), the mean severity was 16.49 and the standard deviation 12.35. DNA for GWAS analysis was obtained from 204 of the PTSD cases and was isolated from saliva. The Institutional Review Board of Northern Illinois University approved this study.

Nurses Health Study II (NHS2; Supplementary Data 1 #5)

See reference for details.[103] Participants identified stressful events they had experienced from a list of 25 events used in diagnostic interviews, [104, 28,105-107] and PTSD was assessed in relation to participants' self-selected worst stressful event. Participants were cued to think of the period following the event during which symptoms were most frequent and intense. They were asked whether they had ever been bothered by each of 17 symptoms and rated each symptom on a Likert-style scale (1: "not at all" to "5: extremely").[108] Additional questions assessed the other three DSM-IV criteria: intense fear, horror, or helplessness in response to the event (Criterion A2), symptom duration of at least one month (Criterion E), and clinically significant impairment in functioning due to symptoms (Criterion F)[104]. Based on the diagnostic interview, we created two lifetime PTSD phenotypes as follows.

To meet criteria for lifetime PTSD diagnosis, respondents must have endorsed experiencing one or more of the 5 re-experiencing symptoms, 3 or more of the 7 avoidance/numbing symptoms, 2 or more of the 5 arousal symptoms, and criteria A2, E and F as defined above. In addition to the diagnostic phenotype, we analyzed lifetime PTSD symptom severity which was defined as the sum of the symptom ratings across the 17 questions.

The reliability of the PTSD diagnosis was assessed using a blind review of audiotapes from 50 interviews and the Cohen's kappa statistic was 1.0 (perfect reliability).[109] We assessed the validity of our identification of PTSD in a separate cohort, the Detroit Neighborhood Health Study, via clinical interviews among a random subsample of 51 participants and found excellent concordance.[47] The Partners Human Research Committee approved this study.

Nurses Health Study II (NHSY; Supplementary Data 1 #22)

See reference for details.[110] The Nurses' Health Study II is an ongoing cohort of 116,430 female nurses initially enrolled in 1989 and followed with biennial questionnaires. The present study included follow-up through 2013. This study included women who returned a supplementary 2008 questionnaire on trauma exposure and PTSD symptoms (*N*=54,763). This questionnaire was sent to a subsample of participants (*N*=60,804, response rate=90.1%). To retain participation in the ongoing longitudinal cohort, only women who have already returned their biennial questionnaire are sent supplementary questionnaires. Women missing data on trauma or PTSD symptoms (*N*=3,930) were excluded. This study was approved by the Institutional Review Board of Brigham and Women's Hospital. Return of the questionnaire via US mail constitutes implied consent.

Trauma exposure and PTSD symptoms were assessed on a supplementary 2008 questionnaire. The 16-item Brief Trauma Questionnaire queried lifetime exposure to 15 types of traumatic events (e.g., serious car accident, sexual assault) and an additional item queried any traumatic event not covered in the other questions. [76] Respondents were asked to identify which trauma was their worst or most distressing; they were then asked their age at this worst trauma as well as their

age at their first trauma. PTSD symptoms were assessed in relation to their worst trauma with the 7-item Short Screening Scale for DSM-IV PTSD.[77] Four or more symptoms on this scale have been associated with PTSD diagnosis (sensitivity=80%, specificity=97%, positive predictive value=71%, negative predictive value=98%).[77] For cases women with ≥4 PTSD symptoms were selected, for controls women with <4 PTSD symptoms (most had 0) were selected.

Ohio National Guard (ONGA; Supplementary Data 1 #2)

See reference for details.[111] A total of 37 potentially traumatic events were identified using the Clinician-Administered PTSD Scale (CAPS-IV)[7] and the 1996 Detroit Area Survey of Trauma.[48] PTSD symptoms were assessed using a 17-item structured interview scale derived from the PTSD Checklist (PCL) for DSM-IV[2] performed by trained lay telephone interviewers using epidemiological methods (forced choice symptom severity range, 1-5). Reliability of the telephone interview was validated against the criterion standard (in-person CAPS interview by mental health professional) in a clinical subsample (N = 500), demonstrating high specificity (0.92).[112] Respondents were considered to have a diagnosis if lifetime DSM-IV PTSD criteria were met. Respondents were considered to have a current diagnosis if past month DSM-IV criteria were met. The PCL calculates PTSD symptom severity, which ranged from 17 to 85, by sum of scores of items endorsed. For this cohort (125 Cases, 125 Controls), the mean severity was 38.4 and the standard deviation 17.6. DNA for GWAS analysis was isolated from saliva (Oragene tube). The Institutional Review Board of VA Ann Arbor Health System approved this study.

OPT (FEEN; Supplementary Data 1 #37)

Potentially traumatic events were identified using the standard trauma interview.[30] The PSS-I was used to assess PTSD over the prior two weeks for the trauma of interest by postdoctoral and graduate level assessors trained to reliability.[31] The SCID-IV was used to assess lifetime PTSD (not current) for a single trauma not the focus of treatment.[8] Respondents were considered to have a current PTSD diagnosis if they met DSM-IV diagnostic criteria and had a PSS-I score of 25 or greater. The PSS-I also provides PTSD symptom severity, with a range from 0 to 51. For this cohort (118 cases), the mean severity was 30.36 and the standard deviation 6.91. DNA for GWAS analysis was isolated from blood. The Institutional Review Board of University Hospitals approved this study.

Portugal (PORT; Supplementary Data 1 #20)

See reference for details.[113] Potentially traumatic events were identified using CIDI, which included a module on DSM-IV PTSD that inquired about lifetime exposure to each of 27 different traumatic events (criterion A1). Respondents who reported ever experiencing any of the traumatic events were then asked about the number of exposures (NOE) and age at first exposure (AOE) for each.[104] The CIDI

was used to assess PTSD over the prior Lifetime DSM-IV PTSD and other common DSM-IV disorders.[104] DNA isolated from saliva. The Institutional Review Board of the Nova Medical School, Portugal, approved this study.

PRISMO (PRIS; Supplementary Data 1 #25)

See reference for details.[114] Potentially traumatic events were identified using a 19-item checklist.[114] The Dutch Self-Rating Inventory for PTSD (SRIP) was used to assess PTSD one month prior to deployment and up to 2 years after deployment.[115] Respondents were considered to have a current diagnosis if a SRIP total score of ≥ 38 at any measurement was met. The SRIP calculates PTSD symptom severity, which ranged from 22 to 88, by self-report. For this cohort (144 Cases, 815 Controls), the mean severity was 27.30 and the standard deviation 6.38. DNA for GWAS analysis was isolated from whole blood. The Institutional Review Board of Utrecht University Medical Center approved this study.

Pregnancy Outcomes, Maternal and Infant Study Cohort (PROM; Supplementary Data 1 #23)

See reference for details.[116] Potentially traumatic events were experiences of intimate partner violence [identified using Demographic Health Survey Questionnaires and Modules: Domestic Violence Module[117] and the World Health Organization Multi-Country Study on Violence Against Women][118] and history of childhood abuse [identified using the Childhood Physical and Sexual Abuse Questionnaire adopted from the Center for Disease Control and Prevention Adverse Childhood Experiences Study].[119] The Posttraumatic Stress Disorder Checklist – Civilian Version (PCL-C) was used to assess PTSD over the prior month.[120] The PCL-C is a self-report measure with 17 items reflecting DSM-IV symptoms of PTSD and closely follows the Diagnostic and Statistical Manual of Mental Disorders (DSM-IV) criteria. For each item, participants were asked how bothered they were by a symptom over the past month on a 5-point Likert scale ranging from 1: “not at all” to 5: “extremely” in regards to their most significant life event stressor. The total score on the PCL-C ranges from 17 to 85. Recent data from our team support that a PCL-C score of 26 or higher on the Spanish-language version, is associated with an 86% sensitivity and 63% specificity in diagnosing PTSD in a Peruvian population using the Diagnostic and Statistical Manual of Mental Disorders (DSM-IV) criteria.[120]

All study procedures were approved by the Instituto Nacional Materno Perinatal in Lima, Peru, and the Office of Human Research Administration at the Harvard T.H. Chan School of Public Health, Boston, MA.

Readiness and Resilience in National Guard Soldiers (RING; Supplementary Data 1 #33)

See reference for details.[121] Potentially traumatic events were identified using the Clinician-Administered PTSD Scale for DSM-IV (CAPS-IV) and items from the Deployment Risk and Resilience Inventory (DRRI)[7,46] The CAPS-IV and the PTSD Checklist (PCL) were also used to assess PTSD currently (over the prior 2 weeks) and over the lifetime by trained interviewers and through self-report.[7,122] The PCL calculates PTSD symptom severity, which ranged from 17 to 73 currently and 17 to 85 lifetime, by self-report. For this cohort (41 Cases, 162 Controls), the mean current severity was 34.03 and the standard deviation was 13.48. The mean lifetime severity was 33.89 and the standard deviation was 12.98. Respondents were considered to have a lifetime diagnosis if they had a PCL score $\geq$ 50 at the one-year post-deployment time point. Respondents were considered to have a current diagnosis if the same criteria were met at the two-year post-deployment time point. The Institutional Review Board of the Minneapolis VA Health Care System approved this study.

Risbrough/Norman randomized controlled psychotherapy trial (VRIS; Supplementary Data 1 #58)

See reference for details.[123] Potentially traumatic events were identified using the life event checklist. The CAPS was used to assess PTSD over the prior over the past month by interviewers.[68] Respondents were considered to have a diagnosis if DSM-5 diagnostic criteria using the CAPS-5 (a criterion A trauma and 2 or higher on 1 re-experiencing symptom (criterion b), 1 avoidance symptom (criterion c), 2 negative alterations in cognitions or mood (criterion d), and 2 hyperarousal symptoms (criterion e)) were met. The CAPS calculates PTSD symptom severity, which ranged from 0 to 80 by clinician rater of symptoms and severity. For this cohort (73 Cases, 5 sub-clinical), the mean severity was 42 and the standard deviation 9.6. DNA for GWAS analysis was isolated from saliva using Oragene. The Institutional Review Board of The San Diego VA approved this study.

Shared Roots (SHRS; Supplementary Data 1 #46)

See reference for details.[66] Potentially traumatic events were identified using The Life Events Checklist for DSM-5 (LEC-5).[66] The Clinician-Administered PTSD Scale for DSM-5 (CAPS-5)[68] was administered by clinicians to assess PTSD over the prior month. The CAPS-5 and the PTSD Checklist for DSM-5 (PCL-5)[59] calculates PTSD symptom severity, with a score range of 0 to 80, by adding scores ranging from 0 to 4 for all twenty items. For this cohort (164 Cases, 164 Controls), the mean severity on the PCL-5 was 33.0 and the standard deviation 23.9. A lifetime diagnosis of PTSD was not assessed for in this study. Respondents were considered to have a current diagnosis if DSM-5[124] criteria based on the CAPS-5 were met. The Institutional Review Board of Stellenbosch University approved this study.

Southeastern Europe PTSD (SEEP; Supplementary Data 1 #49)

See reference for details.[125] Potentially traumatic events were identified using Life Stressor List and List of traumatic events including frequency and severity of traumatic events.[126] The CAPS was used to assess lifetime PTSD by medical personnel (psychiatrists, psychologists or psychiatric residents).[7] Respondents were considered to have a diagnosis if DSM-IV criteria were met over lifetime. The CAPS calculates PTSD symptom severity, which ranged from 27 to 141. For this cohort (347 Cases, 339 Controls), the mean severity was 74.3 and the standard deviation 20.2. DNA for GWAS analysis was isolated from EDTA blood. The Institutional Review Board of the universities of Sarajevo, Zagreb, Tuzla, Mostar, Prishtina and Würzburg approved this study.

Study of Aftereffects of Trauma: Understanding Response in National Guard (SATU; Supplementary Data 1 #11)

See reference for details.[127] Potentially traumatic events were identified using Clinician-Administered PTSD Scale for DSM-IV (CAPS-IV).[7] The CAPS-IV was also used to assess PTSD currently (over the prior 2 weeks) and over the lifetime by trained interviewers[7]. The CAPS-IV calculates PTSD symptom severity, which ranged from 0 to 105. For this cohort (88 Cases, 62 Controls), the mean severity was 50.33 and the standard deviation 24.55. Respondents were considered to have a lifetime diagnosis if they met DSM-IV criteria for PTSD (measured using CAPS-IV) either current or past. Respondents were considered to have a current diagnosis if they met DSM-IV criteria for PTSD (measured using CAPS-IV) at the time of the assessment. The Institutional Review Board of the Minneapolis VA Health Care System approved this study.

Sydney Neuroimaging (BRY2; Supplementary Data 1 #36)

Potentially traumatic events were identified using clinical interview to identify history of traumatic events. The CAPS was used to assess PTSD over the prior 4 weeks by Masters level clinical interviewers.[7] Respondents were considered to have a diagnosis if DSM-IV criteria were met. The CAPS calculates PTSD symptom severity, which ranged from 0 to 136, by clinical interview. For this cohort (82 Cases, 86 Controls), the mean severity was 39.67 and the standard deviation 31.75. DNA for GWAS analysis was isolated from saliva. The Institutional Review Board of Western Sydney Area Health Service approved this study.

VA Boston-National Center for PTSD Study (NCPT, TRAC; Supplementary Data 1 #31, Supplementary Data 1 #32)

A total of 437 white non-Hispanic cases and 215 trauma-exposed controls is the composite of two datasets. The first from a cohort of white non-Hispanic subjects as described in a previous GWAS[128] that passed ancestry filters as performed using SNPweights[129] according to PGC-PTSD protocols (300 cases and 165 controls; 305 males and 160 females). The majority of this sample consisted of US veterans, but

also included the intimate partners of a subset of the veterans. The second cohort was made up of subjects from the Translational Research Center for TBI and Stress Disorders, a VA RR&D Traumatic Brain Injury Center of Excellence at VA Boston Healthcare System (TRACTS) study of US veterans. From TRACTS, 137 white non-Hispanic cases and 50 controls passed ancestry filters based on SNPweights and were included in the analysis. The TRACTS sample is largely male (170 men and 17 women). The genotyping, quality control, filtering and imputation for these cohorts has been described in detail elsewhere.[128,130] Briefly, genotyping was performed using the Illumina HumanOmni2.5-8 microarrays (Illumina, San Diego, CA). Imputation of non-genotyped SNPs was performed using IMPUTE2[131-134] and 1000 genomes phase 1 reference data.[135] Principal components were generated by the program EIGENSTRAT[136] based on 100,000 SNPs. For both cohorts, participants were administered the Clinician-administered PTSD scale for DSM-IV (CAPS-IV),[7] a 30-item structured diagnostic interview that assesses the frequency and severity of the 17 DSM-IV PTSD symptoms, 5 associated features and functional impairment, and both current and lifetime PTSD symptoms. These studies were performed under the oversight of the appropriate VA health care facilities institutional review boards.

UK Biobank Cohort Description for PGC-PTSD (UKBB; Supplementary Data 1 #60)

The UK Biobank is an epidemiological resource assessing a range of health-related phenotypes in approximately 500,000 British individuals who were recruited between the ages of 40 and 70.[137] Genome-wide genotype data is available on all participants, as well as a broad range of health phenotypes assessed at varying intensity. Data from an online follow-up questionnaire assessing common mental health traits, including questions designed to screen for PTSD, was available on 157,366 individuals.[138]

Phenotype: PTSD phenotypes were derived from the mental health online follow-up of the UK Biobank (Resource 22 on http://biobank.ctsu.ox.ac.uk). Participants were asked six questions derived from the brief civilian version of the PTSD Checklist Screener (PCL-S;[82]) assessing PTSD symptoms in the prior month. Questions comprised three initial questions related to avoidance of activities, disturbing thoughts, and feeling upset, and three further questions related to feeling distant, feeling irritable and having trouble concentrating (UK Biobank fields 20494-20498, 20508). Each item was scored on a five-point scale according to the amount of concern caused by that item in the past month (1="Not at all" to 5="Extremely"). The final item concerning trouble concentrating was drawn from an equivalent item from the Patient Health Questionnaire-9 (PHQ9) depression questionnaire and was scored on a four-point scale according to frequency of difficulties (1="Not at all" and 4="Nearly every day"). All items were summed for each individual to yield a total score ranging 3-29. Cases were defined as all individuals with a PCL-S score ≥ 13, controls as all individuals who responded to all of the initial three questions and had PCL-S score ≤ 12.

Two sensitivity analyses were performed to assess the stability of the UK Biobank PTSD phenotype. Firstly, controls were limited only to those who answered all initial questions with "Not at all" and so had a PCL-S score = 3. Secondly, PTSD cases and controls were limited to those reporting a lifetime trauma exposure. To assess the impact of reported trauma exposure on the PTSD phenotype, a trauma exposure measure was derived from questions in the mental health online follow-up that related to common triggers of post-traumatic stress-disorder.[138] These questions asked if participants had ever: experienced combat; had a life-threatening accident; been diagnosed with a life-threatening illness; been a victim of a physically violent crime; been a victim of sexual assault; or witnessed a sudden violent death. Responses were combined to a single variable capturing any report of traumatic experience versus no report.

Genetic quality control: Genetic data for analyses was obtained from the full release of the UK Biobank data (N=487,410).[139] Individuals were removed if this was recommended by the UK Biobank for unusual levels of missingness or heterozygosity; if call rate < 98% on genotyped SNPs; if they were related to another individual in the dataset (KING r < 0.044, equivalent to removing up to third-degree relatives inclusive); and if the phenotypic and genotypic gender information was discordant (X-chromosome homozygosity (FX) < 0.9 for phenotypic males, FX > 0.5 for phenotypic females). Removal of relatives was performed using a greedy algorithm, which minimise exclusions (for example, by excluding the child in a mother-father-child trio). All analyses were limited to individuals of White Western European ancestry, as defined by 4-means clustering on the first two genetic principal components provided by the UK Biobank.[140] Principal components analysis was also performed on the European-only subset of the data using the software package flashpca2[141]. After quality control, individuals were excluded from analysis if they did not complete the mental health online questionnaire (N=126,522).

Genetic analyses used imputed variants provided by the UK Biobank.[139] Autosomal genotype data from two highly-overlapping custom genotyping arrays (covering ~800,000 markers) underwent centralised quality control to remove genotyping errors before being imputed in a two-stage imputation to the Haplotype Reference Consortium (HRC) and UK10K (for rarer variants not present in the HRC) reference panels [(142;143;139)]. In addition, this central quality control, variants for analysis were limited to common variants (minor allele frequency > 0.01) imputed with higher confidence (IMPUTE INFO metric > 0.4). In addition, only variants that were directly genotyped or that were imputed from the HRC were included.[142]

Genome-wide association analyses: Prior to analysis, PTSD status was residualised on six prinicipal components from the genetic data and factors capturing genotyping batch and recruitment centre, using logistic regression. GWAS were then performed on the resulting deviance residuals using linear regressions on imputed genotype dosages in BGenie v1.2, software written for genetic analyses of UK Biobank.[139]

Vietnam Era Twin Study of Aging (VETS; Supplementary Data 1 #24)

See references for details.[144,145] Potentially traumatic events were identified using the Combat Exposure Index[146] and the Diagnostic Interview Schedule Version III-Revised (DIS-III-R).[DIS-III-R; 147] The Vietnam Era Twin Registry PTSD scale[148] (administered at average age 38) was used to assess PTSD symptoms over the past 6 months and the PCL-civilian version for DSM-IV[149] (administered at average age 62) was used to assess PTSD over the prior month. These two instruments correlate 0.90 when administered at the same time.[150] The 17-item PCL calculated PTSD symptom severity, which ranged from 17 to 84. Each response was rated on a 1-5 scale (from “not at all” to “extremely”). For this cohort (60 Cases, 841 Controls), the mean severity was 26.2 and the standard deviation was10.5. For this analysis, respondents were considered to have a diagnosis of PTSD if they met DSM-III-R criteria based on the DIS-II-R interview. DNA for GWAS analysis was isolated from blood. Genotyping was performed by deCODE Genetics, Reykjavik, Iceland. The Institutional Review Boards of the University of California, Sand Diego, Boston University, and the Puget Sound VA Healthcare System approved this study.

The Women and Children’s Health Study (WACH; Supplementary Data 1 #43)

See reference for details.[151] Potentially traumatic events were identified using the Life Events Checklist (LEC) for DSM-5.[66] The PTSD Checklist for DSM-5 (PCL-5) was used to assess PTSD over the lifetime by interviewers.[152] Respondents were considered to have a diagnosis of PTSD if the PCL-5 score was >=38. Respondents were considered to have a current diagnosis based only upon self-report during the interview. The PCL-5 calculates PTSD symptom severity, which ranged from 0 to 79. For this cohort (151 Cases, 150 Controls), the mean severity was 52.4 (SD: 11.2) for cases and 31.0 (SD: 23.5) for controls DNA for GWAS analysis was isolated from blood. The Institutional Review Board of the Louisiana State University Health Sciences Center-New Orleans approved this study.

Yale-Penn Study (GSDC; Supplementary Data 1 #6)

See reference for details.[153] Sample collection and diagnostic interviews were performed by trained interviewers using the Semi-Structured Assessment for Drug Dependence and Alcoholism (SSADDA; available at https://zork.wustl.edu/nida/study_descriptions/study_1/ssaddav11_2_ns.pdf) to derive diagnoses for lifetime psychiatric and substance use disorders based on DSM-IV criteria. Twelve types of traumatic events were assessed: experienced direct combat in a war; seriously physically attacked or assaulted; physically abused as a child; seriously neglected as a child; raped; sexually molested or assaulted; threatened with a weapon; held captive or kidnapped; witnessed someone being badly injured or killed; involved in a flood, fire, or other natural disaster; involved in a life-threatening accident; suffered a great shock because one of the above events happened to someone close to you; and other. Participants were asked to list up to three traumatic events and describe the trauma in detail. Those reporting traumatic experiences were then interviewed for potential PTSD symptoms. After the data were

scored, PTSD diagnoses were generated based on DSM-IV criteria. The institutional review boards at Yale University School of Medicine, the University of Connecticut Health Center, the University of Pennsylvania School of Medicine, the Medical University of South Carolina, and McLean Hospital approved the study.

Supplementary Note 1

Participating studies and ancestry compositions

The PGC-PTSD Freeze 2 dataset (PGC2) is comprised of a total of 206,655 subjects (32,428 PTSD cases and 174,227 controls) with available genotype and phenotype information (Supplementary Data 1). PGC2 includes subjects from 9 studies already reported in Freeze 1[154], 50 (combined into 47) new studies (together forming Freeze 1.5: *N* = 80,467 subjects, 22,039 cases, 58,428 controls), and the large European ancestry UK Biobank study (UKBB; *N* = 126,188 subjects, 10,389 cases, 115,799 controls). Mean study age of these subjects ranged from 13.35 – 69.1 years (mean 52.4 years), and the male/female ratio ranged from all female (*N* = 7) to all male (*N* = 3) studies (mean 49.7% male).

PGC1.5 included populations from South Africa, Europe, and the Americas, and only 56.5% of subjects with individual-level genotype data were of European ancestry (Supplementary Table 12). After classification of subjects into main ancestral groups (Supplementary Figure 1) and removal of related subjects, a total of 195,701 subjects (94.7%; 29,556 PTSD cases and 166,145 controls) remained to be included in the European (EUA), African (AFA), and American ancestry (AMA) meta-analyses (Supplementary Tables 3, 5, 6). Due to the smaller sample size (N = 5,703 subjects), the AMA meta-analysis was only considered in trans-ancestry analyses and replication of the top hits in the EUA and AFA GWAS.

Polygenic effects vs. population stratification

Meta-analyses were performed for the EUA, AFA, and AMA GWAS in both sexes and separately in men and women. Quantile-quantile plots across all analyses showed low genome-wide inflation of the test statistics (Supplementary Figure 2). To further distinguish true polygenic effects from potential population stratification and cryptic relatedness (e.g. among subjects from studies sharing summary data) within these inflation statistics, the LDSC intercept method was used (EUA analyses only). In the meta-analysis including both sexes, the estimated polygenic effects account for 72% (SE = 0.082) of the observed inflation (Supplementary Figure 2, panel A), with a remaining modest but significant inflation (intercept = 1.022, SE = 0.006, $P = 6.22 \times 10^{-4}$) potentially reflecting cryptic relatedness, population stratification, and/or sample size. In the male-only analysis (Supplementary Figure 2, panel E), the polygenic effects account for 15% of the test statistics, with a remaining modest but significant inflation (intercept =1.026, SE = 0.006, $P = 9 \times 10^{-6}$), and in the female-only analysis polygenic effects account for 100% of the observed genome-wide inflation of the test statistics (Supplementary Figure 2, panel I), with no significant evidence of remaining inflation (intercept = 0.989, SE = 0.006, *P* = 0.08).

Sensitivity analyses for heritability estimates in UK Biobank using alternative subject selection criteria

Because the UKBB contributed a substantial number of subjects to PGC2 (45% of the EUA cases), we performed additional heritability analyses using two additional,

more restrictive case and control definitions (Supplementary Table 1, p2 and p3). Heritability point estimates in these smaller data sets remained similar across all analyses; as expected, h^2_{snp} was most significant in the largest, least restrictive subject pool (p1). Importantly, in the UK biobank, h^2_{snp} estimates were not different between men and women across all subject selection criteria (P >0.4 in all cases). Subsequent GWAS analyses were based on the UKBB phenotype including the largest number of subjects (P1; N = 126,188).

Local ancestry inference (LAI)

To deconvolute local ancestry across the genome of admixed subjects we developed a custom LAI pipeline (see Methods and Supplementary Figure 23 for details). Overall, we found that global ancestry proportions for admixed individuals calculated from local ancestry tracts were highly concordant with ancestry estimates generated by PCA-based SNPweights (see Methods; correlations: r = 0.950 for the European ancestry components, and r = 0.992 for African ancestry, N = 7,206 mixed-ancestry subjects from the entire GTPC).

Local ancestry analyses for the two lead SNPs from the genome-wide significant genomic regions in AFA are presented in the Supplementary Tables 7-8. Only subjects with available genotype data are included in these analyses. An analysis of allele frequencies stratified by copies and type of ancestral haplotypes showed that rs115539978 is present predominantly on the African background (MAF 8-9%), with MAF< 1% on the European and Native American background (Supplementary Table 7). Inclusion of LAI covariates did not influence the effect of rs115539978 on PTSD (i.e., global PC's appropriately controlled for admixture; panel B). Due to differences in allele frequencies, rs115539978 is an ancestry-specific PTSD risk variant. The male-specific hit with lead SNP rs142174523 was common on all ancestral backgrounds (Supplementar Table 8). Association with PTSD was robust to inclusion of LAI covariates, and the minor allele had a similar, protective effect on the African and European background (OR = 0.8).

Biological function of genes and psychiatric relevance

Functional annotation of the 6 GWAS hits and gene-based analyses using FUMA predicted 12 genes to be associated with PTSD in the EUA and AFA GWAS (Table 4).

ZDHHC14 (Zinc finger, DHHC-type containing 14): *ZDHHC14* encodes a palmitoyltransferase that is expressed in the brain[155] and evidence from other DHHC containing palmitoyl acyltransferases support a role for dysregulated palmitoylation contributing to neuropsychiatric disorders[156]. *ZDHHC14* was previously identified as a candidate gene for bipolar disorder by linkage and convergent functional genomics studies[157], but to date GWAS does not support a significant association between *ZDHHC14* and bipolar disorder.

PARK2 (Autosomal recessive juvenile Parkinson disease-2) is a component of the E3 ubiquitin ligase complex and is known to have several functions[158], including the autophagy of dysfunctional mitochondria, which plays a neuroprotective role in

familial Parkinson’s disease.[159] *PARK2* may also act to regulate innate immunity and inflammation[65,160,161] and function as a tumor suppressor [162]. PARK2 shows an extensive alternative splicing pattern[163], however, the role of different isoforms in the brain is not well understood. PARK2 codes for PARKIN, loss of function of the PARKIN protein leads to dopaminergic cell death although the mechanism by which this occurs is not clear. The dopaminergic system has an important role in fear conditioning, which is critical in the development and maintenance of PTSD.[164] Abnormal expression of *PARK2* has been seen in major depressive disorder and schizophrenia cases.[165]

*KAZN (*Kazrin, periplakin interacting protein) codes for a highly conserved protein that is noted for being important in the formation of the cornified envelope of keratinocytes but expression throughout the body suggests it has other important developmental functions.[166] *KAZN* is moderately expressed in the brain [155,167], where it has been found to be underexpressed in parvalbumin neurons of the superior temporal cortex of schizophrenia cases.[168] Notably, dysfunction of PV interneurons is casually linked to many mental disorders including PTSD.[169] KAZN is overexpressed in the substantia nigra of Parkinson's cases.[170]

TMEM51-AS1 (transmembrane protein 51 antisense) is the non-coding antisense transcript to TMEM51. No additional information regarding their role in the human or rodent brain is available.

The C2H2 zinc-finger binding motif of *ZNF813* (zinc finger protein 813) and the predicted nuclear location of its product suggest a role in DNA binding and transcriptional activity, however, its function in vitro and in-vivo remains unclear. It is weakly expressed in the human brain.[155]

Gene-based analyses in EUA further identified 2 genes.

SH3RF3 (SH3 Domain Containing Ring Finger 3): encodes a protein that contains four Src homology 3 domains as well as a RING finger domain that confers E3 ubiquitin ligase activity[171]. Src homology 3 domains mediate protein-protein interactions[172] and E3 ubiquitin ligase genes are important in development of neurological diseases.[173,174] SH3RF3 is ubiquitously expressed, with moderate expression seen in the frontal cortex.[155,167] A linkage study of Alzheimer's disease found an association between *SH3RF3* and disease age of onset [175].

PODXL (Podocalyxin-like protein 1) encodes a protein involved in regulation of cellular adhesion and morphology, that is particularly noted for being important in kidney development and abnormally expressed in a variety of cancers.[176] In the brain, PODXL is expressed in microvessels and may be important in the formation or function of the blood brain barrier.[177] Genomics studies have implicated *PODXL* in several disorders including alcoholism[178], Alzheimer's disease [179], bipoar disorder[180], epilepsy[181], and schizophrenia.[182]

For our top AFA association, FUMA mapped the SNP to *LINC02335, MIR5007,* and *TUC338*. There is little information available about the functional role of any of these RNAs. *MIR5007* was marginally associated with eye movement phenotypes in related to psychotic disorders ($P = 3 \times 10^{-7}$).[183]

The top SNP in AFA males was mapped to *LINC02571* and *HLA-B (*Major Histocompatibility Complex, Class I, B) in the human leukocyte antigen region. This region plays a major role in immune function and has repeatedly been associated with psychiatric illnesses.[184,185] Lead SNP rs142174523 is a putative pleiotropic SNP for rheumatoid arthritis and schizophrenia.[186] It is ~700KB away from the most significant schizophrenia hit[187], but localization of functional variants in this region is notably difficult and can require extensive follow up.

Functional annotation of variants in risk loci

FUMA[188] was also used for functional annotation of variants in the 6 genome-wide significant regions (see id's 30-33 at [https://fuma.ctglab.nl/browse]). For the first EUA locus (Supplementary Figure 24, Table 4), one SNP (rs35262389) in LD with the lead SNP rs34517852 has a Combined Annotation-Dependent Depletion (CADD) score of 15.28 that may be indicative of this being the functional variant of this locus. Another SNP (rs9348095) in LD has a CADD score of 9.498 and RegulomeDB indicates that this variant is located in the TSS of ZDHHC14, potentially influencing its transcriptional activity. Hi-C data further indicated that the risk locus, which is upstream of *ZDHHC14,* interacts with elements further downstream in *ZDHHC14*, potentially regulating transcriptional activity.

For the second EUA locus (lead SNP rs9364611), chromatin state analysis showed that most of the locus is transcriptionally quiescent, however, a few SNPs are located in enhancer sites or weakly transcribed regions when tested in across neuronal cell lines/tissues. Hi-C data show further that the risk locus forms physical contact with another site in the same intron of *PARK2*; however, the functional implications of this interaction remain unknown.

No variants had significant or relevant functional evidence in the top locus identified in the EUA males (lead SNP rs571848662).

For the second locus (lead SNP rs148757321), chromatin state analyses showed only a weak transcription signal across all neuronal cell lines/tissues. Hi-C chromatin interaction data show significant interactions with regions upstream of *KAZN* without further evidence for a functional role of this interaction.

The AFA locus with lead SNP rs115539978 contained a few SNPs associated with heterochromatin and enhancer function. Hi-C chromatin interaction data showed significant chromatin conformation interactions between the risk region and a region approximately 1,100kb upstream harboring additional non-coding RNAs including LINC00458, hsa-mir-1297 and LINC00558 as well as a region approximately 820kb downstream harboring the pseudogene *HNF4GP1*.

For the locus in AFA males with lead SNP rs142174523, RegulomeDB scores indicate potential regulatory functions of SNPs in the locus on transcription factor binding and eQTL function. This is in line with the chromatin state analyses that show some heterochromatin enrichment across all neuronal cell lines/tissues as well as stretches of PolyComb repressed chromatin. Furthermore, eQTL analyses did show significant associations with gene expression in *ATP6V1G2, C4A, C4B, CCHCR1, CYP21A1P, DDR1, HCG27, HLA-B, HLA-C, MICB, NOTCH4, POU5F1, PSORS1C3,*

SKIV2L, VARS, and *VARS2* across the different eQTL databases used.

Regulation of non-coding RNAs by the African ancestry specific GWAS hit rs115539978

To gain deeper insight into the function of rs115539978 on expression of non-coding RNAs at this locus we used 12 lymphoblastoid cell lines (LCLs) from the AFR superpopulation of the 1000 Genomes Project (Supplementary Table 13).

Prior evidence suggests that hsa-mir-5007 may not be an expressed miRNA[167,189] and RNA-seq data from our lab in peripheral blood and post-mortem brain tissue did not reveal expression of this particular miRNA (data not shown). However, using a qPCR approach we detected expression of Linc00458 and TUC338 (Supplementary Table 14).[190] Linc02335 was weakly expressed and Linc00558 was not expressed in the LCLs used.

We next tested if rs115539978 genotype influences differential expression of Linc00458, TUC338 and Linc02335 under basal conditions, however, we did not observe expression differences for any of the RNAs tested (Linc00458: $F(1,9) = 1.452$, $P = 0.259$; TUC338: $F(1,9) = 2.828$, $P = 0.127$; Linc02335: $F(1,9) = 0.036$, $P = 0.854$).

We next tested the hypothesis that genotype dependent differences in RNA expression may emerge only after in-vitro activation of the glucocorticoid receptor, using dexamethasone representing a pharmacological stress-exposure condition. Of note, Linc02335 has been previously described as a Dex responsive gene in airway smooth muscle cells.[191] Indeed, genotype dependent differences in RNA expression emerged after 4 hours Dex stimulation for Linc00458 and TUC338 with a downregulation of these RNAs in cells carrying the major allele compared to the minor (risk) allele (Linc00458: $F(1,9) = 5.328$, $P = 0.046$; TUC338: $F(1,9) = 5.425$, $P = 0.045$) (Supplementary Figure 25 B and D). Similar differences were observed for TUC338 in ethanol vehicle control experiments but not for Linc00458 (UC338: $F(1,9) = 7.940$, $P = 0.02$; Linc00458: $F(1,9) = 1.571$, $P = 0.242$) (Supplementary Figure 25 A and C). Thus, rs115539978 may influence the expression of non-coding RNAs in response to increased glucocorticoid receptor signaling, thus linking this African-specific genetic variant to stress response and non-coding RNA expression. The biological roles of both TUC338 and Linc00458 in the brain remain unknown. Linc00458 (BC026300) was previously described as an exclusive transcript in human embryonic stem cells, and implicated in the regulation of pluripotency via direct interaction with SOX2.[190] The human genome contains several copies of TUC338. Although a TUC338 transcript from chromosome 12 has been assigned a functional role in hepatocellular carcinoma cell growth[192], it remains unclear if other TUC338 copies share similar functions. To date, no brain-specific role for TUC338 has been described.

Deep phenotyping exploration of the African ancestry top hit rs115539978

To explore the African-specific lead variant in more detail we tested the hypothesis that rs115539978 is associated with intermediate phenotypes previously associated

with PTSD, including functional imaging phenotypes and psychophysiology. PGC-PTSD includes a large number of extensively phenotyped studies, and working groups across multiple biological systems are focused on implementing pipelines for functional follow-up of genomic regions of interest.[193] Data were used from a subsample of the Grady Trauma Project (study #47, GTPC), where individual genetic data and detailed phenotypic data were readily available. GTPC is the largest PGC2 AFA dataset (Supplementary Table 5) and shows significant association of rs115539978 with PTSD diagnosis ($P < 4.55 \times 10^{-7}$, Supplementary Figure 14).

Neuroimaging: Examining brain structure (size) using freesurfer, we examined amygdala bilateral average volume in rs115539978 CC genotype vs. T carriers. We found greater amygdala volume in the rs115539978 T-carriers (1689uL, $N = 14$), relative to subjects homozygous for the C-allele (1549uL, $N = 73$), $F(1,87) = 5.0$, $P = 0.03$ (Supplementary Figure 26 A). Although there is evidence for an overall reduction of amygdala volume in PTSD in some previous literature, the most robust effects are generally for hippocampal volume ([194]). Furthermore, larger amygdala volumes and enhanced functional connectivity were reported with increases in fear- and anxiety related symptoms [195]. Finally, there is a long history of increase dendritic arborization and amygdala volume in animal models of chronic stress.[196]

Psychobiology: We examined the role of rs115539978 in association with fear potentiated startle habituation – a physiological phenotype known to be, in part, amygdala dependent.[197] We found a significant effect of rs115539978 genotype on habituation to startle CS+ (from block 2 to block 3 (ACQ_HAB)), a variable that captures habituation to the conditioned aversive stimulus. The main effect of SNP is significant at $P = 0.048$ (N=248 CC and 51 T carriers) comparing the CC to CT genotype (TT-carriers are not available). The significance is maintained ($P = 0.044$) if we covary for demographic variables (age and sex) (Supplementary Figure 26 B). Thus, this lead SNP seems to capture a genomic region that regulates ncRNA expression related to stress response and is also associated with increased amygdala volume and startle psychophysiology in a traumatized population.

Supplementary Note 2

Acknowledgements

Army Study to Assess Risk and Resilience in Servicemembers (NSS1, NSS2, PPDS)(T1#14, T1#15, T1#16)

Funding:

Army STARRS was sponsored by the Department of the Army and funded under cooperative agreement number U01MH087981 (2009-2015) with the National Institutes of Health, National Institute of Mental Health (NIH/NIMH). Subsequently, STARRS-LS was sponsored and funded by the Department of Defense (USUHS grant number HU0001-15-2-0004). The contents are solely the responsibility of the authors and do not necessarily represent the views of the Department of Health and Human Services, NIMH, the Department of the Army, or the Department of Defense.

The Army STARRS Team consists of:

Co-Principal Investigators: Robert J. Ursano, MD (Uniformed Services University of the Health Sciences) and Murray B. Stein, MD, MPH (University of California San Diego and VA San Diego Healthcare System)

Site Principal Investigators: Steven Heeringa, PhD (University of Michigan), James Wagner, PhD (University of Michigan) and Ronald C. Kessler, PhD (Harvard Medical School)

Army liaison/consultant: Kenneth Cox, MD, MPH (US Army Public Health Center)

Other team members: Pablo A. Aliaga, MS (Uniformed Services University of the Health Sciences); COL David M. Benedek, MD (Uniformed Services University of the Health Sciences); Susan Borja, PhD (NIMH); Tianxi Cai, ScD (Harvard School of Public Health); Laura Campbell-Sills, PhD (University of California San Diego); Chia-Yen Chen, ScD (Harvard Medical School); Carol S. Fullerton, PhD (Uniformed Services University of the Health Sciences); Nancy Gebler, MA (University of Michigan); Joel Gelernter, MD (Yale University); Robert K. Gifford, PhD (Uniformed Services University of the Health Sciences); Feng He, MS (University of California San Diego); Paul E. Hurwitz, MPH (Uniformed Services University of the Health Sciences); Sonia Jain, PhD (University of California San Diego); Kevin Jensen, PhD (Yale University); Kristen Jepsen, PhD (University of California San Diego); Tzu-Cheg Kao, PhD (Uniformed Services University of the Health Sciences); Lisa Lewandowski-Romps, PhD (University of Michigan); Holly Herberman Mash, PhD (Uniformed Services University of the Health Sciences); James E. McCarroll, PhD, MPH (Uniformed Services University of the Health Sciences); Adam X. Maihofer (University of California San Diego); Colter Mitchell, PhD (University of Michigan); James A. Naifeh, PhD (Uniformed Services University of the Health Sciences); Tsz Hin Hinz Ng, MPH (Uniformed Services University of the Health Sciences); Caroline M. Nievergelt, PhD (University of California San Diego); Matthew K. Nock, PhD (Harvard University); Stephan Ripke, MD (Harvard Medical School); Nancy A. Sampson, BA (Harvard Medical School); CDR Patcho Santiago, MD, MPH (Uniformed Services University of the Health Sciences); Ronen Segman, MD

(Hadassah University Hospital, Israel); Jordan W. Smoller, MD, ScD (Harvard Medical School); Xiaoying Sun, MS (University of California San Diego); Erin Ware PhD (University of Michigan); LTC Gary H. Wynn, MD (Uniformed Services University of the Health Sciences); Alan M. Zaslavsky, PhD (Harvard Medical School); and Lei Zhang, MD (Uniformed Services University of the Health Sciences).

Ash Wednesday and IVS (BRYA)(T1#10)
This project was funded by a National Health and Medical Research Council Grant (1073041).

Bounce Back Now (BOBA)(T1#18)
This work was supported by 1R01MH081056 (PI: Ruggiero), 1R01MH081056-S1 (PI: Amstadter), as well as K02 AA023239 (PI: Amstadter) and K01 AA025692 (PI: Sheerin).

Childhood Trauma Study (QIMR) (T1#30)
This work was primarily supported by National Institute of Health grants to ECN (AA13446; AA011998_5978). Additional support includes grants to ACH (AA10249, AA07728, AA11998, AA13321), NGM (AA13326), PAFM (DA12854; DA027995).

Child Trauma and Neural Systems Underlying Emotion Regulation (KMCT)(T1#19)
This work was funded by the R01-MH103291 (PI: McLaughlin), R01-MH103291-S1 (PI: McLaughlin), and R01-MH103291-S2 (PI: McLaughlin).

Cohen Veterans Center Study and Fort Campbell study (COM1, FTCB)(T1#50, T1#52)
These studies were supported by the Steve and Alexandra Cohen Foundation and the Department of Defense (DoD: W81XWH-09-2-0044 to C.R.M. and W911NF-09-1-0298 to R.Y.).

Cortical Excitability: Biomarker and Endophenotype in Combat Related PTSD (WANG)(T1#59)
This work was supported by VA Merit Review awardS 1I21RX001725-01, 1I01CX000487-01A1, and 1R34MH078854-01.

Danish military study (DAMI)(T1#28)
This research has been conducted using the Danish National Biobank resource, supported by the Novo Nordisk Foundation. The study was supported by the Research and Knowledge Centre, The Danish Veteran Centre and funded by the Danish Ministry of Defence as part of the 3rd September 2014 agreement on strengthened initiatives for Danish veterans.

Danish iPSYCH PTSD samples (DAIP)(T1#29)
The iPSYCH team acknowledges funding by the Lundbeck Foundation (grant numbers R102-A9118 and R155-2014- 1724) and the universities and university hospitals of Aarhus and Copenhagen. The Danish National Biobank resource was supported by the Novo Nordisk Foundation. Data handling and analysis on the GenomeDK HPC facility was supported by NIMH (1U01MH109514-01 to Michael O'Donovan and ADB). High-performance computer capacity for handling and statistical analysis of iPSYCH data on the GenomeDK HPC facility was provided by the Centre for Integrative Sequencing, iSEQ, Aarhus University, Denmark (grant to ADB).

DCS Rothbaum Study (DCSR)(T1#38)
For support, this study was funded by by NIMH grant R01 MH-70880 to Dr. Rothbaum, Clinicaltrials.gov identifier: NCT00356278**.**

Defining Essential Features of Neural Damage (DEFE)(T1#12)
Congressionally Directed Medical Research Programs, W81XWH-08–2–0038
Department of Veteran Affairs Rehabilitation Research and Development Service, 1IK1RX002325, I01RX000622

Detroit Neighborhood Health Study (DNHS, ADNH)(T1#4, T1#45)
DNHS was funded by NIH Awards R01DA022720, R01DA022720-S1, and RC1MH088283 to Allison E. Aiello; and R01MD011728 to Monica Uddin. We are grateful to all of the participants and staff for their contributions to the DNHS.

Drakenstein Child Health Study - South African sample (SAFR)(T1#3)
Research reported in this publication was supported by the South African Medical Research Council (SAMRC) Unit on Risk & Resilience in Mental Disorders and a Self-Initiated Research Grant (NK). The views and opinions expressed are those of the authors and do not necessarily represent the official views of the SAMRC.

EA CRASH (EACR)(T1#42)
EA CRASH was supported by the National Institute of Arthritis and Musculoskeletal and Skin Diseases of the National Institutes of Health under Award Number R01-AR056328. The content is solely the responsibility of the authors and does not necessarily represent the views of this funding agency.

Family Study of Cocaine Dependence and Collaborative Genetic Study of Nicotine Dependence (FSCD, COGA, COGB)(T1#7, T1#8, T1#9)
The Collaborative Genetic Study of Nicotine Dependence (COGEND) was supported by National Cancer Institute grant P01CA089392 to Laura Bierut. The Family Study of Cocaine Dependence (FSCD) was supported by National Institute on Drug Abuse grants R01DA013423 and R01DA019963 to Laura Bierut. Funding support for genotyping was provided by the NIH GEI (U01HG004438), the National Institute on

Alcohol Abuse and Alcoholism, the National Institute on Drug Abuse, and the NIH contract "High throughput genotyping for studying the genetic contributions to human disease" (HHSN268200782096C).

GMRF-QUT (GMFR)(T1#55)

The Queensland Branch of the Returned and Services League of Australia (RSL) funded the PTSD Initiative at the Gallipoli Medical Research Institute. The Australian Government Department of Veterans' Affairs provided transport for eligible participants. We gratefully acknowledge the dedicated efforts of the participants and their families, and the clinical and support staff involved in data collection. Specifically, we would like to thank the members of the PTSD Initiative team: Sarah McLeay, PhD, Wendy Harvey, MPH, Madeline Romaniuk, DPsych(Clin), Darrell Crawford, MD, David Colquhoun, MBBS, Ross McD Young, PhD, Miriam Dwyer, BSc, John Gibson, MBBS, Robyn O'Sullivan, MBBS, Graham Cooksley, MBBS, Christopher Strakosch, MD, Rachel Thomson, PhD, Joanne Voisey, PhD, Bruce Lawford, MBBS. We would also like to thank Emile Touma and Vikram Goel for performing psychiatric assessments, Terence Harvey for developing the study database and QUT for financial support.

Grady Trauma Project (EGHS, GTPC)(T1#44, T1#47)

This study was supported by the National Institutes of Health, MH071537 and MH096764.

Injury and Traumatic Stress Consortium (INTR)(T1#27)

The PTSD and TBI INjury and TRaUmatic STress Clinical Consortium (INTRuST) was funded by a grant from the United States Department of Defense (PI: Stein, MB): W81XWH08-2-0159. Members of the INTRuST Consortium Biorepository Working Group who contributed to this work include: Gerald A. Grant MD, Christine E. Marx MD, Mark S. George MD, Thomas W. McAllister MD, Norberto Andaluz MD, Lori Shutter MD, Raul Comibra MD, Ross D. Zafonte DO, Sonia Jain PhD, Xue-Jun Qin, and Michael Hauser PhD.

Marine Resiliency Study (MRSC, BAKE)(T1#1,T1#57)

The Marine Corps, Navy Bureau of Medicine and Surgery (BUMED) and VA Health Research and Development (HSR&D) provided funding for MRS data collection and analysis (PI DGB) and NIH R01MH093500 funded the GWAS assays and analysis (PI CMN). The study was supported by the Veterans Administration Center of Excellence for Stress and Mental Health (CESAMH). Acknowledged are Victoria B. Risbrough Ph.D (VA San Diego Healthcare System & UCSD), Mark A. Geyer (UCSD), Daniel T. O'Connor (UCSD), all MRS investigators, as well as the MRS administrative core and data collection staff. The authors also thank the Marine and Navy Corpsmen volunteers for their military service and participation in MRS.

McLean Trauma Sample (TEIC)(T1#39)
The Kaufman lab would like to thank the participants for making this research possible; the staff of the Hill Center for Women and Proctor House II, McLean Hospital; The work was supported by National Institute of Mental Health (NIMH) grant R21MH112956 to MLK, and NIMH fellowship grant F32MH109274 to LAML, the Anonymous Women's Health Fund to MLK, the O'Keefe Family Foundation to MLK, the Trauma Scholars Fund to MLK, and the Frazier Foundation Grant for Mood and Anxiety Research to KJR.
The Teicher Lab would like to thank all the participants for being a part of these studies and the staff of Developmental Biopsychiatry Research Program, McLean Hospital. The studies were supported by National Institute of Mental Health (NIMH) grant RO1 MH91391 and National Institute on Drug Abuse R01 DA17846 to MHT.

Mid-Atlantic Mental Illness Research Education and Clinical Center the study of Post-Deployment Mental Health Study (MIRE)(T1#26)
Preparation of this manuscript was supported by a Clinical Sciences Research and Development (CSR&D) Research Career Scientist Award (#11S-RCS-009) to Dr. Beckham, a CSR&D Career Development Award (#IK2 CX000525) to Dr. Kimbrel, and Biomedical and Laboratory Research and Development (BLR&D) Merit Award to Dr. Beckham from the U.S. Department of Veterans Affairs (VA). This work was also supported by the VA Mid-Atlantic Mental Illness Research, Education and Clinical Center (MIRECC), the Durham Veterans Affairs Medical Center, the VA Office of Mental Health Services, and the VA Office of Research and Development. The Mid-Atlantic MIRECC Workgroup contributors for this paper include: Mira Brancu, PhD, Patrick S. Calhoun, PhD, Eric Dedert, PhD, Eric B. Elbogen, PhD, John A. Fairbank, PhD, Robin A. Hurley, MD, Jason D. Kilts, PhD, Angela Kirby, MS, Christine E. Marx, MD, MS, Scott D. McDonald, PhD, Scott D. Moore, MD, PhD, Rajendra A. Morey, MD, MS, Jennifer C. Naylor, PhD, Treven C. Pickett, PsyD, Jared Rowland, PhD, Cindy Swinkels, PhD, Steven T. Szabo, MD, PhD, Katherine H. Taber, PhD., Larry A. Tupler, PhD, Elizabeth E. Van Voorhees, PhD, H. Ryan Wagner, Ph.D., Ruth E. Yoash-Gantz, PsyD. Dedert is funded by a Department of Veterans Affairs Clinical Science Research and Development Career Development Award (IK2CX000718). Naylor is funded by a Department of Veterans Affairs Rehabilitation Research and Development Career Development Award (1IK2RX000908). Van Voorhees is funded by a Department of Veterans Affairs Rehabilitation Research and Development Career Development Award (1K2RX001298). The views expressed in this article are those of the authors and do not necessarily reflect the position or policy of the Department of Veterans Affairs or the United States government.

National Centre for Mental Health (NCMH)(T1 #41)
This project was supported by the National Centre for Mental Health (NCMH). NCMH is funded by Welsh Government through Health and Care Research Wales.

National Health and Resilience in Veterans Study (NHRV; Supplementary Data 1 #13)

The National Health and Resilience in Veterans Study is supported by the U.S. Department of Veterans Affairs National Center for Posttraumatic Stress Disorder.

NIU Trauma Orcutt (NIUT)(T1#40)

This study was supported by the Joyce Foundation (Dr Orcutt) and NIH, HD049907and MH085436.

Nurses Health Study II (NHS2, NHSY)(T1#5)(T1#22)

NHSII PTSD Sub-Study was funded by National Institute on Mental Health awards RO1 MH093612, MH078928 to Karestan C Koenen. The NHSII cohort is funded in part by UM1 CA176726.

Ohio National Guard (ONGA)(T1#2)

The funding information for the Ohio Army National Guard cohort is:
Dept. of Army, Telecommunication and Advanced Technology Research Center (TATRC) Award #s W81XWH-15-1-0080 and W81XWH-10-1-0579 “Ohio Army National Guard Mental Health Initiative: Genetics of Risk and Resilience for Deployment-Related Stress Disorders”.

OPT and CHOICE (FEEN)(T1 #37)

This research is funded by the National Institute of Mental Health (NIMH; R01MH066347, R01MH066348) and the William T. Dahms, MD, Clinical Research Unit, funded under the Cleveland Clinical and Translational Science Award (UL1 RR024989).
Pfizer Inc. supplied the medication at no cost but had no input in the trial development, conduct, analysis, or interpretation. Drs. Zoellner, Roy-Byrne, Mavissakalian, and Feeny have no competing interests to disclose.

We would like to thank all participants, therapists, and psychiatrists involved in the study and acknowledge the vital contributions of study researchers and administrators in Seattle, Washington and Cleveland, Ohio. Specifically, we would like to thank the investigative team on the grants: Jason Doctor, Ph.D., Joshua McDavid, MD, Alice S. Friedman, MSN, ARNP, and Nora McNamara, MD. Afsoon Eftekhari, Ph.D., and Lisa Stines Doane, Ph.D. were integral in the implementation of this study. Edna Foa, Ph.D. and her team provided PE integrity ratings. We would like to acknowledge Susan Silva, Ph.D., Eric Youngstrom, Ph.D., Kevin King, Ph.D., and Andrew A. Cooper, Ph.D. for their statistical consultation and analyses.

Pregnancy Outcomes, Maternal and Infant Cohort Study (PROM)(T1#23)

This research was supported by awards from the Eunice Kennedy Shriver Institute of Child Health and Human Development (R01-HD-059835 and R01 HD059827).

Readiness and Resilience in National Guard Soldiers (RING)(T1#33)
Support for this study came from the Center for Veterans Research and Education.

Risbrough/Norman randomized controlled psychotherapy trial (VRIS)(T1#58)
Samples and data collection were funded by VA Office of Clinical Science Research and Development (5IO1CX000756 to SBN) and the VA Office of Basic Science Research and Development (1I01BX002558 to VR). Additional salary support was funded by the VA National Center for PTSD (Norman) and VA Center of Excellence for Stress and Mental Health (Risbrough).

Shared Roots (SHRS)(T1#46)
The Shared Roots project is supported by the South African Medical Research Council for the Shared Roots Flagship Project, Grant no. MRC-RFA-IFSP-01-2013/SHARED ROOTS through funding received from the South African National Treasury under its Economic Competitiveness and Support Package. Its contents are solely the responsibility of the authors and do not necessarily represent the official views of the South African Medical Research Council. Additional funding was received from the South African Research Chairs Initiative of the South African Department of Science and Technology and National Research Foundation.

Southeastern Europe PTSD (SEEP)(T1#49)
Recruitment of the SEE-PTSD cohort was funded by the DAAD.

STRONG STAR Genetic and Environmental Predictors of Combat-Related PTSD (STRO) (T1 #35)
Funding for the STRONG STAR sample collection was made possible by the U.S. Department of Defense through the U.S. Army Medical Research and Materiel Command, Congressionally Directed Medical Research Programs, Psychological Health and Traumatic Brain Injury Research Program awards W81XWH-08-02-109 (Alan Peterson) and W81XWH-08-02-0110 (Douglas Williamson), and W81XWH-08-02-0114 (Brett Litz).

Study of Aftereffects of Trauma: Understanding Response in National Guard (SATU)(T1#11)
Congressionally Directed Medical Research Programs, W81XWH-08–2–0038
Department of Veteran Affairs Rehabilitation Research and Development Service, 1IK1RX002325, I01RX000622

Sydney Neuroimaging (BRY2)(T1#36)
This project was funded by a National Health and Medical Research Council Grant (1073041).

UK Biobank (UKBB)(T1#60)
This research has been conducted using the UK Biobank Resource, as an approved extension to application 16577 (Dr. Breen). This study represents independent research part funded by the National Institute for Health Research (NIHR) Biomedical Research Centre at South London and Maudsley NHS Foundation Trust and King's College London. The views expressed are those of the authors and not necessarily those of the NHS, the NIHR or the Department of Health and Social Care. High performance computing facilities were funded with capital equipment grants from the GSTT Charity (TR130505) and Maudsley Charity (980). GB and JRIC acknowledge funding from Cohen Veterans Bioscience.

VA Boston-National Center for PTSD Study (NCPT, TRACT)(T1#31, T1#32)
This research was supported in part by National Institute of Mental Health Award RO1MH079806 (MWM), Department of Veterans Affairs, Clinical Science Research & Development Program Award 5I01CX000431-02 (MWM), Department of Veterans Affairs, Biomedical Laboratory Research & Development Program Award 1I01BX002150-01 (MWM), The Translational Research Center for TBI and Stress Disorders (TRACTS), A VA Traumatic Brain Injury National Network Rehabilitation Research and Development Center award B9254-C (R.E.M. and W.P.M.), Department of Veterans Affairs. This research is the result of work supported with resources and the use of facilities at the Pharmacogenomics Analysis Laboratory, Research and Development Service, Central Arkansas Veterans Healthcare System, Little Rock, Arkansas. This work was also supported by a Career Development Award to E. J. Wolf from the Department of Veterans Affairs, Clinical Sciences Research, and Development Program.

Vietnam Era Twin Study of Aging (VETS) (T1#24)
This research was supported by National Institute on Aging R01 AG018386, AG022982, AG050595 (W.S.K.), R01 AG018384 (M.J.L.), R03 AG046413 (C.E.F), and K08 AG047903 (M.S.P), and the VA San Diego Center of Excellence for Stress and Mental Health Healthcare System. The content is the responsibility of the authors and does not necessarily represent official views of the NIA, NIH, or VA. The Cooperative Studies Program of the U.S. Department of Veterans Affairs provided financial support for development and maintenance of the Vietnam Era Twin Registry. We would also like to acknowledge the continued cooperation and participation of the members of the VET Registry and their families.

The Women and Children's Health Study (WACH)(T1#43)
Funding: This study was primarily supported by the National Institute of Environmental Health Sciences (grant 1U01ES021497)

Yale-Penn Study (GSDC)(T1#6)
This study was supported by National Institutes of Health Grants RC2 DA028909, R01 DA12690, R01 DA12849, R01 DA18432, R01 AA11330, and R01 AA017535

and the Veterans Affairs VISN 1 and VISN 4 Mental Illness Research, Educational, and Clinical Centers; and the VA National Center for PTSD Research.

Genotyping services for a part of our genome-wide association study were provided by the Center for Inherited Disease Research and the Yale Center for Genome Analysis. Center for Inherited Disease Research is fully funded through a Federal contract from the National Institutes of Health to The Johns Hopkins University (contract number N01-HG-65403).

Million Veteran Program (MVP) (replication cohort)

This research includes data from the Million Veteran Program (MVP), Office of Research and Development, Veterans Health Administration, and was supported by MVP and the VA Cooperative Studies Program (CSP) study #575B.

Supplementary Figures

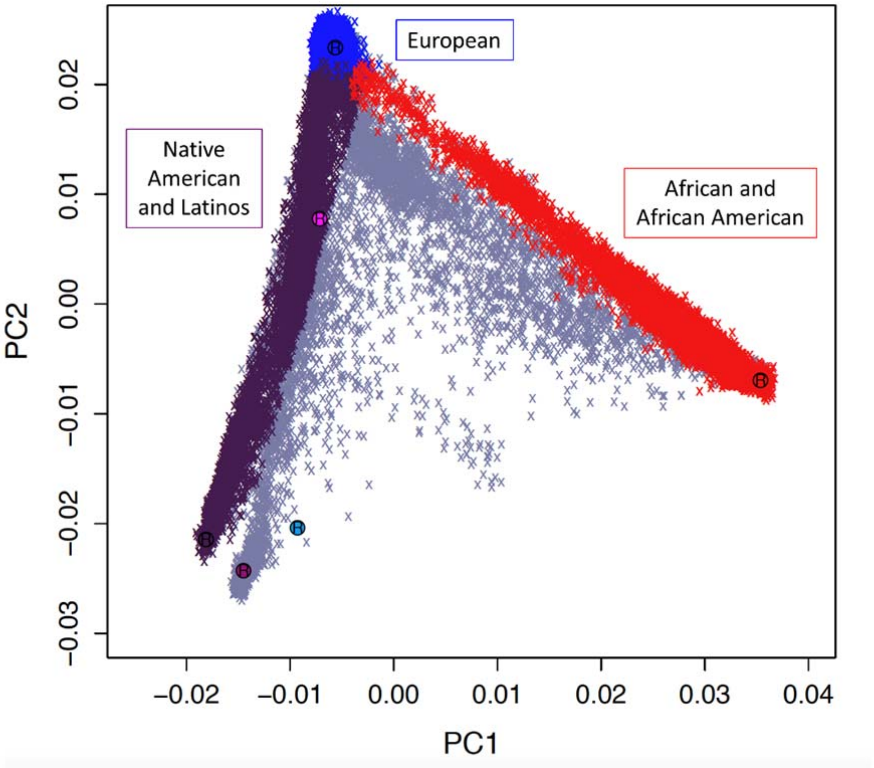

Supplementary Figure 1: Standardized global ancestry grouping for PGC-PTSD Freeze 2 GWA studies.

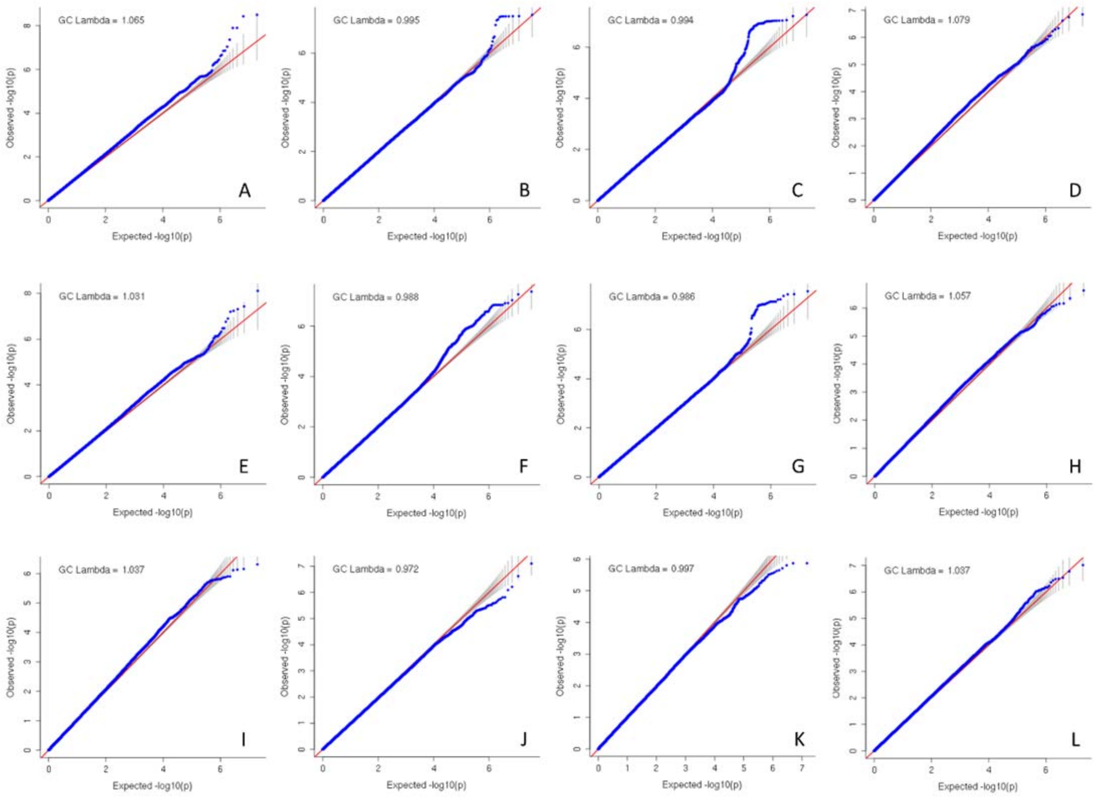

Supplementary Figure 2: Quantile-quantile plots of expected versus observed -log$_{10}$ *p*-values for genome-wide association studies (GWAS) with PTSD in subjects of different ancestries.

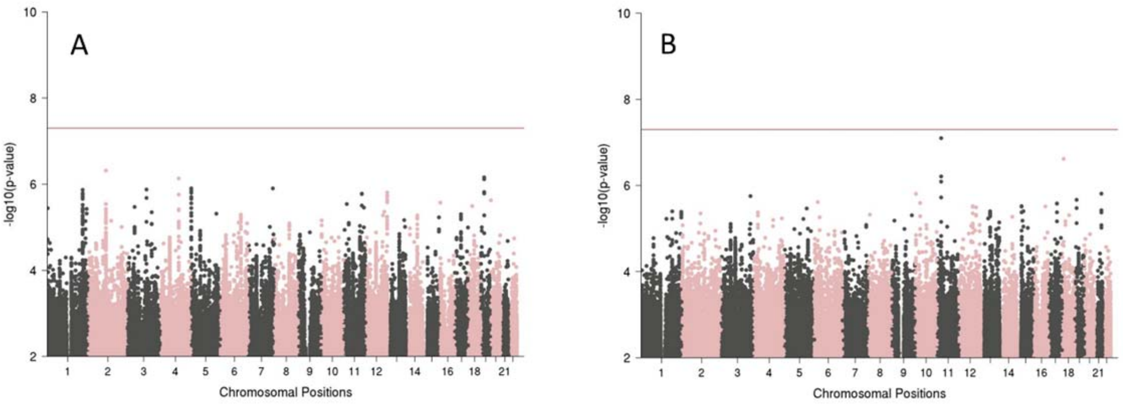

Supplementary Figure 3: Manhattan plots from meta-analyses of GWAS in women showing no genome-wide significant loci in subjects of A) European and B) African ancestry. The red line indicates the genome-wide significance threshold at $P < 5 \times 10^{-8}$.

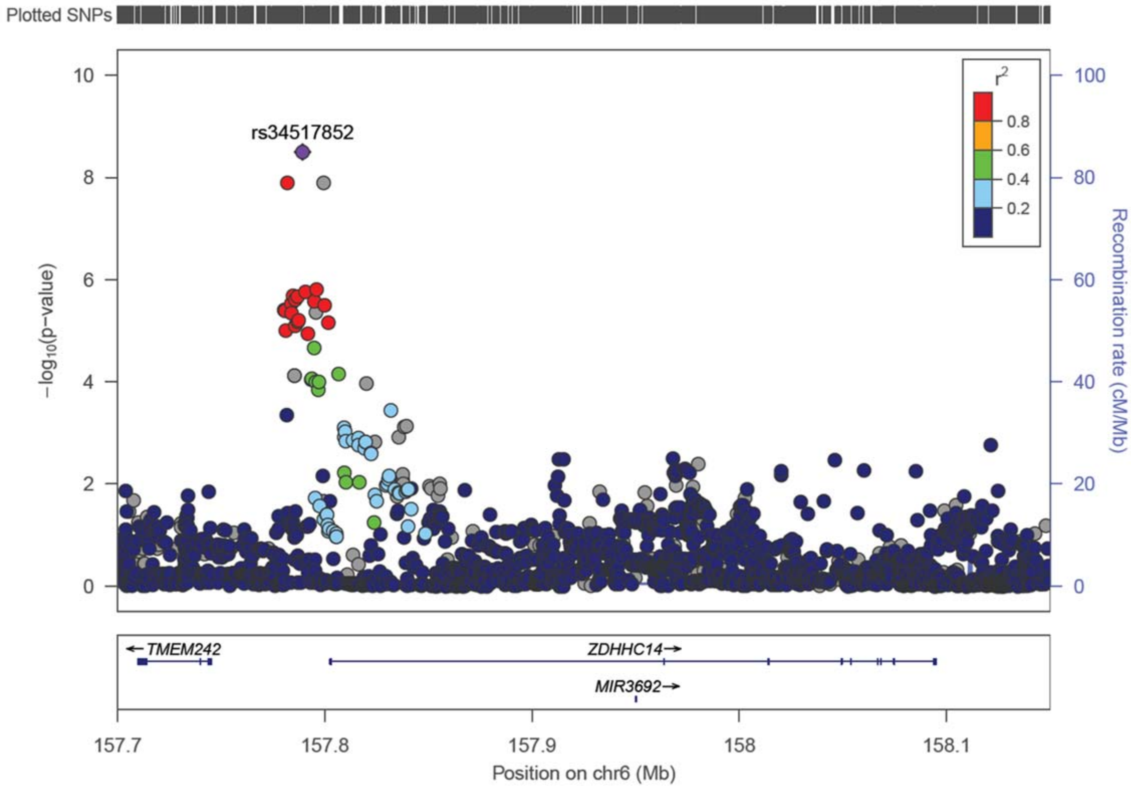

Supplementary Figure 4: Regional association plots (Locus zoom) of the European ancestry top hit rs34517852 in the EUA meta-analysis. Chromosomal position is indicated on the x-axis, -log10 *P*-values for each SNP (filled circles) is indicated on the y-axis, with the lead SNP shown in purple. Annotated genes in the region are drawn in the lower panel. Recombination rate is indicated by a blue line. Additional SNPs in the locus are colored according to linkage disequilibrium (r^2) with the lead SNP.

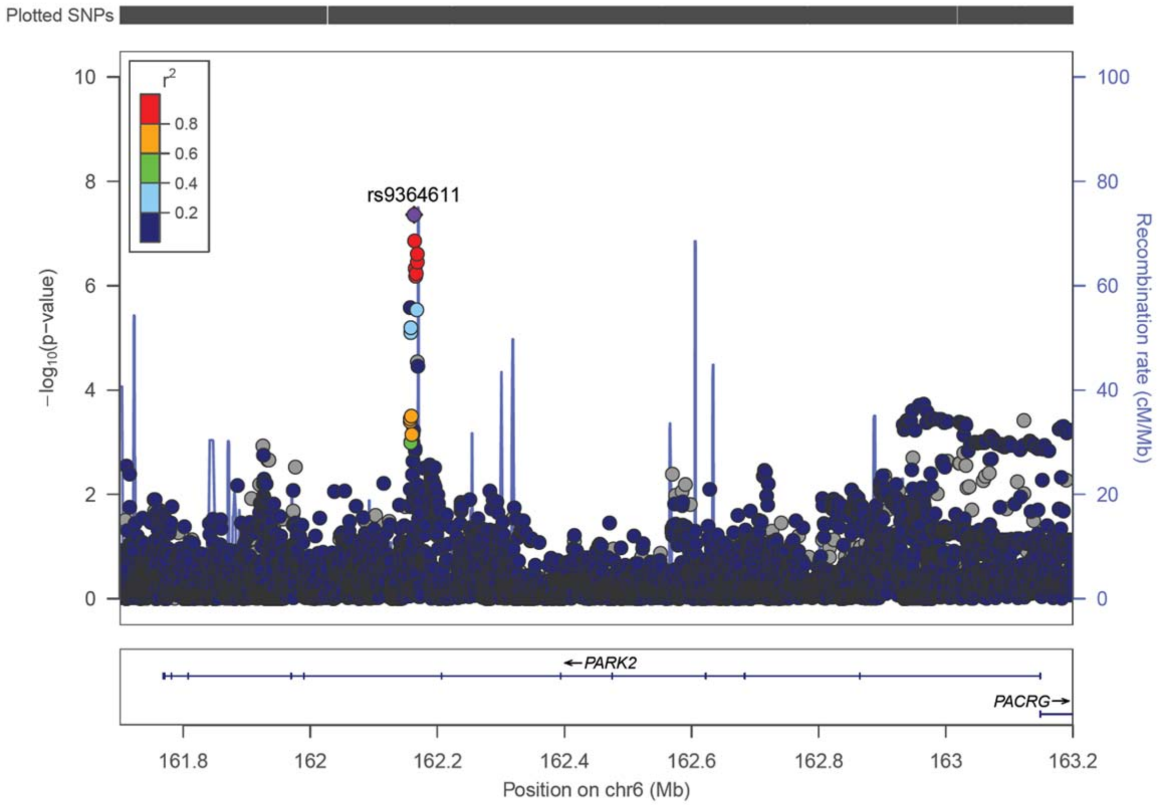

Supplementary Figure 5: Regional plots (Locus zoom) of the European ancestry second hit rs9364611 in the EUA meta-analysis. Chromosomal position is indicated on the x-axis, -log10 *P*-values for each SNP (filled circles) is indicated on the y-axis, with the lead SNP shown in purple. Annotated genes in the region are drawn in the lower panel. Recombination rate is indicated by a blue line. Additional SNPs in the locus are colored according to linkage disequilibrium (r^2) with the lead SNP.

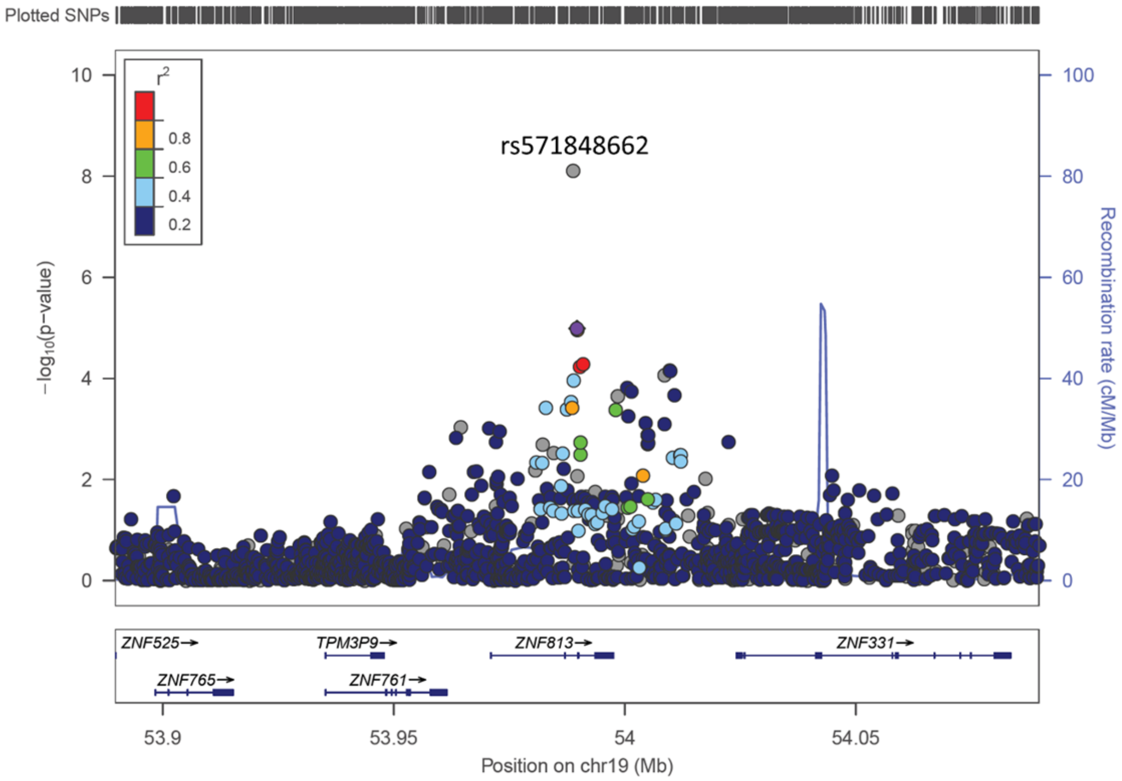

Supplementary Figure 6: Regional plots (Locus zoom) of the European ancestry male top hit rs571848662 (gray circle) in the EUA sex-stratified meta-analysis. Chromosomal position is indicated on the x-axis, -log10 *P*-values for each SNP (filled circles) is indicated on the y-axis, with the lead SNP shown in purple. Annotated genes in the region are drawn in the lower panel. Recombination rate is indicated by a blue line. Additional SNPs in the locus are colored according to linkage disequilibrium (r^2) with the lead SNP. The second most significant hit in the region, rs8112292, is marked as target variant (purple circle) for r^2 estimates in LocusZoom because the reference panel for LD calculations does not include rs571848662.

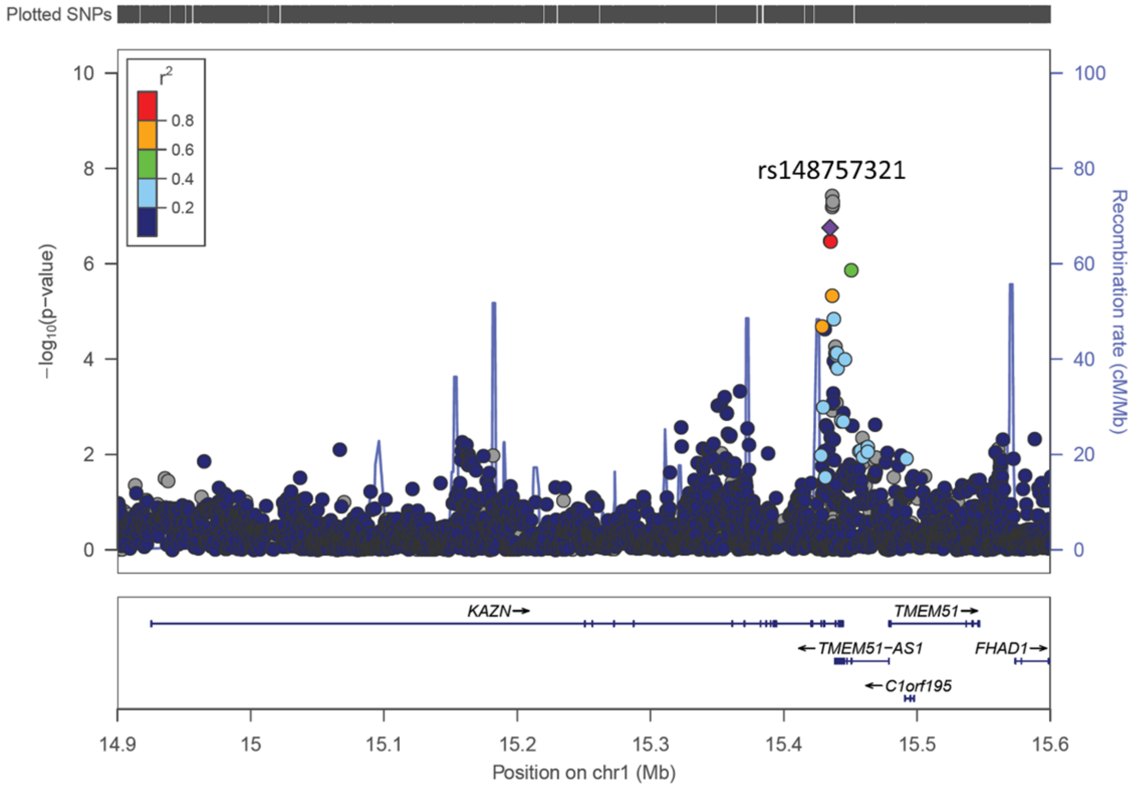

Supplementary Figure 7: Regional plots (Locus zoom) of the European ancestry male second hit rs148757321 (gray circle) in the EUA sex-stratified meta-analysis. Chromosomal position is indicated on the x-axis, -log10 *P*-values for each SNP (filled circles) is indicated on the y-axis, with the lead SNP shown in purple. Annotated genes in the region are drawn in the lower panel. Recombination rate is indicated by a blue line. Additional SNPs in the locus are colored according to linkage disequilibrium (r^2) with the lead SNP. The second most significant hit in the region, rs514370, is marked as target variant (purple diamond) for r^2 estimates in LocusZoom because the because the reference panel for LD calculations does not include rs148757321.

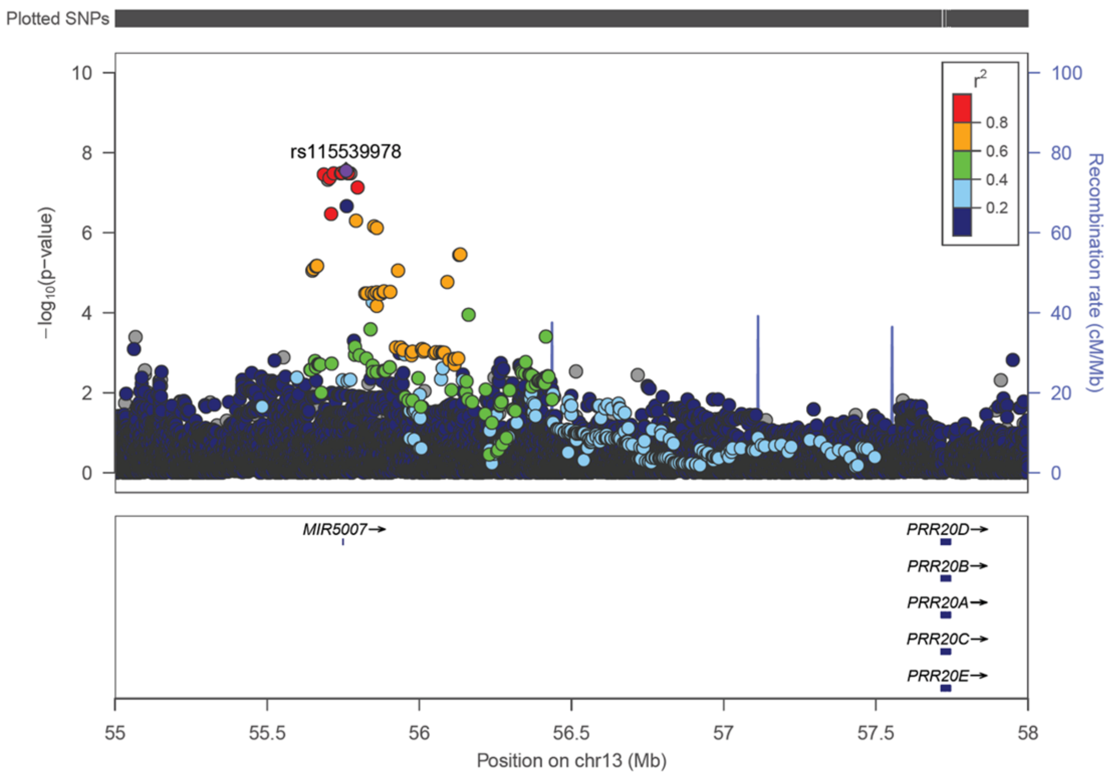

Supplementary Figure 8: Regional plots (Locus zoom) of the African ancestry top hit rs115539978 (purple diamond) in the AFA meta-analysis. Chromosomal position is indicated on the x-axis, -log10 *P*-values for each SNP (filled circles) is indicated on the y-axis, with the lead SNP shown in purple. Annotated genes in the region are drawn in the lower panel. Recombination rate is indicated by a blue line. Additional SNPs in the locus are colored according to linkage disequilibrium (r^2) with the lead SNP.

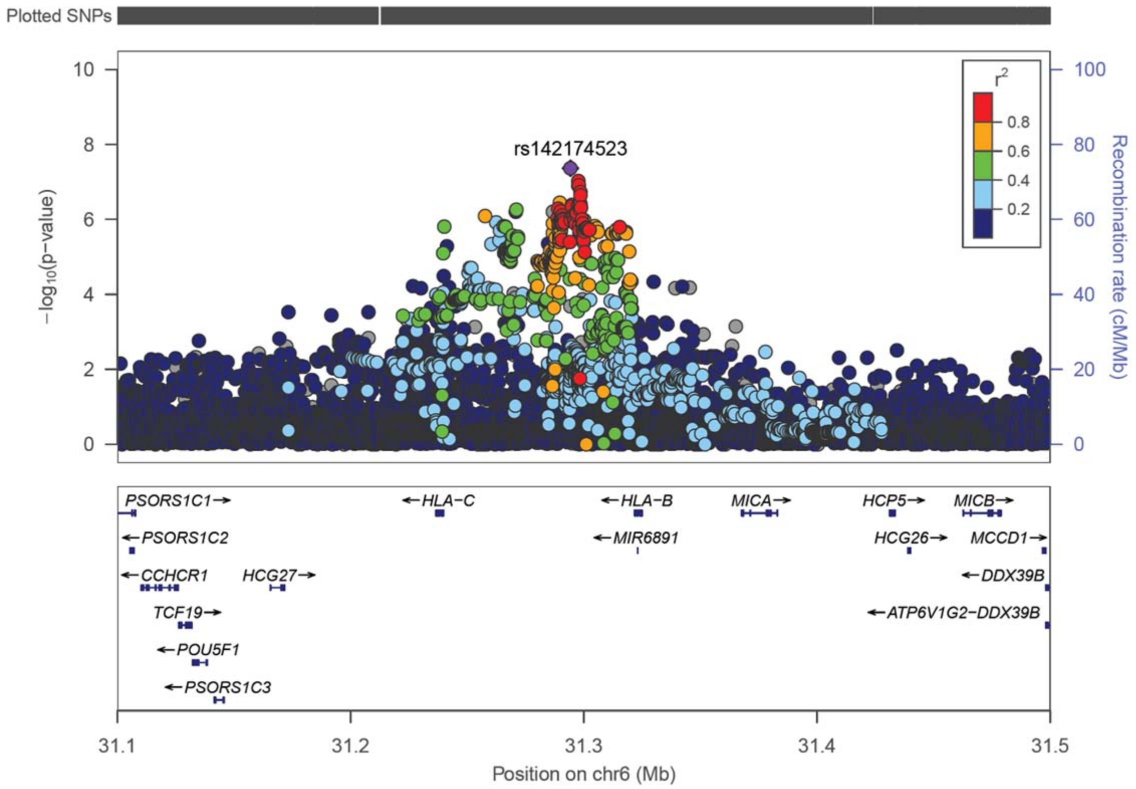

Supplementary Figure 9: Regional plots (Locus zoom) of the African ancestry male top hit rs142174523 (purple diamond) in the AFA sex-stratified meta-analysis. Chromosomal position is indicated on the x-axis, -log10 *P*-values for each SNP (filled circles) is indicated on the y-axis, with the lead SNP shown in purple. Annotated genes in the region are drawn in the lower panel. Recombination rate is indicated by a blue line. Additional SNPs in the locus are colored according to linkage disequilibrium (r^2) with the lead SNP.

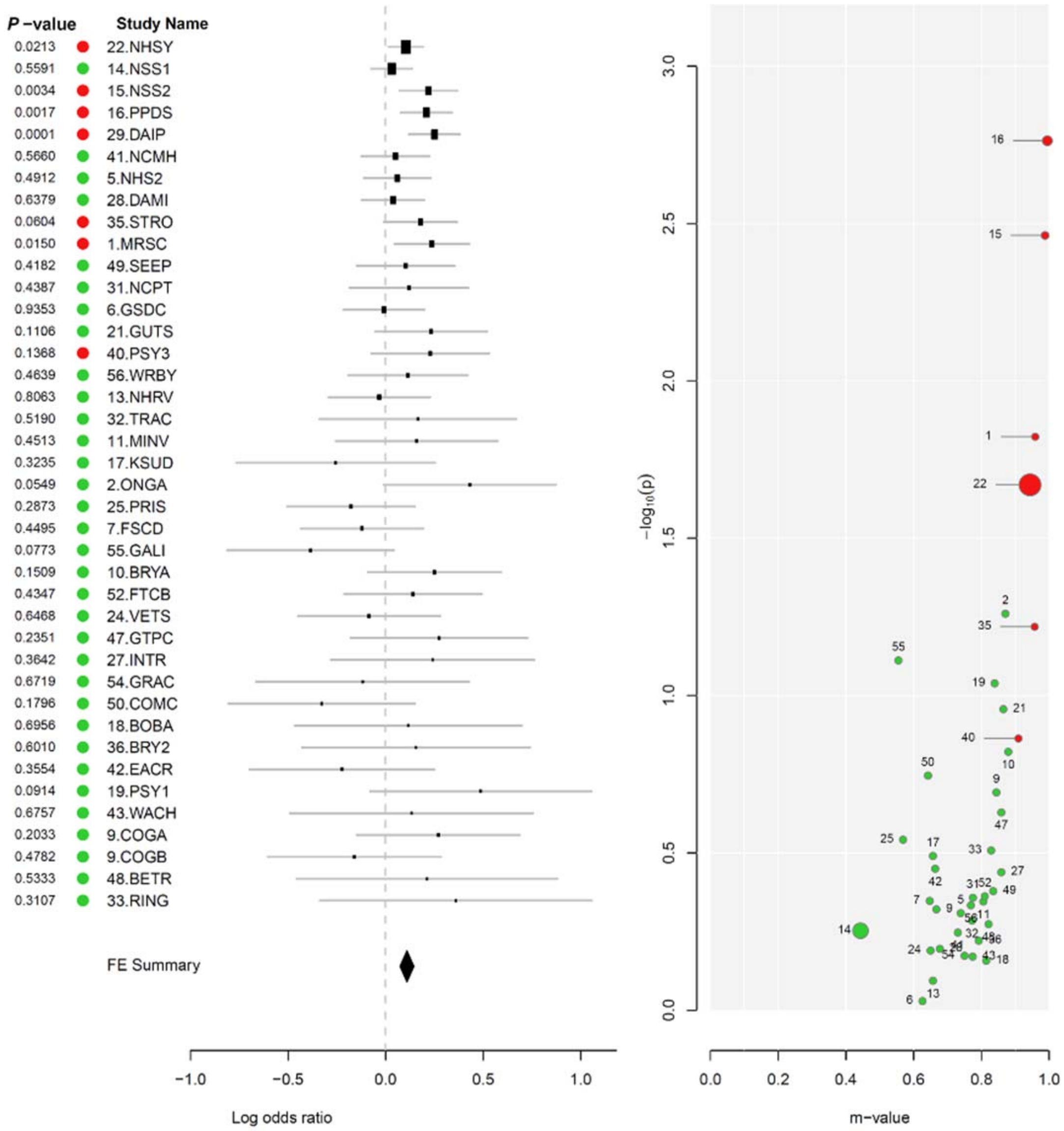

Supplementary Figure 10: Forest plot (left panel) of the European top hit rs34517852 (*P* = 3.16 x 10^{-9}) showing effect sizes for each of the 40 included GWAS in the EUA meta-analysis. The PM-Plot (right panel) is separating studies predicted to have an effect on PTSD (red) from underpowered studies with ambiguous effects (green) or predicted to have no effect (blue). For a detailed study description see Supplementary Methods and Supplementary Data 1.

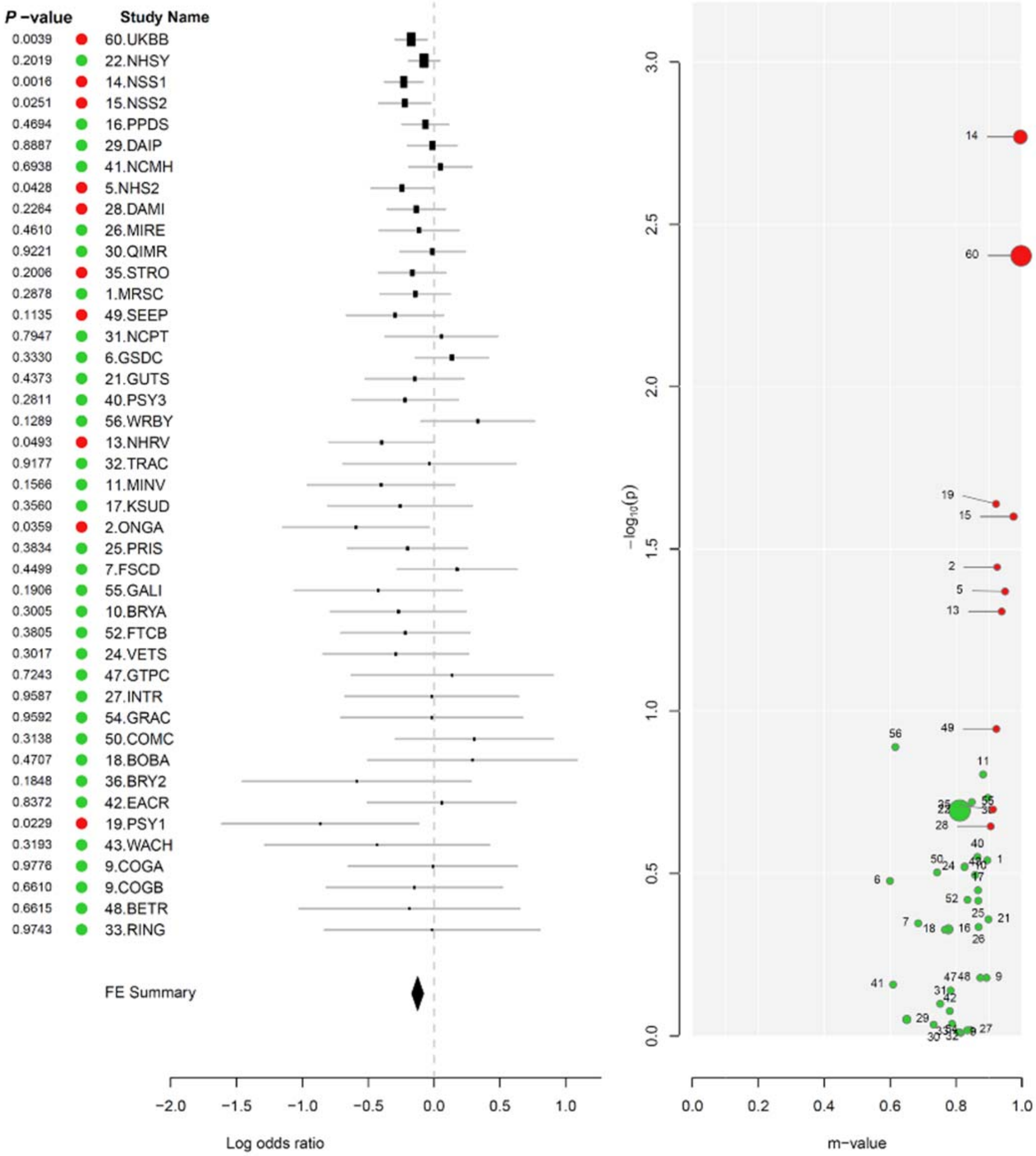

Supplementary Figure 11: Forest plot (left panel) of the European second hit rs9364611 (*P* = 4.36 x 10^{-8}) showing effect sizes for each of the 43 included GWAS in the EUA meta-analysis. The PM-Plot (right panel) is separating studies predicted to have an effect on PTSD (red) from underpowered studies with ambiguous effects (green) or predicted to have no effect (blue). For a detailed study description see Supplementary Methods and Supplementary Data 1.

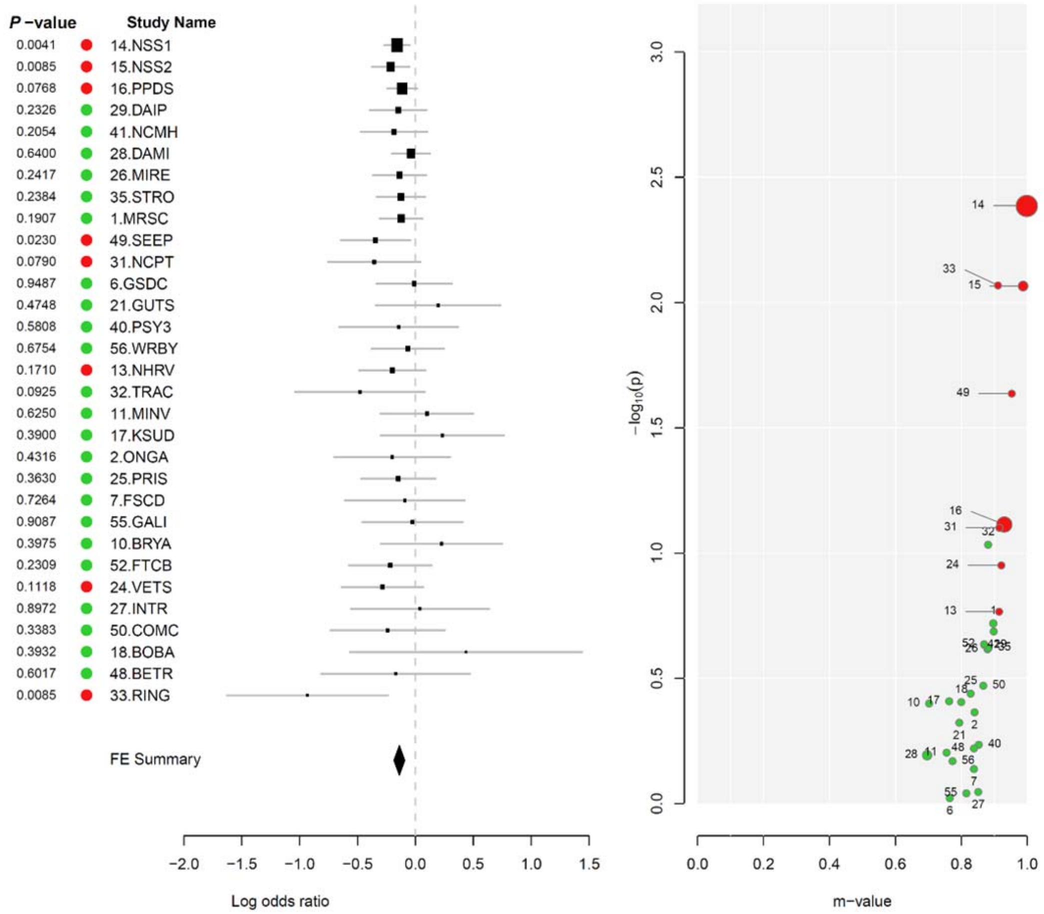

Supplementary Figure 12: Forest plot (left panel) of the male European ancestry top hit rs571848662 (*P* = 7.88 x 10^{-9}) showing effect sizes for each of the 31 included GWAS in the male EA meta-analysis. The PM-Plot (right panel) is separating studies predicted to have an effect on PTSD (red) from underpowered studies with ambiguous effects (green) or predicted to have no effect (blue). For a detailed study description see Supplementary Methods and Supplementary Data 1.

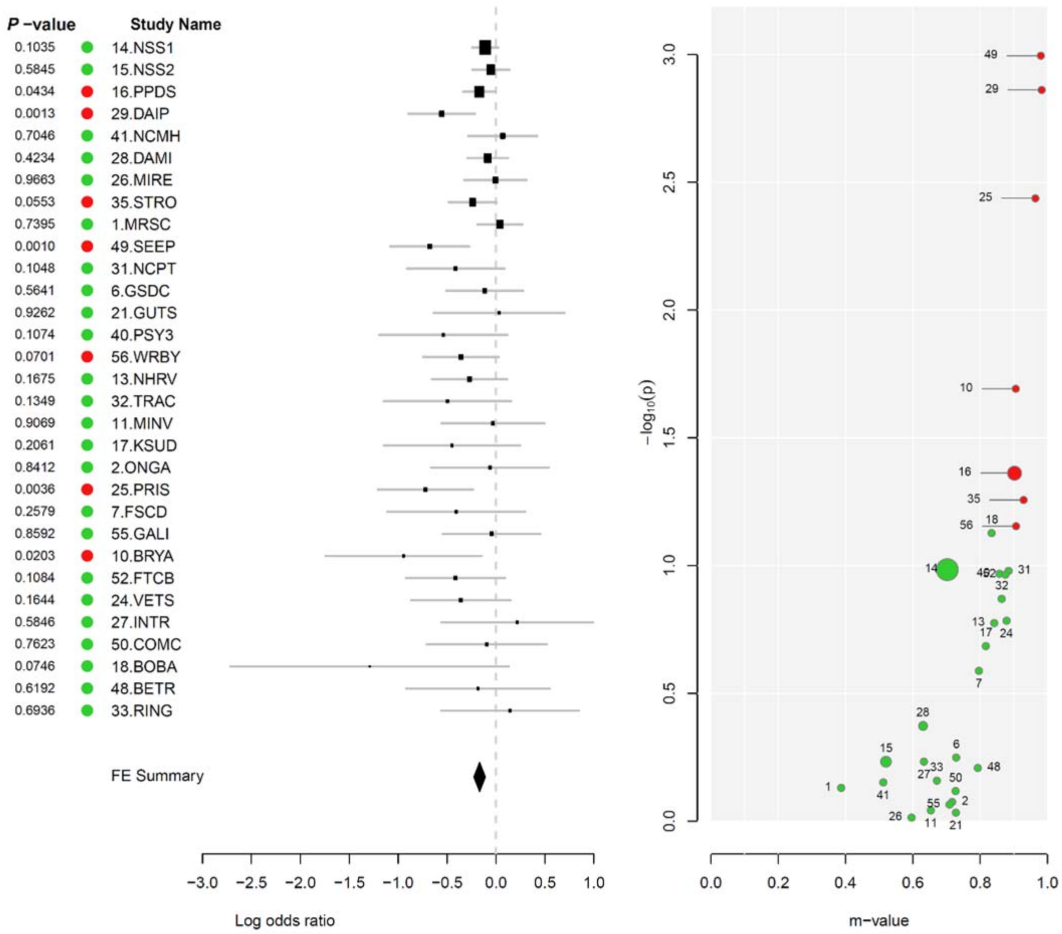

Supplementary Figure 13: Forest plot (left panel) of the male European ancestry second hit rs148757321 (*P* = 3.76 x 10^{-8}) showing effect sizes for each of the 31 included GWAS in the male EUA meta-analysis. The PM-Plot (right panel) is separating studies predicted to have an effect on PTSD (red) from underpowered studies with ambiguous effects (green) or predicted to have no effect (blue). For a detailed study description see Supplementary Methods and Supplementary Data 1.

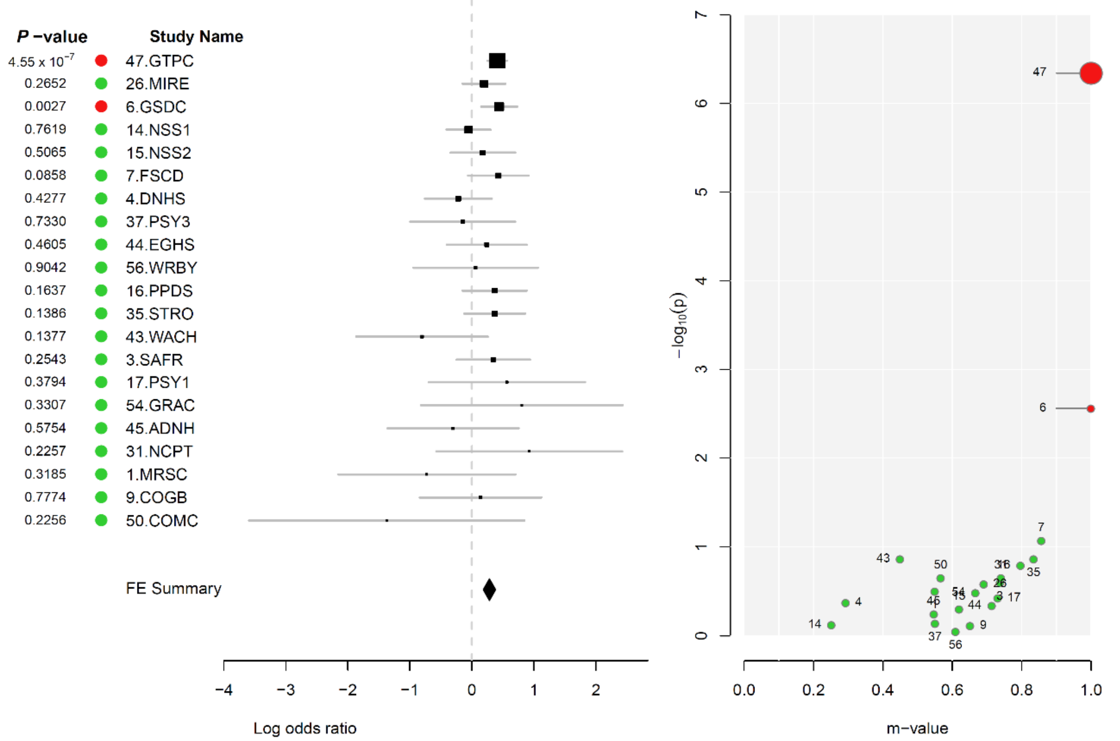

Supplementary Figure 14: Forest plot (left panel) of the African ancestry top hit rs115539978 (P = 2.79 x 10^{-8}) showing effect sizes for each of the 21 included GWAS in the AFA meta-analysis. The PM-Plot (right panel) is separating studies predicted to have an effect on PTSD (red) from underpowered studies with ambiguous effects (green) or predicted to have no effect (blue). For a detailed study description see Supplementary Methods and Supplementary Data 1.

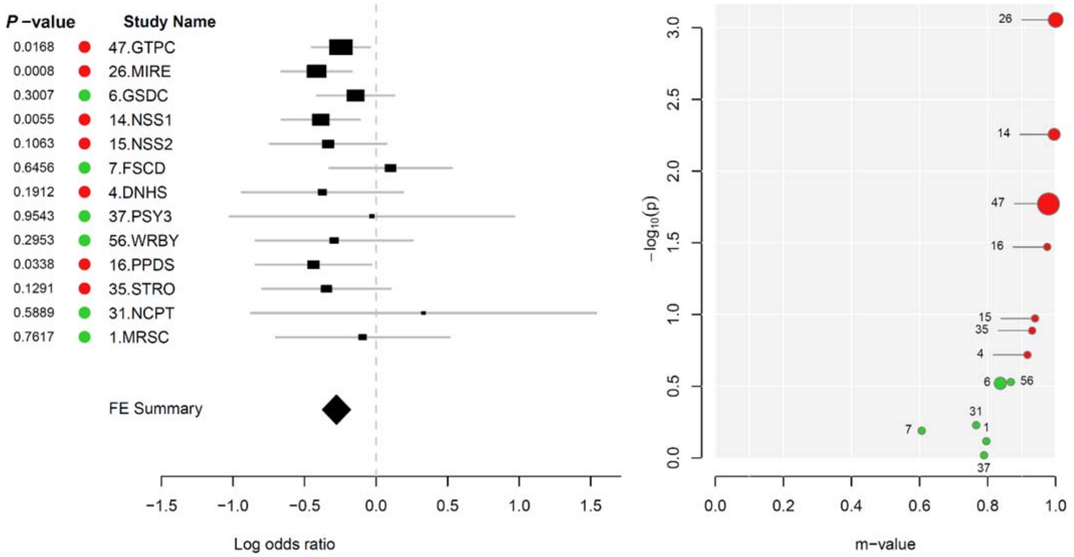

Supplementary Figure 15: Forest plot (left panel) of the male African ancestry top hit rs142174523 (*P* = 4.31 x 10^{-8}) showing effect sizes for each of the 13 included GWAS in the male AFA meta-analysis. The PM-Plot (right panel) is separating studies predicted to have an effect on PTSD (red) from underpowered studies with ambiguous effects (green) or predicted to have no effect (blue). For a detailed study description see Supplementary Methods and Supplementary Data 1.

Ancestry	A1	A2	A1 Freq	OR	SE	P-value	Direction	Neff
EUA	a	t	0.34	1.12	0.02	3.16E-09	++--++?+++++--+++++++-?-++++--++++++?--+++-	30,274
AFA	a	t	0.55	0.99	0.03	0.66	--++-?+-++--+---+--++	10,212
AMA	a	t	0.30	0.92	0.05	0.09	----+-	4,499

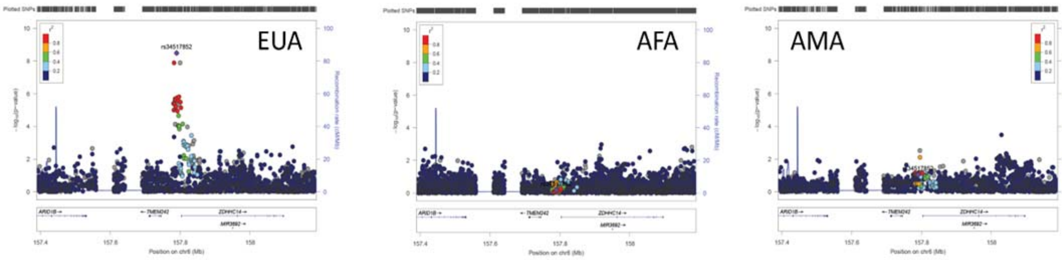

Supplementary Figure 16: Comparison of the European ancestry top hit rs34517852 across the European (EUA), African (AFA), and Latino (AMA) PTSD GWAS. Chromosomal position of the regional association plots is indicated on the x-axis, -log10 *P*-values for each SNP (filled circles) is indicated on the y-axis, with the lead SNP shown in purple. Annotated genes in the region are drawn in the lower panel. Recombination rate is indicated by a blue line. Additional SNPs in the locus are colored according to linkage disequilibrium (r^2) with the lead SNP. *A1* allele 1 (coded allele), A2 allele 2, *A1 Freq* A1 allele frequency, *OR* odds ratio, *SE* standard error, *Neff* effective number of subjects

Ancestry	A1	A2	A1 Freq	OR	SE	P-value	Direction	Neff
EUA	t	c	0.13	0.88	0.02	4.36E-08	--++---+-+------------+-+----+-+--+-------+	70,332
AFA	t	c	0.11	1.04	0.04	0.32	+++++--+-+-+-+-++---+	11,322
AMA	t	c	0.07	0.97	0.10	0.72	+--++-	4,499

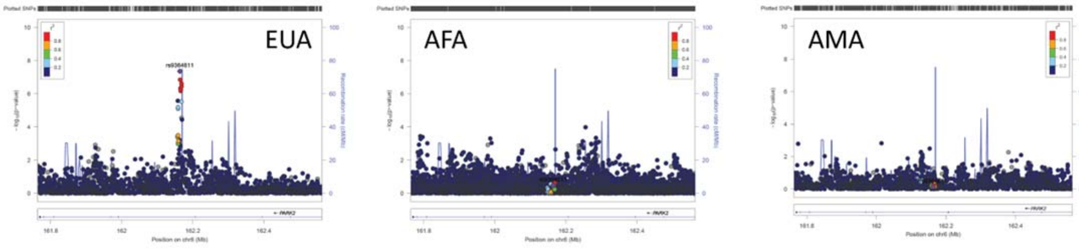

Supplementary Figure 17: Comparison of the European ancestry second hit rs9364611 across the European (EUA), African (AFA), and Latino (AMA) PTSD studies. Chromosomal position of the regional association plots is indicated on the x-axis, -log10 *P*-values for each SNP (filled circles) is indicated on the y-axis, with the lead SNP shown in purple. Annotated genes in the region are drawn in the lower panel. Recombination rate is indicated by a blue line. Additional SNPs in the locus are colored according to linkage disequilibrium (r^2) with the lead SNP. *A1* allele 1 (coded allele), A2 allele 2, *A1 Freq* A1 allele frequency, *OR* odds ratio, *SE* standard error, *Neff* effective number of subjects

Ancestry	A1	A2	A1 Freq	OR	SE	P-value	Direction	Neff
EUA	t	tatac	0.61	0.87	0.02	7.88E-09	----++----++---+--?-----------?+-	16,964
AFA	t	tatac	0.69	1.03	0.05	0.61	-++-++-+-+-++	4,702
AMA	t	tatac	0.59	1.01	0.09	0.89	+-+-+	1,259

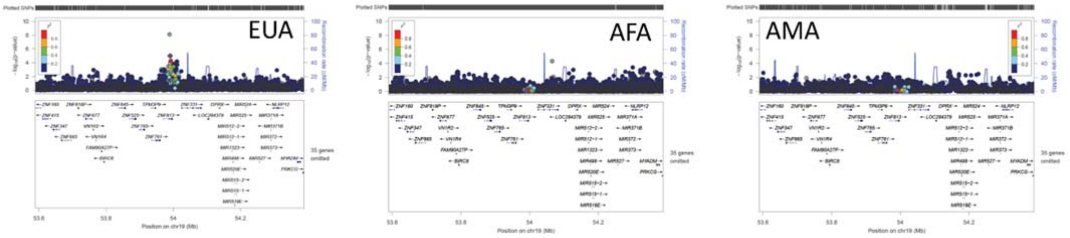

Supplementary Figure 18: Comparison of the European ancestry male top hit rs571848662 across the European (EUA), African (AFA), and Latino (AMA) PTSD studies. Chromosomal position of the regional association plots is indicated on the x-axis, -log10 *P*-values for each SNP (filled circles) is indicated on the y-axis, with the lead SNP shown in purple. Annotated genes in the region are drawn in the lower panel. Recombination rate is indicated by a blue line. Additional SNPs in the locus are colored according to linkage disequilibrium (r^2) with the lead SNP. *A1* allele 1 (coded allele), A2 allele 2, *A1 Freq* A1 allele frequency, *OR* odds ratio, *SE* standard error, *Neff* effective number of subjects

Ancestry	A1	A2	A1 Freq	OR	SE	P-value	Direction	Neff
EUA	ctgtg	c	0.83	1.18	0.03	3.76E-08	-++++++++++-+++-++?+++++-+-+++?++	16,964
AFA	ctgtg	c	0.90	0.94	0.07	0.38	-++----+-++-+	4,702
AMA	ctgtg	c	0.72	0.95	0.09	0.60	--++-	1,259

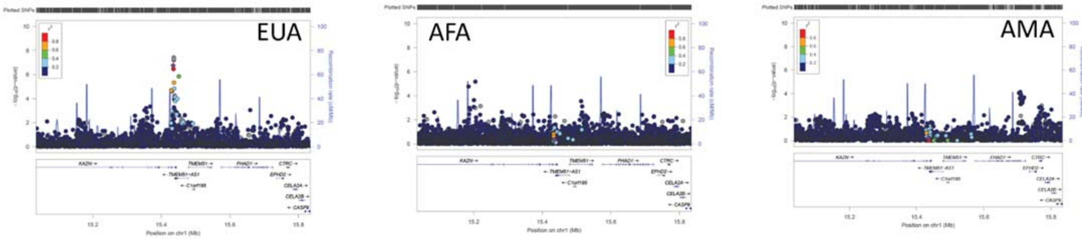

Supplementary Figure 19: Comparison of the European ancestry male second hit rs148757321 across the European (EUA), African (AFA), and Latino (AMA) PTSD studies. Chromosomal position of the regional association plots is indicated on the x-axis, -log10 *P*-values for each SNP (filled circles) is indicated on the y-axis, with the lead SNP shown in purple. Annotated genes in the region are drawn in the lower panel. Recombination rate is indicated by a blue line. Additional SNPs in the locus are colored according to linkage disequilibrium (r^2) with the lead SNP. *A1* allele 1 (coded allele), A2 allele 2, *A1 Freq* A1 allele frequency, *OR* odds ratio, *SE* standard error, *Neff* effective number of subjects

Ancestry	A1	A2	A1 Freq	OR	SE	P-value	Direction	Neff
AFA	t	c	0.07	1.33	0.05	2.79E-08	--++++++-+++++--+-+-+	11,322
EUA	NA	NA	NA	NA	NA	NA	NA	NA
AMA	NA	NA	NA	NA	NA	NA	NA	NA

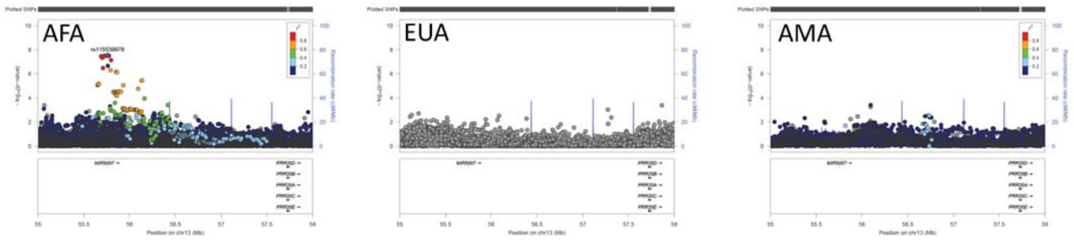

Supplementary Figure 20. Comparison of the African ancestry top hit rs115539978 across the African (AFA), European (EUA), and Latino (AMA) PTSD studies. Chromosomal position of the regional association plots is indicated on the x-axis, -log10 *P*-values for each SNP (filled circles) is indicated on the y-axis, with the lead SNP shown in purple. Annotated genes in the region are drawn in the lower panel. Recombination rate is indicated by a blue line. Additional SNPs in the locus are colored according to linkage disequilibrium (r^2) with the lead SNP. *A1* allele 1 (coded allele), A2 allele 2, *A1 Freq* A1 allele frequency, *OR* odds ratio, *SE* standard error, *Neff* effective number of subjects

Ancestry	A1	A2	A1 Freq	OR	SE	P-value	Direction	Neff
AFA	a	g	0.30	0.76	0.05	4.31E-08	---+-----+---	4,702
EUA	a	g	0.27	1.01	0.03	0.78	+----++++++-------?+?++-+++---?++	16,821
AMA	a	g	0.21	1.00	0.11	0.97	+---+	1,259

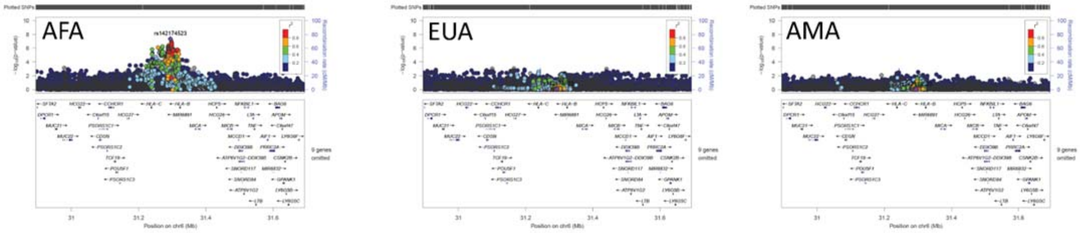

Supplementary Figure 21. Comparison of the African ancestry male top hit rs142174523 across the African (AFA), European (EUA), and Latino (AMA) PTSD studies. Chromosomal position of the regional association plots is indicated on the x-axis, -log10 *P*-values for each SNP (filled circles) is indicated on the y-axis, with the lead SNP shown in purple. Annotated genes in the region are drawn in the lower panel. Recombination rate is indicated by a blue line. Additional SNPs in the locus are colored according to linkage disequilibrium (r^2) with the lead SNP. *A1* allele 1 (coded allele), A2 allele 2, *A1 Freq* A1 allele frequency, *OR* odds ratio, *SE* standard error, *Neff* effective number of subjects

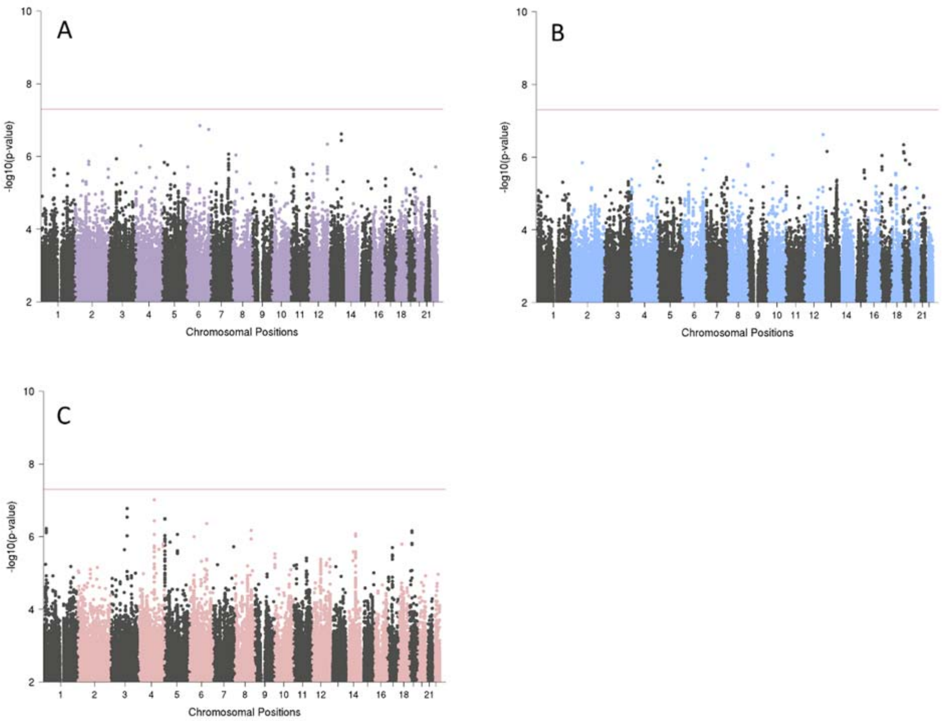

Supplementary Figure 22. Manhattan plots from trans-ancestry meta-analyses of GWAS for PTSD including subjects of European, African, and Latino ancestries. Results are shown for GWAS in all subject subjects (panel A), and for sex-stratified analyses in men (panel B) and women (panel C), respectively. The red line represents genome-wide significance at $P < 5 \times 10^{-8}$.

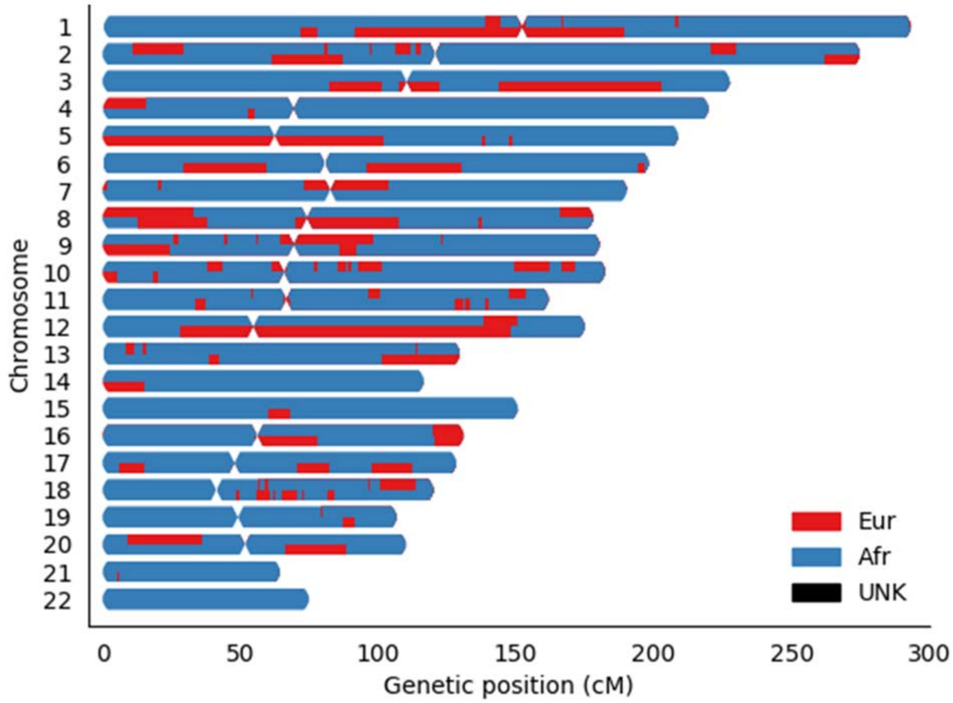

Supplementary Figure 23. Example of a painted karyogram showing ancestral population tracts across the autosomes of an African American individuals. *EUR* European tract, *Afr* African tract, *UNK* tract of unknown origin

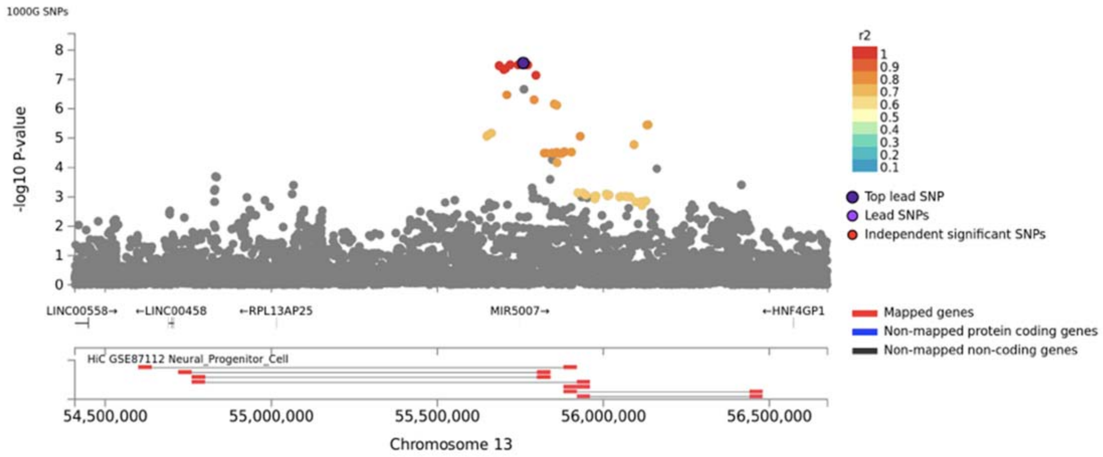

Supplementary Figure 24. Hi-C data providing evidence for chromatin interaction of the African-ancestry GWAS meta-analysis top hit rs115539978. SNP rs115539978 is on chromosome 13 with a region about 1,100kb upstream harboring additional non-coding RNAs including LINC00458, hsa-mir-1297 and LINC00558 as well as a region approximately 820kb downstream harboring the pseudogene *HNF4GP1*. Chromosomal position of the regional association plots is indicated on the x-axis, -log10 *P*-values for each SNP (filled circles) is indicated on the y-axis, with the lead SNP shown in purple. Additional SNPs in the locus are colored according to linkage disequilibrium (r^2) with the lead SNP.

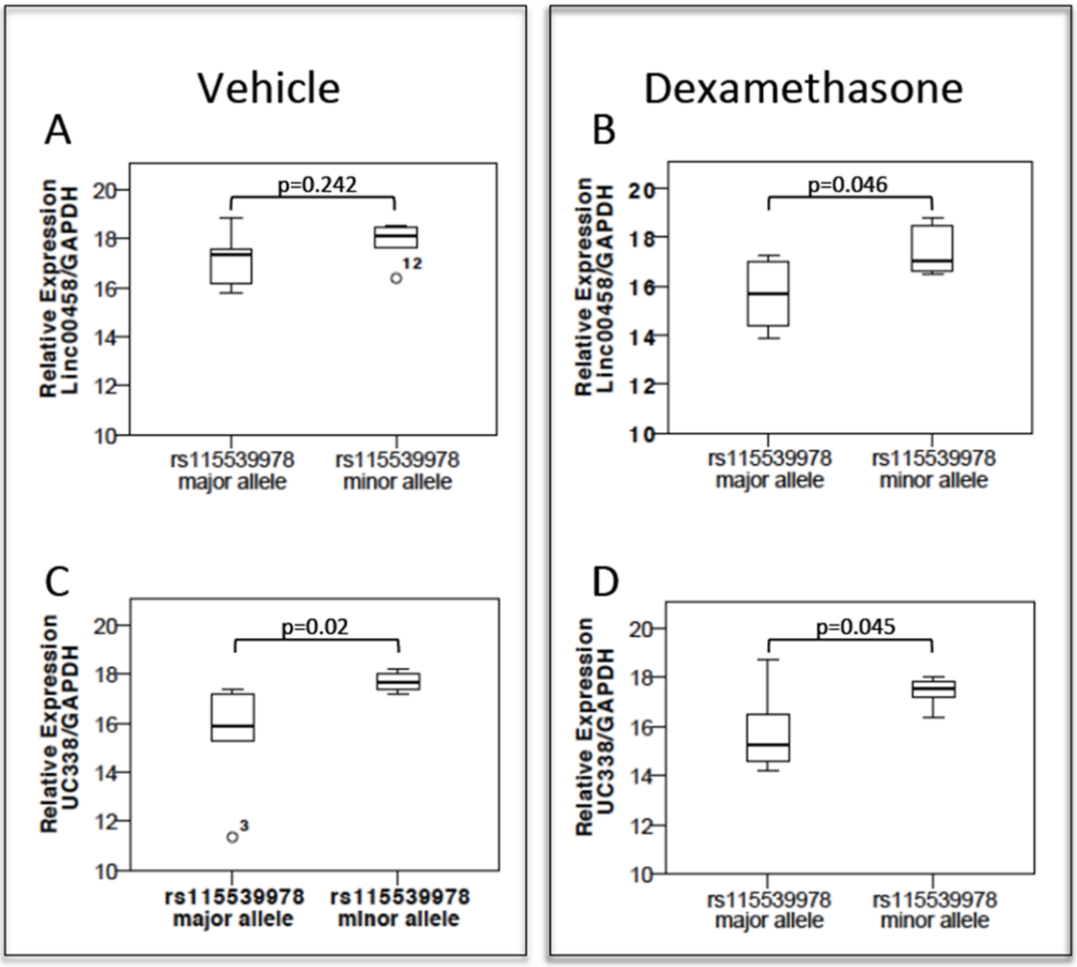

Supplementary Figure 25. Dexamethasone-induced differential expression of Linc00458 and UC338 is rs115539978 genotype dependent. Vehicle control experiments for Linc00458 (panel A) and TUC338 (panel C), and Dex stimulation for Linc00458 (panel B) and TUC338 (panel D) are shown.

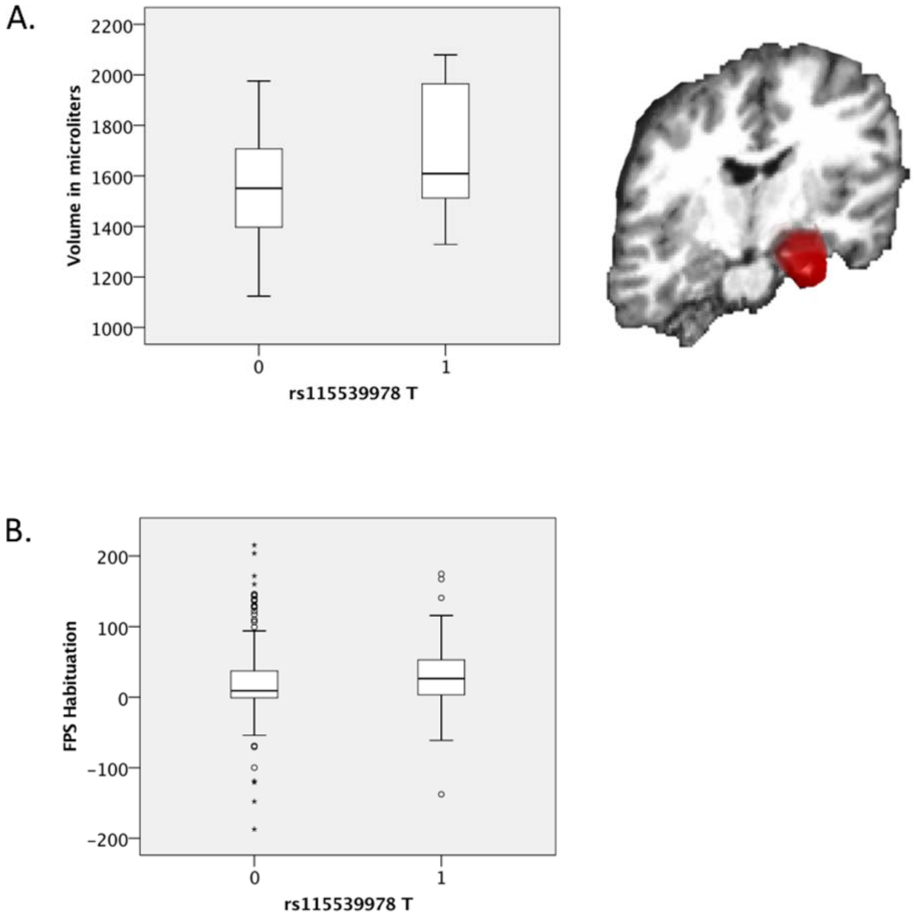

Supplementary Figure 26. Neuroimaging and physiology related to the African ancestry top hit rs115539978. Neuroimaging findings (panel A) and physiological findings (panel B) comparing carriers of the minor T-allele versus homozygous subjects for the C-allele in African-American subjects from the Grady Trauma project (study #47, GTPC) showing significant differences in left amygdala volume (*N* = 73 CC, and 14 T-allele carriers, *P* = 0.03) and with fear potentiated startle habituation (*N* = 248 CC, and 51 T carriers, *P* = 0.048). SNP effects were identified after controlling for ancestry components PCs 1-5, age, and intracranial volume (ICV). There was no effect of SNP on ICV, *P* = 0.76. Box and whisker plots show median and interquartile range in each group. Brain image shows a 3D rendering of the left amygdala segmentation for a representative participant, overlaid on a coronal slice of that participant's T1-w image in radiological orientation (right is left).

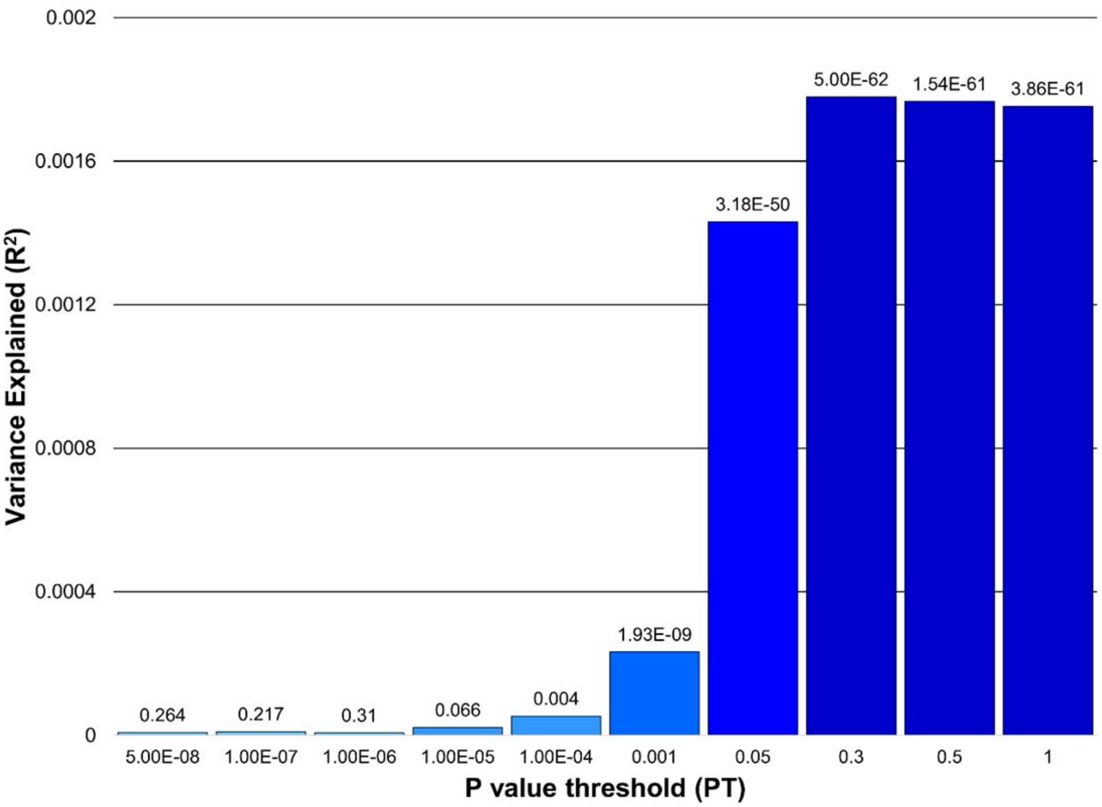

Supplementary Figure 27. Polygenic risk score (PRS) plot from PRSice showing results at broad *P*-value thresholds for PGC2 PTSD GWAS predicting PTSD re-experiencing symptoms in Million Veteran Program (MVP).[198] A bar for the best-fit PRS ($P = 5.2 \times 10^{-62}$) from the high-resolution run at a p-value threshold of 0.3 is also included (darkest blue). A total of 100 *P*-value thresholds were tested (adjusted $P = 5.2 \times 10^{-60}$).

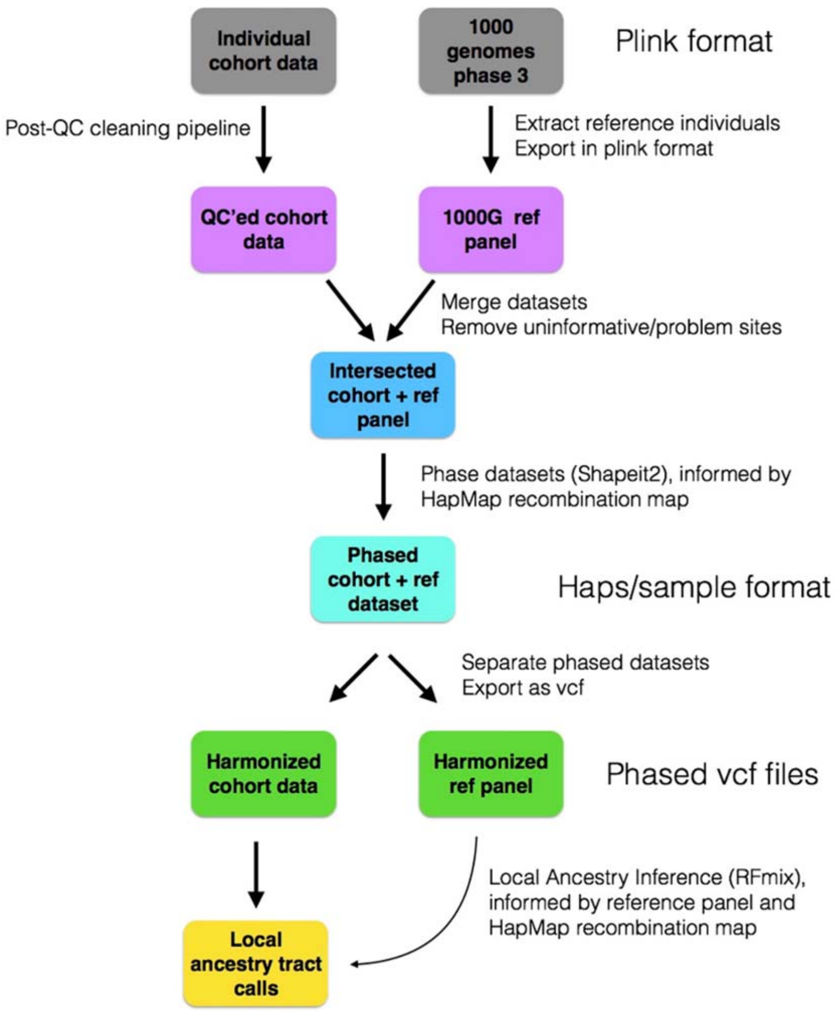

Supplementary Figure 28: Schematic of the local ancestry pipeline developed to infer local ancestry (LAI) in admixed subjects.

Supplementary Tables

Supplementary Table 1 Heritability sensitivity analyses in UK Biobank based on LD-score regression (LDSC)

						10% prevalence			30% prevalence			50% prevalence				
**Group**	**Sample**	***N* SNPs**	***N* Cases**	***N* Controls**	***N* Total**	h^2_{SNP}	**SE**	**95% CI**	h^2_{SNP}	**SE**	**95% CI**	h^2_{SNP}	**SE**	**95% CI**	**z**	***p*-value**
All	UKBB_p1	1,175,791	10,389	115,799	126,188	0.13	0.01	0.1 - 0.15	0.17	0.02	(0.1 - 0.15)	0.19	0.02	0.15 - 0.23	8.75	2.1×10^{-18}
	UKBB_p2	1,280,135	7,047	56,988	64,035	0.13	0.02	0.08 - 0.17	0.17	0.03	(0.08 - 0.17)	0.19	0.03	0.12 - 0.25	5.76	8.5×10^{-9}
	UKBB_p3	1,280,135	7,047	26,935	33,982	0.19	0.02	0.14 - 0.23	0.26	0.03	(0.14 - 0.23)	0.28	0.04	0.21 - 0.35	7.79	6.4×10^{-15}
Men	UKBB_p1	1,175,791	3,544	51,700	55,244	0.11	0.04	0.04 - 0.17	0.15	0.05	(0.04 - 0.17)	0.16	0.05	0.05 - 0.26	2.99	1.4×10^{-3}
	UKBB_p2	1,280,135	2,658	28,443	31,101	0.10	0.06	-0.02 - 0.22	0.14	0.09	(-0.02 - 0.22)	0.15	0.09	-0.04 - 0.33	1.58	0.114
	UKBB_p3	1,280,135	2,658	15,238	17,896	0.14	0.05	0.04 - 0.24	0.20	0.07	(0.04 - 0.24)	0.21	0.08	0.06 - 0.37	2.75	6.0×10^{-3}
Women	UKBB_p1	1,160,174	6,845	64,099	70,944	0.14	0.02	0.1 - 0.18	0.19	0.03	(0.1 - 0.18)	0.21	0.03	0.14 - 0.27	6.36	2.0×10^{-10}
	UKBB_p2	1,280,135	4,389	28,545	32,934	0.08	0.03	0.02 - 0.14	0.11	0.04	(0.02 - 0.14)	0.12	0.05	0.03 - 0.21	2.66	7.8×10^{-3}
	UKBB_p3	1,280,135	4,389	11,697	16,086	0.17	0.04	0.1 - 0.24	0.23	0.05	(0.1 - 0.24)	0.25	0.05	0.14 - 0.36	4.61	4.0×10^{-6}

PTSD screening in UKBB was based on self-reported symptoms from a mental health survey.
Estimates are calculated at different population prevalences after trauma exposure, for alternative subject selection criteria, and separately for men and women.
P-value is testing if h^2_{SNP} is different from zero and applies to all prevalences, *UKBB* UK Biobank European subjects, *UKBB_p1* all cases and controls (cases: PCL>=13; controls: PCL<13), *UKBB_p2* only trauma-exposed cases and controls (cases: PCL>=13; controls: PCL<13) *UKBB_p3* only trauma-exposed cases and controls (cases: PCL>=13; controls: PCL=4), h^2_{snp} mean SNP-based heritability, *SE* standard error, *CI* confidence interval; *z* test-statistic

Supplementary Table 2. Comparison of genetic correlations (rg) between different subsets of PGC2 studies based on LD-score regression.

			Subset 1				Subset 2							
**Subset 1**	**Subset 2**	***N* SNPs**	***N* cases**	***N* controls**	h^2_{SNP}	**SE**	***N* cases**	***N* controls**	h^2_{SNP}	**SE**	r_g	**SE**	**z**	***p*-value**
PGC1.5 smal	PGC1.5 large	1,154,022	2,102	7,366	0.12	0.06	10,721	28,282	0.03	0.00	0.45	0.26	1.71	0.087
UKB w	UKB m	1,160,174	6,845	64,099	0.19	0.03	3,544	51,700	0.15	0.05	1.03	0.24	4.31	1.6×10^{-5}
PGC1.5 w	PGC1.5 m	1,162,530	6,128	9,528	0.21	0.05	6,364	23,905	0.01*	0.03	N/A	N/A	N/A	N/A
PGC1.5 w	UKB	1,157,449	6,128	9,528	0.21	0.05	10,389	115,799	0.18	0.02	0.46	0.13	3.56	4.0×10^{-4}
PGC1.5 w	UKB w	1,164,765	6,128	9,528	0.21	0.05	6,845	64,099	0.19	0.03	0.46	0.14	3.34	8.0×10^{-4}
PGC1.5 w	UKB m	1,158,070	6,128	9,528	0.21	0.05	3,544	51,700	0.15	0.05	0.44	0.23	1.94	0.052
PGC1.5 m	UKB m	1,159,417	6,364	23,905	0.01*	0.03	3,544	51,700	0.15	0.05	N/A	N/A	N/A	0.70
PGC1.5 m	UKB w	1,158,070	6,364	23,905	0.01*	0.03	6,845	64,099	0.19	0.03	N/A	N/A	N/A	N/A
PGC1.5	UKB	1,175,791	12,823	35,648	0.05	0.02	10,389	115,799	0.18	0.02	0.73	0.21	3.50	0.0005

PGC1.5 PGC2 studies not including the UK Biobank; here only subjects of European ancestry are included, *UKB* UK Biobank subjects of European ancestry
r_g genetic correlation, *SE* standard error, *z* test-statistic, *N/A* fails to run, * $p > 0.05$

Supplementary Table 3 Description of European ancestry (EUA) meta-analysis of 43 GWAS including 174,659 subjects

		N subjects		Males		Females		*N* total
Study No.	abbrev.	cases	controls	cases	controls	cases	controls	
1	MRSC	310	2,199	304	2,197	N/A	N/A	2,509
2	ONGA	118	102	88	82	N/A	N/A	220
5	NHS2	579	741	N/A	N/A	579	741	1,320
6	GSDC	263	1,227	100	785	163	442	1,490
7	FSCD	111	436	45	230	66	206	547
9	COGA	49	894	N/A	N/A	N/A	N/A	943
9	COGB	49	815	N/A	N/A	N/A	N/A	864
10	BRYA	87	572	41	350	46	222	659
13	NHRV	156	2,136	119	1,967	37	169	2,292
14	NSS1	1,264	3,586	1,028	3,148	236	438	4,850
15	NSS2	909	949	752	784	157	165	1,858
16	PPDS	677	4,295	637	4,116	39	164	4,972
17	KSUD	121	96	57	72	47	20	217
18	BOBA	70	68	25	24	45	44	138
21	GUTS	261	255	70	67	191	188	516
22	NHSY	2,652	2,611	N/A	N/A	2,652	2,611	5,263
24	VETS*	85	1,114	85	1,114	N/A	N/A	1,199
25	PRIS	113	706	100	642	N/A	N/A	819
26	MIRE	418	405	343	343	75	62	823
27	INTR	78	116	69	55	N/A	N/A	194
28	DAMI	462	2,019	407	1,919	55	100	2,481
29	DAIP	666	2,721	208	847	458	1,874	3,387
30	QIMR*	325	1,797	101	934	224	863	2,122
31	NCPT	299	162	215	84	84	78	461
32	TRAC	148	49	131	49	N/A	N/A	197
33	RING	35	156	35	151	N/A	N/A	191
35	STRO	320	1,926	298	1,829	N/A	N/A	2,246
36	BRY2	68	56	N/A	N/A	40	30	124
41	NCMH	622	622	234	235	388	387	1,244
42	EACR	67	231	N/A	N/A	41	168	298
43	WACH	56	56	N/A	N/A	56	56	112
47	GTPC	80	107	N/A	N/A	61	58	187
48	BETR	46	53	45	53	0	0	99
49	SEEP	306	325	204	223	102	102	631
50	COM1	75	134	70	122	0	0	209
52	FTCB	85	953	80	911	0	0	1,038
54	GRAC	76	92	N/A	N/A	76	92	168
55	GALI	100	123	100	123	N/A	N/A	223
60	UKB**	10,389	115,799	3,544	51,700	6,845	64,099	126,188
11_12	MINV	145	96	142	88	N/A	N/A	241
19-20	PSY1	62	86	N/A	N/A	45	51	148
37-40	PSY3	232	306	64	109	165	197	538
56-59	WRBY	178	255	167	252	N/A	N/A	433
Meta-analysis		23,212	151,447	9,908	75,605	12,973	73,627	174,659

N/A not enough subjects for analysis
*linear mixed models in GEMMA to account for relatedness
**linear regression in BGenie v1.2

Supplementary Table 4 Comparison between full and sex-stratified analyses for genome-wide significant markers

Variant	Studies	A1	A2	Sex	A1 freq	Beta	SE	OR	*P*-value	*N* cases	*N* controls	*N* effective	*p*-sex difference*
rs34517852	EUA both	a	t	both	0.341	0.110	0.019	1.12	3.2×10^{-9}	12,080	33,446	30,274	
				male	0.344	0.113	0.025	1.12	7.5×10^{-6}	5,920	22,628	16,278	0.80
				female	0.339	0.104	0.029	1.11	2.8×10^{-4}	5,829	8,603	12,906	
rs9364611	EUA both	t	c	both	0.131	-0.124	0.023	0.88	4.4×10^{-8}	23,212	151,447	70,332	
				male	0.132	-0.109	0.032	0.90	6.3×10^{-4}	9,908	75,605	30,595	0.51
				female	0.131	-0.139	0.034	0.87	3.3×10^{-5}	12,973	73,627	38,491	
rs115539978	AFA both	t	c	both	0.074	0.284	0.051	1.33	2.8×10^{-8}	4,363	10,976	11,322	
				male	0.073	0.279	0.079	1.32	4.3×10^{-4}	1,782	5,361	4,702	0.68
				female	0.075	0.323	0.070	1.38	4.0×10^{-6}	2,360	4,926	6,064	
rs571848662	EUA m	t	tatac	both	0.608	-0.083	0.018	0.92	2.8×10^{-6}	12,498	33,851	31,097	
				male	0.608	-0.139	0.024	0.87	7.9×10^{-9}	6,263	22,971	16,964	8.4×10^{-4}
				female	0.607	-0.017	0.027	0.98	0.530	5,904	8,665	13,042	
rs148757321	EUA m	ctgtg	c	both	0.827	0.091	0.022	1.10	3.2×10^{-5}	12,498	33,851	31,097	
				male	0.828	0.168	0.031	1.18	3.8×10^{-8}	6,263	22,971	16,964	9.6×10^{-4}
				female	0.826	0.018	0.034	1.02	0.590	5,904	8,665	13,042	
rs142174523	AFA m	a	g	both	0.305	-0.122	0.031	0.89	1.0×10^{-4}	4,363	10,976	11,322	
				male	0.300	-0.277	0.051	0.76	4.3×10^{-8}	1,782	5,361	4,702	9.9×10^{-5}
				female	0.307	-0.021	0.042	0.98	0.620	2,360	4,926	6,064	

A1 allele 1 (coded allele), *A2* allele2, *A1 freq* A1 allele frequency
* z-test on differences between effect sizes (Beta) of male and female meta-analyses

Supplementary Table 5 Description of African ancestry (AFA) meta-analysis of 21 GWAS including 15,339 subjects

		N subjects		Males		Females		*N* total
Study No.	abbrev.	cases	controls	cases	controls	cases	controls	
1	MRSC	39	193	39	193	N/A	N/A	232
3	SAFR	77	139	N/A	N/A	77	139	216
4	DNHS	155	481	52	208	103	273	636
6	GSDC	350	2,140	168	1,201	182	939	2,490
7	FSCD	159	441	71	230	88	211	600
9	COGB	37	359	N/A	N/A	N/A	N/A	396
14	NSS1	328	1,107	210	825	118	282	1,435
15	NSS2	202	223	129	154	73	69	425
16	PPDS	107	852	92	750	N/A	N/A	959
26	MIRE	566	544	378	381	188	163	1,110
31	NCPT	40	32	30	23	N/A	N/A	72
35	STRO	98	518	80	425	N/A	N/A	616
43	WACH	78	72	N/A	N/A	78	72	150
45	ADNH	50	47	N/A	N/A	N/A	N/A	97
47	GTPC	1,546	3,412	371	902	1,175	2,510	4,958
50	COM1	30	40	N/A	N/A	N/A	N/A	70
54	GRAC	52	33	N/A	N/A	52	33	85
17-20	PSY1	63	57	N/A	N/A	32	30	120
37-40	PSY3	140	66	64	16	73	50	206
56-59	WRBY	117	58	98	53	N/A	N/A	175
44	EGHS	129	162	N/A	N/A	121	155	291
Meta-analysis		4,363	10,976	1,782	5,361	2,360	4,926	15,339

N/A not enough subjects for analysis

Supplementary Table 6 Description of American ancestry (AMA) meta-analysis of 6 GWAS including 5,703 subjects

		N subjects		Males		Females		*N* total
Study No.	abbrev.	cases	controls	cases	controls	cases	controls	
1	MRSC	83	535	81	533	N/A	N/A	618
14	NSS1	128	499	97	422	N/A	N/A	627
15	NSS2	106	127	83	101	23	26	233
16	PPDS	85	677	72	634	N/A	N/A	762
23	PROM	1,508	1,526	N/A	N/A	1,508	1,526	3,034
35	STRO	71	358	67	322	N/A	N/A	429
Meta-analysis		1,981	3,722	400	2,012	1,531	1,552	5,703

N/A not enough subjects for analysis

Supplementary Table 7 Local ancestry analyses for the African ancestry GWAS hit rs115539978

Description of allele frequencies stratified by copies and type of ancestral haplotypes

Ancestal background	A1	A2	A1 freq	*N* * case	*N* * Control	*N* * total
1 European	t	c	0.008	953	2,788	3,741
2 European	t	c	0.003	152	447	599
1 African	t	c	0.080	1,025	2,945	3,970
2 African	t	c	0.089	2,556	6,960	9,516
1 Native American	t	c	0.008	100	253	353
2 Native American	t	c	0	0	2	2

Comparison of SNP association with PTSD including 5 global PC's versus local ancestry inference (LAI) in the analysis

Model	A1	A2	A1 freq	OR	SE	*P*-value	*N* cases	*N* controls	*N* total
SNP effect (with 5 global PCs)	t	c	0.072	1.31	0.052	2.4×10^{-7}	3,747	10,402	14,149
SNP effect (with LAI + 5 global P	t	c	0.072	1.30	0.053	7.1×10^{-7}	3,747	10,402	14,149

PTSD predicted by copies of A1 allele on specific ancestral background

Ancestral background	A1	A2	OR	SE	*P*-value
European	t	c	0.70	0.462	0.44
African	t	c	1.32	0.053	9.6×10^{-8}
Native American	t	c	0.00	112.271	0.93

*subjects with haplotypes of different ancestry are counted twice

Supplementary Table 8 Local ancestry analyses for the African ancestry male GWAS hit rs142174523

Description of allele frequencies stratified by copies and type of ancestral haplotypes

**Ancestal background**	**A1**	**A2**	**A1 freq**	***N*** *** case**	***N*** *** Control**	***N*** *** total**
1 European	a	g	0.25	419	1,358	1,777
2 European	a	g	0.23	70	206	276
1 African	a	g	0.34	453	1,526	1,979
2 African	a	g	0.32	821	3,150	3,971
1 Native American	a	g	0.40	80	312	392
2 Native American	a	g	0.29	6	8	14

Comparison of SNP association with PTSD including 5 global PC's versus local ancestry inference (LAI) in the analysis

**Model**	**A1**	**A2**	**A1 freq**	**OR**	**SE**	***P*** **-value**	***N*** **cases**	***N*** **controls**	***N*** **total**
SNP effect (with 5 global PCs)	a	g	0.31	0.80	0.051	8.0×10^{-6}	1,373	4,962	6,335
SNP effect (with LAI + 5 global P	a	g	0.31	0.80	0.051	1.4×10^{-5}	1,373	4,962	6,335

PTSD predicted by copies of A1 allele on specific ancestral background

**Ancestral background**	**A1**	**A2**	**OR**	**SE**	***P*** **-value**
European	a	g	0.80	0.115	0.055
African	a	g	0.79	0.055	2.2×10^{-5}
Native American	a	g	0.89	0.215	0.57

*subjects with haplotypes of different ancestry are counted twice

Supplementary Table 9 Replication of genome-wide significant findings in the Million Veterans Program (MVP)

Subjects (original finding)	Replication (MVP)*	Variant	Replication Variant (LD)**	Chr	Position (bp)	A1	A2	A1 freq	Beta	SE	*P*-value	*N* subjects†
European ancestry												
all	all	rs34517852	rs34517852	6	157789333	a	t	0.342	0.0002	0.018	0.990	146,660
all	all	rs9364611	rs9364611	6	162163506	t	c	0.130	-0.027	0.025	0.264	146,660
male	all (6.6% female)	rs571848662	rs8112292 (0.75)	19	53988841	t	c	0.468	0.005	0.017	0.782	146,660
male	all (6.6% female)	rs148757321	rs148757321	1	15436223	ctgtg	c	0.829	0.024	0.022	0.274	146,660
African ancestry												
all	all	rs115539978	rs115539978	13	55759209	t	c	0.071	-0.119	0.112	0.289	19,983
male	all	rs142174523	rs9265461 (0.77)	6	31294290	a	g	0.361	0.011	0.064	0.857	19,983

The MVP GWAS is on re-experiencing symptoms, a core feature of PTSD

A1 allele 1 (coded allele), *A2* allele2, *A1 freq* A1 allele frequency, *LD* Linkage Disequilibrium estimate with variant
* Replication (MVP): no sex-stratified analyses available; cohort is predominantly male
** If variant was not genotyped in MVP, an LD proxy variant was used instead. LD between variant and proxy is noted in parenthesis
† *N subjects* number of MVP subjects with re-experiencing symptoms phenotype (REX)

Supplementary Table 10 Meta-analysis of European ancestry GWAS, before and after conditioning on MDD

Subjects	Variant	Chr	Position (bp)	A1	A2	A1 freq	Beta	SE	OR	*P*-value	Beta_cojo	SE_cojo	OR_cojo	*P*_cojo	*N* cases	*N* controls
EUA all	rs34517852	6	157789333	a	t	0.341	0.110	0.019	1.12	3.2×10^{-9}	0.112	0.019	1.12	1.8×10^{-9}	12,080	33,446
EUA all	rs9364611	6	162163506	t	c	0.131	-0.124	0.023	0.88	4.4×10^{-8}	-0.128	0.023	0.88	1.7×10^{-8}	23,212	151,447
EUA male	rs571848662*	19	53988841	t	tatac	0.608	-0.139	0.024	0.87	7.9×10^{-9}	NA*	NA	NA	NA	6,263	22,971
EUA male	rs8112292**	19	53989669	t	c	0.464	0.096	0.022	1.10	1.0×10^{-5}	0.092	0.022	1.10	2.1×10^{-5}	9,799	74,615
EUA male	rs148757321	1	15436223	ctgtg	c	0.828	0.168	0.031	1.18	3.8×10^{-8}	0.180	0.031	1.20	5.5×10^{-9}	6,263	22,971

Leading markers for genome-wide significant loci (at p<5x10-8) in the overall and sex-stratified analyses are reported.

A1 allele 1 (coded allele), A2 allele2, A1 freq A1 allele frequency, *Beta_cojo* Beta value conditioned on summary data from the PGC-PTSD major depressive disorder (MDD) GWAS, using multi-trait conditional and joint analysis (mtCOJO), *OR_cojo* Odds ratio conditioned on PGC-PTSD MDD, *SE_cojo* Standard error of beta conditioned on PGC-PTSD MDD, *P_cojo* p-value of beta conditioned on PGC-PTSD MDD
*rs571848662 is not in MDD data
** rs8112292 is the proxy SNP in highest LD (r^2 = 0.85 in 1000G CEU) with rs571848662

Supplementary Table 11 Meta-analysis of European ancestry GWAS, before and after conditioning on MDD, BPD, and SCZ

Subjects	Variant	Chr	Position (bp)	A1	A2	A1 freq	Beta	SE	OR	*P*-value	Beta_cojo	SE_cojo	OR_cojo	*P*_cojo	*N* cases	*N* controls
EUA all	rs34517852	6	157789333	a	t	0.341	0.110	0.019	1.12	3.2×10^{-9}	0.115	0.019	1.12	1.1×10^{-9}	12,080	33,446
EUA all	rs9364611	6	162163506	t	c	0.131	-0.124	0.023	0.88	4.4×10^{-8}	-0.132	0.023	0.88	9.7×10^{-9}	23,212	151,447
EUA male	rs571848662*	19	53988841	t	tatac	0.608	-0.139	0.024	0.87	7.9×10^{-9}	NA*	NA	NA	NA	6,263	22,971
EUA male	rs8112292**	19	53989669	t	c	0.464	0.096	0.022	1.10	1.0×10^{-5}	0.080	0.022	1.08	3.9×10^{-4}	9,799	74,615
EUA male	rs148757321***	1	15436223	ctgtg	c	0.828	0.168	0.031	1.18	3.8×10^{-8}	NA***	NA	NA	NA	6,263	22,971
EUA male	rs518152****	1	15434259	a	g	0.171	-0.146	0.029	0.86	3.4×10^{-7}	-0.146	0.030	0.86	8.6×10^{-7}	9,900	75,549

Leading markers for genome-wide significant loci (at p<5x10-8) in the overall and sex-stratified analyses are reported.

A1 allele 1 (coded allele), *A2* allele2, *A1 freq* A1 allele frequency, *Beta_cojo* Beta value conditioned on summary data from the PGC-PTSD bipolar disorder (BPD), *major depressive disorder (MDD) and schizophrenia (SCZ) GWAS, using multi-trait conditional and joint analysis (mtCOJO), OR_cojo* Odds ratio conditioned on BPD, MDD and SCZ, *SE_cojo Standard error of beta conditioned on BPD, MDD and SCZ, P_cojo p*-value of beta conditioned on BPD, MDD and SCZ
*rs571848662 is not in in all summary statistic datasets
** rs8112292 is the proxy SNP in highest LD (r2=0.85 in 1000G CEU) with rs571848662
***rs148757321 is not in all summary statistic datasets
**** rs518152 is the proxy SNP in highest LD (r^2=0.96 in 1000G CEU) with rs148757321

Supplementary Table 12 Global ancestry determination and assignment into 3 large, homogenous groups for GWAS analysis

Ancestry grouping	*N* cases*	*N* controls*	*N* total*	% total	GWAS grouping
Continental regions:					
Africa	82	151	233	0.34	African ancestry (AFA)
Europe	10,643	28,633	39,276	56.53	European ancestry (EUA)
Central/South Asia	8	62	70	0.10	excluded
East Asia	92	436	528	0.76	excluded
Americas	642	679	1,321	1.90	American ancestry (AMA)
Oceania	15	75	90	0.13	excluded
Mixed ancestry:					
Africa/Europe (mostly African American)	4,428	12,456	16,884	24.30	African ancestry (AFA)
Americas/Europe (mostly Latinos)	1,544	3,486	5,030	7.24	American ancestry (AMA)
East Asia/Europe (mostly Filipinos)	58	212	270	0.39	excluded
Europe/Americas/Africa (mostly Puerto Rican)	303	1,163	1,466	2.11	excluded
Others	1,125	3,191	4,316	6.21	excluded
Total	18,940	50,544	69,484	100	

* only subjects with available individual-level genotype data are included here

Supplementary Table 13 Cell-lines used for functional follow-up of rs115539978

Cell Line	Genotype*	Gender	Population**
HG02545	T/T	M	ACB
HG03376	T/T	M	MSL
GM20281	T/T	M	ASW
HG02703	T/T	F	GWD
HG03114	T/T	F	ESN
GM19351	T/T	F	LWK
HG01879	C/C	M	ACB
HG03057	C/C	M	MSL
GM19700	C/C	M	ASW
HG02462	C/C	F	GWD
HG02922	C/C	F	ESN
GM19017	C/C	F	LWK

All populations belong to the AFR super population

**genotype for rs115539978, T: minor allele, C: major allele*
***Population abbreviations: ACB* African Caribbeans in Barbados, *ASW* Americans of African Ancestry in SW USA, *ESN* Esan in Nigeria*, GWD* Gambian in Western Divisions in the Gambia, *LWK* Luhya in Webuye, Kenya, *MSL* Mende in Sierra Leone

Supplementary Table 14 Primers used for functional follow-up of rs115539978

Primer Name	Sequence 5'-3'
uc_338-fwd1	TCCCATCTGCTCAAACCACT
uc_338-rev1	CCTCTCAAGAGAAAGACAAAGG
Linc02335ex1ex2-fwd1	GCCACTGCTTTCAGCCTTTA
Linc02335ex1ex2-rev1	ATCAGTCTTTCTCAGGAAGTAGACA
Linc00558fwd1	CTCCGCTAACACACACTTTCA
Linc00558rev1	CCATCCTTTTACTTCCAGCCTA
Linc00458fwd1	ACAGTCCTCAGCCTCCTGAA
Linc00458rev1	TGGGTTGGTGTTCTCCTCTC
hsaGAPDHfwd	AGCTCAGGGATGACCTTGC
hsaGAPDHfwd	TCACTGCCACCCAGAAGACT

Supplementary References

1 Kessler, R. C. *et al.* Design of the army study to assess risk and resilience in servicemembers (Army STARRS). *International Journal of Methods in Psychiatric Research* **22**, 267-275 (2013).

2 Weathers, F. W., Litz, B. T., Herman, D. S., Huska, J. A. & Keane, T. M. in *annual convention of the international society for traumatic stress studies, San Antonio, TX.* (San Antonio, TX.).

3 Kessler, R. C. *et al.* Clinical reappraisal of the Composite International Diagnostic Interview Screening Scales (CIDI-SC) in the Army Study to Assess Risk and Resilience in Servicemembers (Army STARRS). *International journal of methods in psychiatric research* **22**, 303-321 (2013).

4 McFarlane, A. C. & Van Hooff, M. Impact of childhood exposure to a natural disaster on adult mental health: 20-year longitudinal follow-up study. *Br J Psychiatry* **195**, 142-148, doi:10.1192/bjp.bp.108.054270 (2009).

5 Brugha, T., Bebbington, P., Tennant, C. & Hurry, J. The List of Threatening Experiences: a subset of 12 life event categories with considerable long-term contextual threat. *Psychol Med* **15**, 189-194 (1985).

6 van Rooij, S. J., Kennis, M., Vink, M. & Geuze, E. Predicting Treatment Outcome in PTSD: A Longitudinal Functional MRI Study on Trauma-Unrelated Emotional Processing. *Neuropsychopharmacology* **41**, 1156-1165, doi:10.1038/npp.2015.257 (2016).

7 Blake, D. D. *et al.* The development of a Clinician-Administered PTSD Scale. *J Trauma Stress* **8**, 75-90 (1995).

8 First, M. B. & Gibbon, M. The Structured Clinical Interview for DSM-IV Axis I Disorders (SCID-I) and the Structured Clinical Interview for DSM-IV Axis II Disorders (SCID-II). (2004).

9 Gray, M. J., Litz, B. T., Hsu, J. L. & Lombardo, T. W. Psychometric properties of the life events checklist. *Assessment* **11**, 330-341, doi:10.1177/1073191104269954 (2004).

10 Ruggiero, K. J. *et al.* Bounce Back Now! Protocol of a population-based randomized controlled trial to examine the efficacy of a Web-based intervention with disaster-affected families. *Contemp Clin Trials* **40**, 138-149, doi:10.1016/j.cct.2014.11.018 (2015).

11 Kilpatrick, D. G. *et al.* Risk factors for adolescent substance abuse and dependence: data from a national sample. *J Consult Clin Psychol* **68**, 19-30 (2000).

12 Sartor, C. E. *et al.* Common heritable contributions to low-risk trauma, high-risk trauma, posttraumatic stress disorder, and major depression. *Arch Gen Psychiatry* **69**, 293-299, doi:10.1001/archgenpsychiatry.2011.1385 (2012).

13 Kristjansson, S. *et al.* The variance shared across forms of childhood trauma is strongly associated with liability for psychiatric and substance use disorders. *Brain Behav* **6**, e00432, doi:10.1002/brb3.432 (2016).

14 Nelson, E. C., Lynskey, M. T., Heath, A. C., Madden, P. A. & Martin, N. G. A family study of adult twins with and without a history of childhood abuse: stability of retrospective reports of maltreatment and associated family measures. *Twin research and human genetics : the official journal of the International Society for Twin Studies* **13**, 121-130, doi:10.1375/twin.13.2.121 (2010).

15 Kessler, R. C., Sonnega, A., Bromet, E., Hughes, M. & Nelson, C. B. Posttraumatic stress disorder in the National Comorbidity Survey. *Archives of general psychiatry* **52**, 1048-1060 (1995).

16 Steinberg, A. M., Brymer, M. J., Decker, K. B. & Pynoos, R. S. The University of California at Los Angeles post-traumatic stress disorder reaction index. *Current psychiatry reports* **6**, 96-100 (2004).

17 Bifulco, A., Brown, G. W. & Harris, T. O. Childhood Experience of Care and Abuse (CECA): a retrospective interview measure. *Journal of Child Psychology and Psychiatry* **35**, 1419-1435 (1994).

18 Bernstein, D. P., Ahluvalia, T., Pogge, D. & Handelsman, L. Validity of the Childhood Trauma Questionnaire in an adolescent psychiatric population. *J Am Acad Child Adolesc Psychiatry* **36**, 340-348, doi:10.1097/00004583-199703000-00012 (1997).

19 Nader, K. O. *et al.* Clinician Administered PTSD scale for children and adolescents. *National Center for PTSD* (1996).
20 Bedard-Gilligan, M., Zoellner, L. A. & Feeny, N. C. Is Trauma Memory Special? Trauma Narrative Fragmentation in PTSD: Effects of Treatment and Response. *Clinical Psychological Science* **5**, 212-225 (2017).
21 Cooper, A. A. *et al.* Homework “Dose,” Type, and Helpfulness as Predictors of Clinical Outcomes in Prolonged Exposure for PTSD. *Behavior Therapy* **48**, 182-194 (2017).
22 Clifton, E. G., Feeny, N. C. & Zoellner, L. A. Anger and guilt in treatment for chronic posttraumatic stress disorder. *Journal of behavior therapy and experimental psychiatry* **54**, 9-16 (2017).
23 Jerud, A. B. *et al.* Repeated trauma exposure does not impair distress reduction during imaginal exposure for posttraumatic stress disorder. *Depression and anxiety* **34**, 671-678 (2017).
24 Echiverri-Cohen, A. *et al.* Changes in temporal attention inhibition following prolonged exposure and sertraline in the treatment of PTSD. *Journal of consulting and clinical psychology* **84**, 415 (2016).
25 Jerud, A. B., Pruitt, L. D., Zoellner, L. A. & Feeny, N. C. The effects of prolonged exposure and sertraline on emotion regulation in individuals with posttraumatic stress disorder. *Behaviour research and therapy* **77**, 62-67 (2016).
26 Post, L. M., Feeny, N. C., Zoellner, L. A. & Connell, A. M. Post-traumatic stress disorder and depression co-occurrence: Structural relations among disorder constructs and trait and symptom dimensions. *Psychology and Psychotherapy: Theory, Research and Practice* **89**, 418-434 (2016).
27 Bedard-Gilligan, M. *et al.* An Investigation of Depression, Trauma History, and Symptom Severity in Individuals Enrolled in a Treatment Trial for Chronic PTSD. *J Clin Psychol* **71**, 725-740, doi:10.1002/jclp.22163 (2015).
28 Jerud, A. B., Zoellner, L. A., Pruitt, L. D. & Feeny, N. C. Changes in Emotion Regulation in Adults With and Without a History of Childhood Abuse Following Posttraumatic Stress Disorder Treatment (vol 82, pg 721, 2014). *Journal of Consulting and Clinical Psychology* **82**, 827-827, doi:10.1037/a0037947 (2014).
29 Keller, S. M., Feeny, N. C. & Zoellner, L. A. Depression Sudden Gains and Transient Depression Spikes During Treatment for PTSD. *Journal of Consulting and Clinical Psychology* **82**, 102-111, doi:10.1037/a0035286 (2014).
30 Resnick, H., Best, C., Kilpatrick, D., Freedy, J. & Falsetti, S. Trauma assessment for adults. *Charleston, SC: Medical University of South Carolina* (1993).
31 Foa, E. B., Riggs, D. S., Dancu, C. V. & Rothbaum, B. O. Reliability and validity of a brief instrument for assessing post-traumatic stress disorder. *Journal of traumatic stress* **6**, 459-473 (1993).
32 Almli, L. M. *et al.* Problematic alcohol use associates with sodium channel and clathrin linker 1 (SCLT1) in trauma-exposed populations. *Addict Biol*, doi:10.1111/adb.12569 (2017).
33 Lindqvist, D. *et al.* Increased circulating blood cell counts in combat-related PTSD: Associations with inflammation and PTSD severity. *Psychiatry Res* **258**, 330-336, doi:10.1016/j.psychres.2017.08.052 (2017).
34 Hammamieh, R. *et al.* Whole-genome DNA methylation status associated with clinical PTSD measures of OIF/OEF veterans. *Transl Psychiatry* **7**, e1169, doi:10.1038/tp.2017.129 (2017).
35 Keane, T. M. *et al.* Clinical evaluation of a measure to assess combat exposure. *Psychological Assessment: A Journal of Consulting and Clinical Psychology* **1**, 53 (1989).
36 Karstoft, K. I., Andersen, S. B. & Nielsen, A. B. S. Assessing PTSD in the military: Validation of a scale distributed to Danish soldiers after deployment since 1998. *Scand J Psychol* **58**, 260-268, doi:10.1111/sjop.12360 (2017).

37 Pedersen, C. B. *et al.* The iPSYCH2012 case-cohort sample: new directions for unravelling genetic and environmental architectures of severe mental disorders. *Mol Psychiatry* **23**, 6-14, doi:10.1038/mp.2017.196 (2018).
38 Hollegaard, M. V. *et al.* Robustness of genome-wide scanning using archived dried blood spot samples as a DNA source. *BMC Genet* **12**, 58, doi:10.1186/1471-2156-12-58 (2011).
39 Borglum, A. D. *et al.* Genome-wide study of association and interaction with maternal cytomegalovirus infection suggests new schizophrenia loci. *Mol Psychiatry* **19**, 325-333, doi:10.1038/mp.2013.2 (2014).
40 Gandal, M. J. *et al.* Shared molecular neuropathology across major psychiatric disorders parallels polygenic overlap. *Science* **359**, 693-697 (2018).
41 Norrholm, S. D. *et al.* Baseline psychophysiological and cortisol reactivity as a predictor of PTSD treatment outcome in virtual reality exposure therapy. *Behav Res Ther* **82**, 28-37, doi:10.1016/j.brat.2016.05.002 (2016).
42 Rothbaum, B. O. *et al.* A randomized, double-blind evaluation of D-cycloserine or alprazolam combined with virtual reality exposure therapy for posttraumatic stress disorder in Iraq and Afghanistan War veterans. *Am J Psychiatry* **171**, 640-648, doi:10.1176/appi.ajp.2014.13121625 (2014).
43 Gillespie, C. F. *et al.* Trauma exposure and stress-related disorders in inner city primary care patients. *Gen Hosp Psychiatry* **31**, 505-514, doi:10.1016/j.genhosppsych.2009.05.003 (2009).
44 Disner, S. G. *et al.* Predictors of Postdeployment Functioning in Combat-Exposed U.S. Military Veterans. *Clinical Psychological Science* **5**, 650-663, doi:10.1177/2167702617703436 (2017).
45 Davenport, N. D., Lim, K. O. & Sponheim, S. R. Personality and neuroimaging measures differentiate PTSD from mTBI in veterans. *Brain Imaging Behav* **9**, 472-483, doi:10.1007/s11682-015-9371-y (2015).
46 Vogt, D. *et al.* Deployment risk and resilience inventory-2 (DRRI-2): an updated tool for assessing psychosocial risk and resilience factors among service members and veterans. *J Trauma Stress* **26**, 710-717 (2013).
47 Uddin, M. *et al.* Epigenetic and immune function profiles associated with posttraumatic stress disorder. *Proc Natl Acad Sci U S A* **107**, 9470-9475, doi:10.1073/pnas.0910794107 (2010).
48 Breslau, N. *et al.* Trauma and posttraumatic stress disorder in the community: the 1996 Detroit Area Survey of Trauma. *Arch Gen Psychiatry* **55**, 626-632 (1998).
49 Blanchard, E. B., Jones-Alexander, J., Buckley, T. C. & Forneris, C. A. Psychometric properties of the PTSD Checklist (PCL). *Behav Res Ther* **34**, 669-673 (1996).
50 Stein, D. J. *et al.* Investigating the psychosocial determinants of child health in Africa: The Drakenstein Child Health Study. *J Neurosci Methods* **252**, 27-35, doi:10.1016/j.jneumeth.2015.03.016 (2015).
51 Zar, H. J., Barnett, W., Myer, L., Stein, D. J. & Nicol, M. P. Investigating the early-life determinants of illness in Africa: the Drakenstein Child Health Study. *Thorax* **70**, 592-594, doi:10.1136/thoraxjnl-2014-206242 (2015).
52 Koen, N. *et al.* Intimate partner violence: associations with low infant birthweight in a South African birth cohort. *Metab Brain Dis* **29**, 281-299, doi:10.1007/s11011-014-9525-4 (2014).
53 Platts-Mills, T. F. *et al.* Using emergency department-based inception cohorts to determine genetic characteristics associated with long term patient outcomes after motor vehicle collision: methodology of the CRASH study. *BMC emergency medicine* **11**, 14 (2011).
54 Weiss, D. S. in *Cross-cultural assessment of psychological trauma and PTSD* 219-238 (Springer, 2007).
55 Afful, S. E., Strickland, J. R., Cottler, L. & Bierut, L. J. Exposure to trauma: a comparison of cocaine-dependent cases and a community-matched sample. *Drug Alcohol Depend* **112**, 46-53, doi:10.1016/j.drugalcdep.2010.05.012 (2010).
56 Bierut, L. J. *et al.* Novel genes identified in a high-density genome wide association study for nicotine dependence. *Hum Mol Genet* **16**, 24-35, doi:10.1093/hmg/ddl441 (2007).

57 Robins, L., Cottler, L., Bucholz, K. & Compton, W. *Diagnostic interview schedule for dsm-iv (dis-iv)*. (1995).
58 Association, A. P. Diagnostic and statistical manual of mental disorders-IV-TR. *Washington, DC: American Psychiatric Association* (2000).
59 Weathers, F. W. *et al.* The PTSD checklist for DSM-5 (PCL-5). 2013. *Scale available at from the National Center for PTSD at http://www. ptsd. va. gov* (2014).
60 Taylor, D. J. *et al.* Prevalence, Correlates, and Predictors of Insomnia in the US Army prior to Deployment. *Sleep* **39**, 1795-1806, doi:10.5665/sleep.6156 (2016).
61 Yehuda, R. *et al.* Glucocorticoid-related predictors and correlates of post-traumatic stress disorder treatment response in combat veterans. *Interface Focus* **4**, 20140048, doi:10.1098/rsfs.2014.0048 (2014).
62 Yehuda, R. *et al.* Epigenetic biomarkers as predictors and correlates of symptom improvement following psychotherapy in combat veterans with PTSD. *Frontiers in psychiatry* **4** (2013).
63 Allen, J. G. User's guide for the structured clinical interview for DSM-IV Axis II personality disorders: SCID-II. *Bulletin of the Menninger Clinic* **62**, 547 (1998).
64 Sheehan, D. V. *et al.* The Mini-International Neuropsychiatric Interview (M.I.N.I.): the development and validation of a structured diagnostic psychiatric interview for DSM-IV and ICD-10. *J Clin Psychiatry* **59 Suppl 20**, 22-33;quiz 34-57 (1998).
65 Junglen, A. G., Hruska, B., Jensen, T., Boros, A., & Delahanty, D. L. . The Serial Mediating Effects of Negative Urgency and PTSD Symptom Severity on the Relationship Between Emotional Abuse and Substance Use. *Manuscript in preparation* (2017).
66 Weathers, F. *et al.* The life events checklist for DSM-5 (LEC-5). *Instrument available from the National Center for PTSD at www. ptsd. va. gov* (2013).
67 Mehta, D. *et al.* Genomewide DNA methylation analysis in combat veterans reveals a novel locus for PTSD. *Acta Psychiatrica Scandinavica* **136**, 493-505 (2017).
68 Weathers, F. *et al.* The clinician-administered PTSD scale for DSM-5 (CAPS-5). *Interview available from the National Center for PTSD at www. ptsd. va. gov* (2013).
69 Seng, J. S., Low, L. K., Sperlich, M., Ronis, D. L. & Liberzon, I. Post-traumatic stress disorder, child abuse history, birthweight and gestational age: a prospective cohort study. *BJOG* **118**, 1329-1339, doi:10.1111/j.1471-0528.2011.03071.x (2011).
70 Wolfe, J. & Kimerling, R. Gender issues in the assessment of posttraumatic stress disorder. (1997).
71 Resnick, H. S., Kilpatrick, D. G., Dansky, B. S., Saunders, B. E. & Best, C. L. Prevalence of civilian trauma and posttraumatic stress disorder in a representative national sample of women. *J Consult Clin Psychol* **61**, 984-991 (1993).
72 Kilpatrick, D. G., Resnick, H., Saunders, B. & Best, C. The national women's study PTSD module. *Unpublished instrument. Charleston, SC: Crime Victims Research and Treatment Center, Department of Psychiatry and Behavioral Sciences, Medical University of South Carolina* (1989).
73 Binder, E. B. *et al.* Association of FKBP5 polymorphisms and childhood abuse with risk of posttraumatic stress disorder symptoms in adults. *JAMA* **299**, 1291-1305, doi:10.1001/jama.299.11.1291 (2008).
74 Falsetti, S. A., Resnick, H. S., Resick, P. A. & Kilpatrick, D. G. The modified PTSD symptom scale: a brief self-report measure of posttraumatic stress disorder. *The Behavior Therapist* (1993).
75 Roberts, A. L., Rosario, M., Corliss, H. L., Koenen, K. C. & Austin, S. B. Childhood gender nonconformity: a risk indicator for childhood abuse and posttraumatic stress in youth. *Pediatrics* **129**, 410-417, doi:10.1542/peds.2011-1804 (2012).
76 Schnurr, P., Vieilhauer, M. & Weathers, F. (National Center for PTSD, White River Junction, 1999).

77 Breslau, N., Peterson, E. L., Kessler, R. C. & Schultz, L. R. Short screening scale for DSM-IV posttruamatic stress disorder. *American Journal of Psychiatry* **156**, 908-911 (1999).
78 McAllister, T. W. *et al.* Randomized placebo-controlled trial of methylphenidate or galantamine for persistent emotional and cognitive symptoms associated with PTSD and/or traumatic brain injury. *Neuropsychopharmacology* **41**, 1191 (2016).
79 Rasmusson, A. M. *et al.* A randomized controlled trial of ganaxolone in posttraumatic stress disorder. *Psychopharmacology* **234**, 2245-2257 (2017).
80 Lepage, C. *et al.* White matter abnormalities in mild traumatic brain injury with and without post-traumatic stress disorder: a subject-specific diffusion tensor imaging study. *Brain imaging and behavior*, 1-12 (2017).
81 Vogt, D. S., Proctor, S. P., King, D. W., King, L. A. & Vasterling, J. J. Validation of scales from the Deployment Risk and Resilience Inventory in a sample of Operation Iraqi Freedom veterans. *Assessment* **15**, 391-403 (2008).
82 Wilkins, K. C., Lang, A. J. & Norman, S. B. Synthesis of the psychometric properties of the PTSD checklist (PCL) military, civilian, and specific versions. *Depression and anxiety* **28**, 596-606 (2011).
83 Bryant, R. A. *et al.* The psychiatric sequelae of traumatic injury. *Am J Psychiatry* **167**, 312-320, doi:10.1176/appi.ajp.2009.09050617 (2010).
84 Nievergelt, C. M. *et al.* Genomic predictors of combat stress vulnerability and resilience in U.S. Marines: A genome-wide association study across multiple ancestries implicates PRTFDC1 as a potential PTSD gene. *Psychoneuroendocrinology* **51**, 459-471, doi:10.1016/j.psyneuen.2014.10.017 (2015).
85 Baker, D. G. *et al.* Predictors of risk and resilience for posttraumatic stress disorder among ground combat Marines: methods of the Marine Resiliency Study. *Prev Chronic Dis* **9**, E97 (2012).
86 Khan, A. *et al.* Childhood maltreatment, depression, and suicidal ideation: critical importance of parental and peer emotional abuse during developmental sensitive periods in males and females. *Frontiers in psychiatry* **6**, 42 (2015).
87 Teicher, M. H. *et al.* Differential effects of childhood neglect and abuse during sensitive exposure periods on male and female hippocampus. *NeuroImage* (2017).
88 Ohashi, K. *et al.* Childhood maltreatment is associated with alteration in global network fiber-tract architecture independent of history of depression and anxiety. *NeuroImage* **150**, 50-59 (2017).
89 Teicher, M. H. & Parigger, A. The 'Maltreatment and Abuse Chronology of Exposure'(MACE) scale for the retrospective assessment of abuse and neglect during development. *PLoS one* **10**, e0117423 (2015).
90 Herman, J., Perry, J. & van der Kolk, B. Traumatic antecedents interview. *Boston: The Trauma Center* (1989).
91 Bernstein, D. P. *et al.* Initial reliability and validity of a new retrospective measure of child abuse and neglect. *The American journal of psychiatry* **151**, 1132 (1994).
92 Anda, R. F. *et al.* The enduring effects of abuse and related adverse experiences in childhood. *European archives of psychiatry and clinical neuroscience* **256**, 174-186 (2006).
93 DATA, D. *Structured clinical interview for DSM-IV axis I disorders*. (American Psychiatric Press, Washington, 1997).
94 Ashley-Koch, A. E. *et al.* Genome-wide association study of posttraumatic stress disorder in a cohort of Iraq-Afghanistan era veterans. *J Affect Disord* **184**, 225-234, doi:10.1016/j.jad.2015.03.049 (2015).
95 Liu, Y. *et al.* Association of Variant rs4790904 in Protein Kinase C Alpha with Posttraumatic Stress Disorder in a U.S. Caucasian and African-American Veteran Sample. *J Depress Anxiety* **2**, S4001 (2013).
96 Brewin, C. R. *et al.* Brief screening instrument for post-traumatic stress disorder. *Br J Psychiatry* **181**, 158-162 (2002).

97 Watkins, L. E. *et al.* FKBP5 polymorphisms, childhood abuse, and PTSD symptoms: Results from the National Health and Resilience in Veterans Study. *Psychoneuroendocrinology* **69**, 98-105, doi:10.1016/j.psyneuen.2016.04.001 (2016).
98 Carlson, E. B. *et al.* Development and validation of a brief self-report measure of trauma exposure: the Trauma History Screen. *Psychol Assess* **23**, 463-477, doi:10.1037/a0022294 (2011).
99 Stephenson, K. L., Valentiner, D. P., Kumpula, M. J. & Orcutt, H. K. Anxiety sensitivity and posttrauma stress symptoms in female undergraduates following a campus shooting. *J Trauma Stress* **22**, 489-496, doi:10.1002/jts.20457 (2009).
100 Mercer, K. B. *et al.* Acute and posttraumatic stress symptoms in a prospective gene x environment study of a university campus shooting. *Arch Gen Psychiatry* **69**, 89-97, doi:10.1001/archgenpsychiatry.2011.109 (2012).
101 Kubany, E. Traumatic Life Events Questionnaire and PTSD Screening and Diagnostic Scale. *Los Angeles, CA: Western Psychological Services* (2004).
102 Kubany, E. S., Leisen, M. B., Kaplan, A. S. & Kelly, M. P. Validation of a brief measure of posttraumatic stress disorder: the Distressing Event Questionnaire (DEQ). *Psychological assessment* **12**, 197 (2000).
103 Koenen, K. C. *et al.* Protocol for investigating genetic determinants of posttraumatic stress disorder in women from the Nurses' Health Study II. *BMC Psychiatry* **9**, 29, doi:10.1186/1471-244X-9-29 (2009).
104 Kessler, R. C. *et al.* Clinical calibration of DSM-IV diagnoses in the World Mental Health (WMH) version of the World Health Organization (WHO) Composite International Diagnostic Interview (WMHCIDI). *Int J Methods Psychiatr Res* **13**, 122-139 (2004).
105 Helzer, J. E. *et al.* A comparison of clinical and diagnostic interview schedule diagnoses. Physician reexamination of lay-interviewed cases in the general population. *Archives of General Psychiatry* **42**, 657-666 (1985).
106 Robins, L. N. Epidemiology: reflections on testing the validity of psychiatric interviews. *Archives of General Psychiatry* **42**, 918-924 (1985).
107 Robins, L. N., Helzer, J. E., Cottler, L. & Golding, E. *National Institute of Mental Health diagnostic interview schedule version III - revised*. (Department of Psychiatry, Washington University, 1988).
108 Weathers, F. & Ford, J. Psychometric review of PTSD Checklist (PCL-C, PCL-S, PCL-M, PCL-PR). *Measurement of stress, trauma, and adaptation*, 250-251 (1996).
109 Roberts, A. L. *et al.* The stressor criterion for posttraumatic stress disorder: does it matter? *J Clin Psychiatry* **73**, e264-270, doi:10.4088/JCP.11m07054 (2012).
110 Roberts, A. L. *et al.* Association of Trauma and Posttraumatic Stress Disorder With Incident Systemic Lupus Erythematosus in a Longitudinal Cohort of Women. *Arthritis Rheumatol* **69**, 2162-2169, doi:10.1002/art.40222 (2017).
111 Liberzon, I. *et al.* Interaction of the ADRB2 gene polymorphism with childhood trauma in predicting adult symptoms of posttraumatic stress disorder. *JAMA Psychiatry* **71**, 1174-1182, doi:10.1001/jamapsychiatry.2014.999 (2014).
112 Prescott, M. R. *et al.* Validation of lay-administered mental health assessments in a large Army National Guard cohort. *Int J Methods Psychiatr Res* **23**, 109-119, doi:10.1002/mpr.1416 (2014).
113 Xavier, M., Baptista, H., Mendes, J. M., Magalhaes, P. & Caldas-de-Almeida, J. M. Implementing the World Mental Health Survey Initiative in Portugal - rationale, design and fieldwork procedures. *Int J Ment Health Syst* **7**, 19, doi:10.1186/1752-4458-7-19 (2013).
114 Reijnen, A., Rademaker, A. R., Vermetten, E. & Geuze, E. Prevalence of mental health symptoms in Dutch military personnel returning from deployment to Afghanistan: a 2-year longitudinal analysis. *Eur Psychiatry* **30**, 341-346, doi:10.1016/j.eurpsy.2014.05.003 (2015).
115 Hovens, J. E. *et al.* The development of the Self-Rating Inventory for Posttraumatic Stress Disorder. *Acta Psychiatr Scand* **90**, 172-183 (1994).

116 Barrios, Y. V. *et al.* Association of childhood physical and sexual abuse with intimate partner violence, poor general health and depressive symptoms among pregnant women. *PLoS One* **10**, e0116609, doi:10.1371/journal.pone.0116609 (2015).
117 Measure, D. (2005).
118 Garcia-Moreno, C. *et al.* Prevalence of intimate partner violence: findings from the WHO multi-country study on women's health and domestic violence. *Lancet* **368**, 1260-1269, doi:10.1016/S0140-6736(06)69523-8 (2006).
119 Felitti, V. J. *et al.* Relationship of childhood abuse and household dysfunction to many of the leading causes of death in adults. The Adverse Childhood Experiences (ACE) Study. *Am J Prev Med* **14**, 245-258 (1998).
120 Gelaye, B. *et al.* Validity of the posttraumatic stress disorders (PTSD) checklist in pregnant women. *BMC Psychiatry* **17**, 179, doi:10.1186/s12888-017-1304-4 (2017).
121 Polusny, M. A. *et al.* Prospective risk factors for new-onset post-traumatic stress disorder in National Guard soldiers deployed to Iraq. *Psychological medicine* **41**, 687-698 (2011).
122 Bliese, P. D. *et al.* Validating the primary care posttraumatic stress disorder screen and the posttraumatic stress disorder checklist with soldiers returning from combat. *J Consult Clin Psychol* **76**, 272-281, doi:10.1037/0022-006X.76.2.272 (2008).
123 Norman, S. B. *et al.* Maximizing the utility of a single site randomized controlled psychotherapy trial. *Contemp Clin Trials* **42**, 244-251, doi:10.1016/j.cct.2015.04.011 (2015).
124 Association, A. P. *Diagnostic and statistical manual of mental disorders (DSM-5®)*. (American Psychiatric Pub, 2013).
125 Dzubur-Kulenovic, A. *et al.* MOLECULAR MECHANISMS OF POSTTRAUMATIC STRESS DISORDER (PTSD) AS A BASIS FOR INDIVIDUALIZED AND PERSONALIZED THERAPY: RATIONALE, DESIGN AND METHODS OF THE SOUTH EASTERN EUROPE (SEE)-PTSD STUDY. *Psychiatria Danubina* **28**, 154-163 (2016).
126 Priebe, S. *et al.* Mental disorders following war in the Balkans: a study in 5 countries. *Archives of general psychiatry* **67**, 518-528 (2010).
127 Davenport, N. D., Lim, K. O. & Sponheim, S. R. White matter abnormalities associated with military PTSD in the context of blast TBI. *Hum Brain Mapp* **36**, 1053-1064, doi:10.1002/hbm.22685 (2015).
128 Logue, M. W. *et al.* A genome-wide association study of post-traumatic stress disorder identifies the retinoid-related orphan receptor alpha (RORA) gene as a significant risk locus. *Mol Psychiatry* **18**, 937-942, doi:10.1038/mp.2012.113 (2013).
129 Chen, C. Y. *et al.* Improved ancestry inference using weights from external reference panels. *Bioinformatics* **29**, 1399-1406, doi:10.1093/bioinformatics/btt144 (2013).
130 Sadeh, N. *et al.* SKA2 methylation is associated with decreased prefrontal cortical thickness and greater PTSD severity among trauma-exposed veterans. *Mol Psychiatry* **21**, 357-363, doi:10.1038/mp.2015.134 (2016).
131 Howie, B. N., Donnelly, P. & Marchini, J. A flexible and accurate genotype imputation method for the next generation of genome-wide association studies. *PLoS Genet* **5**, e1000529, doi:10.1371/journal.pgen.1000529 (2009).
132 Howie, B., Marchini, J. & Stephens, M. Genotype imputation with thousands of genomes. *G3 (Bethesda)* **1**, 457-470, doi:10.1534/g3.111.001198 (2011).
133 Howie, B., Fuchsberger, C., Stephens, M., Marchini, J. & Abecasis, G. R. Fast and accurate genotype imputation in genome-wide association studies through pre-phasing. *Nat Genet* **44**, 955-959, doi:10.1038/ng.2354 (2012).
134 Marchini, J. & Howie, B. Genotype imputation for genome-wide association studies. *Nat Rev Genet* **11**, 499-511, doi:10.1038/nrg2796 (2010).
135 Genomes Project, C. *et al.* An integrated map of genetic variation from 1,092 human genomes. *Nature* **491**, 56-65, doi:10.1038/nature11632 (2012).
136 Price, A. L. *et al.* Principal components analysis corrects for stratification in genome-wide association studies. *Nat Genet* **38**, 904-909, doi:10.1038/ng1847 (2006).

137 Allen, N. E., Sudlow, C., Peakman, T. & Collins, R. (American Association for the Advancement of Science, 2014).
138 Davis, K. *et al.* Mental Health in UK Biobank–implementation and results of an online questionnaire in 157,366 participants. (2018).
139 Bycroft, C. *et al.* Genome-wide genetic data on~ 500,000 UK Biobank participants. *Preprint at https://www.biorxiv.org/content/10.1101/166298v1.abstract* (2017).
140 Warren, H. R. *et al.* Genome-wide association analysis identifies novel blood pressure loci and offers biological insights into cardiovascular risk. *Nature genetics* **49**, 403 (2017).
141 Abraham, G., Qiu, Y. & Inouye, M. FlashPCA2: principal component analysis of Biobank-scale genotype datasets. *Bioinformatics* **33**, 2776-2778 (2017).
142 McCarthy, S. *et al.* A reference panel of 64,976 haplotypes for genotype imputation. *Nature genetics* **48**, 1279 (2016).
143 Consortium, U. K. The UK10K project identifies rare variants in health and disease. *Nature* **526**, 82 (2015).
144 Kremen, W. S. *et al.* Genes, environment, and time: the Vietnam Era Twin Study of Aging (VETSA). *Twin research and human genetics : the official journal of the International Society for Twin Studies* **9**, 1009-1022, doi:10.1375/183242706779462750 (2006).
145 Kremen, W. S., Franz, C. E. & Lyons, M. J. VETSA: the Vietnam Era Twin Study of Aging. *Twin research and human genetics : the official journal of the International Society for Twin Studies* **16**, 399-402, doi:10.1017/thg.2012.86 (2013).
146 Janes, G. R., Goldberg, J., Eisen, S. A. & True, W. R. Reliability and validity of a combat exposure index for Vietnam era veterans. *Journal of Clinical Psychology* **47**, 80-86 (1991).
147 Robins, L. N., Helzer, J. E., Croughan, J. & Ratcliff, K. S. National Institute of Mental Health Diagnostic Interview Schedule. Its history, characteristics, and validity. *Archives of General Psychiatry* **38**, 381-389 (1981).
148 Goldberg, J., Eisen, S. A., Trub, W. R. & Henderson, W. G. A twin study of the effects of the Vietnam conflict on alcohol drinking patterns. *American Journal of Public Health* **80**, 570-574 (1990).
149 Weathers, F. W., Litz, B. T., Huska, J. A. & Keane, T. M. (National Center for PTSD - Behavioral Science Division, Boston, 1994).
150 Magruder, K. *et al.* Diagnostic performance of the PTSD checklist and the Vietnam Era Twin Registry PTSD scale. *Epidemiol Psychiatr Sci* **24**, 415-422, doi:10.1017/S2045796014000365 (2015).
151 Peters, E. S. *et al.* The Women and Their Children's Health (WaTCH) study: methods and design of a prospective cohort study in Louisiana to examine the health effects from the BP oil spill. *BMJ open* **7**, e014887 (2017).
152 Blevins, C. A., Weathers, F. W., Davis, M. T., Witte, T. K. & Domino, J. L. The Posttraumatic Stress Disorder Checklist for DSM-5 (PCL-5): Development and Initial Psychometric Evaluation. *J Trauma Stress* **28**, 489-498, doi:10.1002/jts.22059 (2015).
153 Xie, P. *et al.* Genome-wide association study identifies new susceptibility loci for posttraumatic stress disorder. *Biol Psychiatry* **74**, 656-663, doi:10.1016/j.biopsych.2013.04.013 (2013).
154 Duncan, L. E. *et al.* Largest GWAS of PTSD (N=20 070) yields genetic overlap with schizophrenia and sex differences in heritability. *Mol Psychiatry*, doi:10.1038/mp.2017.77 (2017).
155 Uhlen, M. *et al.* Proteomics. Tissue-based map of the human proteome. *Science* **347**, 1260419, doi:10.1126/science.1260419 (2015).
156 De, I. & Sadhukhan, S. Emerging roles of DHHC-mediated protein S-palmitoylation in physiological and pathophysiological context. *European journal of cell biology* **97**, 319-338 (2018).
157 Greenwood, T. A. *et al.* Further evidence for linkage of bipolar disorder to chromosomes 6 and 17 in a new independent pedigree series. *Bipolar disorders* **14**, 71-79 (2012).

158 Seirafi, M., Kozlov, G. & Gehring, K. Parkin structure and function. *The FEBS journal* **282**, 2076-2088 (2015).
159 Narendra, D., Tanaka, A., Suen, D.-F. & Youle, R. J. Parkin is recruited selectively to impaired mitochondria and promotes their autophagy. *The Journal of cell biology* **183**, 795-803 (2008).
160 Mouton-Liger, F., Jacoupy, M., Corvol, J.-C. & Corti, O. PINK1/Parkin-dependent mitochondrial surveillance: from pleiotropy to Parkinson's disease. *Frontiers in molecular neuroscience* **10**, 120 (2017).
161 Chopra, R. *et al.* PARK2 and proinflammatory/anti-inflammatory cytokine gene interactions contribute to the susceptibility to leprosy: a case–control study of North Indian population. *BMJ Open* **4**, e004239, doi:10.1136/bmjopen-2013-004239 (2014).
162 Lee, S. *et al.* Multiple-level validation identifies PARK2 in the development of lung cancer and chronic obstructive pulmonary disease. *Oncotarget* **7**, 44211 (2016).
163 La Cognata, V. *et al.* Increasing the coding potential of genomes through alternative splicing: the case of PARK2 gene. *Current genomics* **15**, 203-216 (2014).
164 Kalisch, R., Gerlicher, A. M. & Duvarci, S. A Dopaminergic Basis for Fear Extinction. *Trends in cognitive sciences* **23**, 274-277 (2019).
165 Lee, S.-A. *et al.* in *BMC bioinformatics.* S20 (BioMed Central).
166 Sevilla, L. M., Nachat, R., Groot, K. R. & Watt, F. M. Kazrin regulates keratinocyte cytoskeletal networks, intercellular junctions and differentiation. *J Cell Sci* **121**, 3561-3569 (2008).
167 Consortium, G. T. The Genotype-Tissue Expression (GTEx) project. *Nat Genet* **45**, 580-585, doi:10.1038/ng.2653 (2013).
168 Pietersen, C. Y. *et al.* Molecular profiles of parvalbumin-immunoreactive neurons in the superior temporal cortex in schizophrenia. *Journal of neurogenetics* **28**, 70-85 (2014).
169 Sun, X. R. *et al.* Amelioration of oxidative stress-induced phenotype loss of parvalbumin interneurons might contribute to the beneficial effects of environmental enrichment in a rat model of post-traumatic stress disorder. *Behavioural brain research* **312**, 84-92 (2016).
170 Glaab, E. & Schneider, R. Comparative pathway and network analysis of brain transcriptome changes during adult aging and in Parkinson's disease. *Neurobiol Dis* **74**, 1-13, doi:10.1016/j.nbd.2014.11.002 (2015).
171 Kärkkäinen, S., van der Linden, M. & Renkema, G. H. POSH2 is a RING finger E3 ligase with Rac1 binding activity through a partial CRIB domain. *FEBS letters* **584**, 3867-3872 (2010).
172 Kurochkina, N. & Guha, U. SH3 domains: modules of protein–protein interactions. *Biophysical reviews* **5**, 29-39 (2013).
173 George, A. J., Hoffiz, Y. C., Charles, A. J., Zhu, Y. & Mabb, A. M. A comprehensive atlas of E3 ubiquitin ligase mutations in neurological disorders. *Frontiers in genetics* **9**, 29 (2018).
174 Upadhyay, A. *et al.* E3 ubiquitin ligases neurobiological mechanisms: development to degeneration. *Frontiers in molecular neuroscience* **10**, 151 (2017).
175 Lee, J. H. *et al.* Genetic modifiers of age at onset in carriers of the G206A mutation in PSEN1 with familial Alzheimer disease among Caribbean Hispanics. *JAMA neurology* **72**, 1043-1051 (2015).
176 Nielsen, J. S. & McNagny, K. M. The role of podocalyxin in health and disease. *Journal of the American Society of Nephrology* **20**, 1669-1676 (2009).
177 Bauer, H.-C., Krizbai, I. A., Bauer, H. & Traweger, A. "You Shall Not Pass"—tight junctions of the blood brain barrier. *Frontiers in neuroscience* **8**, 392 (2014).
178 Chan, Y. E. *et al.* Post-traumatic Stress Disorder and Risk of Parkinson Disease: A Nationwide Longitudinal Study. *Am J Geriatr Psychiatry* **25**, 917-923, doi:10.1016/j.jagp.2017.03.012 (2017).
179 Smith, A. R., Mill, J., Smith, R. G. & Lunnon, K. Elucidating novel dysfunctional pathways in Alzheimer's disease by integrating loci identified in genetic and epigenetic studies. *Neuroepigenetics* **6**, 32-50 (2016).

180 Luykx, J., Giuliani, F., Giuliani, G. & Veldink, J. Coding and non-coding RNA dysregulation in bipolar disorder. *Preprint at https://www.biorxiv.org/content/10.1101/291385v1.abstract* (2018).
181 Naseer, M. I. *et al.* Genome wide analysis of novel copy number variations duplications/deletions of different epileptic patients in Saudi Arabia. *BMC genomics* **16**, S10 (2015).
182 Goes, F. S. *et al.* Genome-wide association study of schizophrenia in Ashkenazi Jews. *American Journal of Medical Genetics Part B: Neuropsychiatric Genetics* **168**, 649-659 (2015).
183 Lencer, R. *et al.* Genome-wide association studies of smooth pursuit and antisaccade eye movements in psychotic disorders: findings from the B-SNIP study. *Translational psychiatry* **7**, e1249 (2017).
184 Mokhtari, R. & Lachman, H. M. The major histocompatibility complex (MHC) in schizophrenia: a review. *Journal of clinical & cellular immunology* **7** (2016).
185 Wray, N. R. *et al.* Genome-wide association analyses identify 44 risk variants and refine the genetic architecture of major depression. *Nature genetics* **50**, 668 (2018).
186 Malavia, T. A. *et al.* Generating testable hypotheses for schizophrenia and rheumatoid arthritis pathogenesis by integrating epidemiological, genomic, and protein interaction data. *NPJ schizophrenia* **3**, 11 (2017).
187 Sekar, A. *et al.* Schizophrenia risk from complex variation of complement component 4. *Nature* **530**, 177-183, doi:10.1038/nature16549 (2016).
188 Watanabe, K., Taskesen, E., van Bochoven, A. & Posthuma, D. Functional mapping and annotation of genetic associations with FUMA. *Nature Communications* **8**, 1826, doi:10.1038/s41467-017-01261-5 (2017).
189 Griffiths-Jones, S., Grocock, R. J., Van Dongen, S., Bateman, A. & Enright, A. J. miRBase: microRNA sequences, targets and gene nomenclature. *Nucleic acids research* **34**, D140-D144 (2006).
190 Ng, S. Y., Johnson, R. & Stanton, L. W. Human long non-coding RNAs promote pluripotency and neuronal differentiation by association with chromatin modifiers and transcription factors. *The EMBO journal* **31**, 522-533, doi:10.1038/emboj.2011.459 (2012).
191 Austin, P. J. *et al.* Transcriptional profiling identifies the long noncoding RNA plasmacytoma variant translocation (PVT1) as a novel regulator of the asthmatic phenotype in human airway smooth muscle. *Journal of Allergy and Clinical Immunology* **139**, 780-789 (2017).
192 Braconi, C. *et al.* Expression and functional role of a transcribed noncoding RNA with an ultraconserved element in hepatocellular carcinoma. *Proceedings of the National Academy of Sciences* **108**, 786-791 (2011).
193 Nievergelt, C. M. *et al.* Genomic Approaches to Posttraumatic Stress Disorder: The Psychiatric Genomic Consortium Initiative. *Biol Psychiatry* **83**, 831-839, doi:10.1016/j.biopsych.2018.01.020 (2018).
194 Delgado-González, J. C. *et al.* Quantitative measurements in the human hippocampus and related areas: Correspondence between ex-vivo MRI and histological preparations. *PloS one* **10**, e0130314 (2015).
195 Qin, S. *et al.* Amygdala subregional structure and intrinsic functional connectivity predicts individual differences in anxiety during early childhood. *Biol Psychiatry* **75**, 892-900, doi:10.1016/j.biopsych.2013.10.006 (2014).
196 Roozendaal, B., McEwen, B. S. & Chattarji, S. Stress, memory and the amygdala. *Nat Rev Neurosci* **10**, 423-433, doi:10.1038/nrn2651 (2009).
197 Keifer, O. P., Jr., Hurt, R. C., Ressler, K. J. & Marvar, P. J. The Physiology of Fear: Reconceptualizing the Role of the Central Amygdala in Fear Learning. *Physiology (Bethesda)* **30**, 389-401, doi:10.1152/physiol.00058.2014 (2015).
198 Gelernter, J. *et al.* Genome-wide association study of post-traumatic stress disorder reexperiencing symptoms in >165,000 US veterans. *Nature Neuroscience* **22**, 1394-1401, doi:10.1038/s41593-019-0447-7 (2019).
