# Supplementary material for: International meta-analysis of PTSD genome-wide association studies identifies sex- and ancestry-specific genetic risk loci
Source: Nat Commun. 2019 Oct 8;10:4558. doi: 10.1038/s41467-019-12576-w (PMC6783435; doi:10.1038/s41467-019-12576-w)
Supplement: Supplementary file 5 — Reporting Summary [file 41467_2019_12576_MOESM5_ESM.pdf]

nature research

Corresponding author(s): Caroline Nievergelt

Last updated by author(s): 09/04/2019

Reporting Summary

Nature Research wishes to improve the reproducibility of the work that we publish. This form provides structure for consistency and transparency in reporting. For further information on Nature Research policies, see Authors & Referees and the Editorial Policy Checklist.

Statistics

For all statistical analyses, confirm that the following items are present in the figure legend, table legend, main text, or Methods section.

n/a	Confirmed	
☐	☒	The exact sample size (n) for each experimental group/condition, given as a discrete number and unit of measurement
☐	☒	A statement on whether measurements were taken from distinct samples or whether the same sample was measured repeatedly
☐	☒	The statistical test(s) used AND whether they are one- or two-sided<br>*Only common tests should be described solely by name; describe more complex techniques in the Methods section.*
☐	☒	A description of all covariates tested
☐	☒	A description of any assumptions or corrections, such as tests of normality and adjustment for multiple comparisons
☐	☒	A full description of the statistical parameters including central tendency (e.g. means) or other basic estimates (e.g. regression coefficient) AND variation (e.g. standard deviation) or associated estimates of uncertainty (e.g. confidence intervals)
☐	☒	For null hypothesis testing, the test statistic (e.g. F, t, r) with confidence intervals, effect sizes, degrees of freedom and P value noted<br>*Give P values as exact values whenever suitable.*
☐	☒	For Bayesian analysis, information on the choice of priors and Markov chain Monte Carlo settings
☐	☒	For hierarchical and complex designs, identification of the appropriate level for tests and full reporting of outcomes
☐	☒	Estimates of effect sizes (e.g. Cohen's d, Pearson's r), indicating how they were calculated

Our web collection on statistics for biologists contains articles on many of the points above.

Software and code

Policy information about availability of computer code

Data collection	Data collecting (phenotyping and genotyping) was performed previously by each cohort. Details on data collection in each cohort have been described previously, see the Supplementary Information for a full list of references. Broadly, phenotyping was not specifically dependent on specialized software, and genotyping was performed using standard genotype calling pipelines outside of the scope of the current study.
Data analysis	Ancestry was determined with SNPweights v2.1 (https://www.hsph.harvard.edu/alkes-price/software/). Quality control, imputation, and GWAS of case/control cohorts was performed using ricopili version Dec 2015b (https://github.com/Nealelab/ricopili), which includes wrappers around PLINK 1.9, Eigenstrat v5, LifeOver, SHAPEIT v2.r837, IMPUTE2 v2.2.2. For family and twin studies (VETSA, QIMR), analyses were performed using linear mixed models in GEMMA v0.96. The UKBB data (UKBB) were analyzed with BGenie v1.2. All software distributions were Linux versions. Meta-analyses were performed with metal version 2011-03-25 and METASOFT v.2.0.1. Multivariate conditional joint analysis (mtCojo) was performed using GCTA Version 1.91.6beta. LD score regression analyses were performed with ldsc v 1.0.0 (https://github.com/bulik/ldsc) and the LD Hub web tool (ldsc.broadinstitute.org). Gene-based analyses were performed with the FUMA web tool v 1.3.0 (http://fuma.ctglab.nl/), which uses MAGMA 1.07b. Polygenic risk estimates were calculated using PRSice 2.1.4 (https://choishingwan.github.io/PRSice/). Analyses related to local ancestry calling included scripts from Elizabeth Atkinson (https://github.com/eatkinson) using RFMix version 2 (https://github.com/slowkoni/rfmix). Plots were generated using R 3.1.0, LocusZoom (http://locuszoom.org/), ForestPMPlot (http://genetics.cs.ucla.edu/meta/). All of the above are publicly available, with relevant citations provided in the manuscript.

For manuscripts utilizing custom algorithms or software that are central to the research but not yet described in published literature, software must be made available to editors/reviewers. We strongly encourage code deposition in a community repository (e.g. GitHub). See the Nature Research guidelines for submitting code & software for further information.

Data

Policy information about availability of data

All manuscripts must include a data availability statement. This statement should provide the following information, where applicable:

- Accession codes, unique identifiers, or web links for publicly available datasets
- A list of figures that have associated raw data
- A description of any restrictions on data availability

Summary statistics from the genome-wide meta-analyses will be made available on the Psychiatric Genomics Consortium’s downloads page upon publication (http://www.med.unc.edu/pgc/results-and-downloads), including the source data for Figures 1 and 3. Individual-level data from the genotyped cohorts and cohort-level summary statistics will be made available to researchers following an approved analysis proposal through the PGC Post Traumatic Stress Disorder group with agreement of the cohort PIs; contact the corresponding authors for details. Publicly available genome-wide summary statistics used for testing genetic correlations seen in Figure 3 are accessible through LD Hub (http://ldsc.broadinstitute.org/). Summary statistics for the Million Veteran Project re-experiencing GWAS used for replication can be accssed thorugh dbGaP via accession number phs001672.v1.p.

Field-specific reporting

Please select the one below that is the best fit for your research. If you are not sure, read the appropriate sections before making your selection.

☒ Life sciences ☐ Behavioural & social sciences ☐ Ecological, evolutionary & environmental sciences

For a reference copy of the document with all sections, see nature.com/documents/nr-reporting-summary-flat.pdf

Life sciences study design

All studies must disclose on these points even when the disclosure is negative.

Sample size	Sample size was not predetermined, but instead reflects our best effort to aggregate all possible studies with genome-wide genotype data and robust phenotyping of post traumatic stress disorder. This open, international collaboration supported by the Psychiatric Genomics Consortium includes contributions from 60 studies and to our knowledge represents the largest genome-wide study of PTSD to date. Based on the available data, we have made efforts to maximize the use of the genotyped samples. This includes developing the infrastructure and appropriate statistical modeling to include both family-based and case/control cohorts in the same genome-wide analysis, and including trans-ancestral analysis of African, European, and Latino ancestry individuals. We have also performed power analysis for the current genome-wide study. For instance, we estimate that the full discovery meta-analysis has >80% power to detect variants associated with PTSD with true odds ratios >= 1.1 and minor allele frequency > 0.2. This power and sample size are consistent with successful GWAS of many other psychiatric traits
Data exclusions	Data exclusions were performed based on (a) failure of pre-determined data quality control criteria and (b) planned phenotype exclusions to insure valid case/control criteria. For quality control, individuals were excluded if they were observed to have low genotyping quality (detailed in methods). Ancestries other than African, European, or Latino were excluded due to insufficient sample size for a meaningful analysis in the currently available data. For phenotype-based exclusions, we omit individuals lacking phenotype information for PTSD. Cohorts with other exclusion criteria as part of their original study recruitment are detailed in the Supplementary Information. The metrics used as exclusion criteria were established prior to the analyses, but some thresholds used for exclusion (e.g. cutoffs from ancestry analysis to define ancestry strata) were evaluated during the QC process. All of the above exclusions were made in accordance with the planned study protocol, and are detailed in the manuscript.
Replication	For all genome-wide significant loci in the study, we attempted trans-ethnic replication as well as replication in the Million Veteran Program (MVP) cohort study of PTSD re-experiencing symptoms. As described in the manuscript, direct replication was not found. We note that lack of replication of across ancestry groups may be due to lack of power in the replication samples or differing linkage disequilibrium patterns. Lack of replication in the MVP cohort may reflect differences between the genetics of re-experiencing symptoms of PTSD and overall PTSD. For replication of a general PTSD signal in the data and to indicate generalizability of the overall results across cohorts, polygenic risk score analyses and genetic correlations were used. In all instances, polygenic risk scores derived from subsets of this study successfully predicted PTSD phenotypes in holdout data and significant genetic correlation was seen across different subsets of the data. In particular, we see that PRS significantly predict re-experiencing symptoms in the entirely independent MVP cohort.
Randomization	Randomization of experimental groups was not applicable to this study. The experimental conditions are determined by each individual's genetics, which are fixed at conception. Conceptually this reflects a randomization of the alleles inherited from each individual's parents (i.e. mendelian randomization), but it does not involve randomization of experimental conditions by the researchers in a classical sense. Our study assess the observed association between that natural randomization of genotype and the ascertained phenotype of PTSD.
Blinding	Blinding is not relevant to the current study. Samples were not allocated to different conditions by the researches, and the phenotype ascertainment process is fully separate from the genotyping process.

Reporting for specific materials, systems and methods

We require information from authors about some types of materials, experimental systems and methods used in many studies. Here, indicate whether each material, system or method listed is relevant to your study. If you are not sure if a list item applies to your research, read the appropriate section before selecting a response.

Materials & experimental systems

n/a	Involved in the study	
☒	☐	Antibodies
☐	☒	Eukaryotic cell lines
☒	☐	Palaeontology
☒	☐	Animals and other organisms
☐	☒	Human research participants
☒	☐	Clinical data

Methods

n/a	Involved in the study	
☒	☐	ChIP-seq
☒	☐	Flow cytometry
☒	☐	MRI-based neuroimaging

Eukaryotic cell lines

Policy information about cell lines

Cell line source(s)	Lymphoblastoid cell lines from 1000 Genomes AFR superpopulation, obtained from the Coriell Institute, NJ
Authentication	Cell lines were authenticated by the Coriell Institute using their standard procedures
Mycoplasma contamination	Cell lines tested negative for mycoplasma contamination
Commonly misidentified lines<br>(See ICLAC register)	No commonly misidentified cell lines were used in this research

Human research participants

Policy information about studies involving human research participants

Population characteristics	The current study encompasses 32,428 cases and 174,227 controls from 60 cohorts in the primary analysis. Details on each cohort are provided in the manuscript, with summary descriptive information in Supplemental Table 1 and full descriptions in the Supplementary Information. Briefly, included participants represent a mix of ascertainment schemes across cohorts, including both population-based collections and ascertained research cohorts. These include studies of genetically unrelated cases and controls, as well as studies of twins and families. Overall, the participants include roughly equal numbers of males and females, with ages fully distributed across the lifespan for adults. Participants are from North and South America, Africa, Europe, and Australia and are of European, Latino, or African ancestry (confirmed in genetic data),with African ancestry individuals predominantly reflecting African-American admixture. Genome-wide genotype data has been collected for all participants. Phenotyping criteria vary by cohort (full descriptions in the manuscript methods), but for most cohorts a standardized measure such as the Clinician Administered PTSD Scale (CAPS) or the PTSD Checklist (PCL) has been administered to ascertain current or lifetime PTSD status in accordance with DSM-IV diagnostic guidelines.
Recruitment	Participants were recruited separately for each cohort according to their respective study design. Descriptions of the design for each cohort can be found in the Supplementary Information, along with references to previous publications containing complete details. Overall, the cohorts represent a mix of population-based cohorts without targeted ascertainment (e.g.birth cohorts from a specified region), cohorts recruited for studies of PTSD (e.g. war veterans), or cohorts originally recruited for studies of other phenotypes where measures of PTSD were included in phenotyping (e.g. substance abuse). These recruitment strategies could yield biases in the results for a given cohort, but the mix of recruitment strategies used across the cohorts is unlikely to produces consistent biases across the current analysis. Instead, any different biases resulting from the variety of recruitment strategies and study designs would be more likely to manifest as heterogeneity or noise in results across the cohorts, potentially reducing power.
Ethics oversight	The study was approved by the University of California, San Diego Human Research Protection Program (IRB Project #160976)

Note that full information on the approval of the study protocol must also be provided in the manuscript.
