# Supplementary material for: International meta-analysis of PTSD genome-wide association studies identifies sex- and ancestry-specific genetic risk loci
Source: Nat Commun. 2019 Oct 8;10:4558. doi: 10.1038/s41467-019-12576-w (PMC6783435; doi:10.1038/s41467-019-12576-w)
Supplement: Supplementary file 6 — Description of Additional Supplementary Files [file 41467_2019_12576_MOESM6_ESM.pdf]

Title: Supplementary Data 1
Description: Demographics of PGC-PTSD Freeze 2 cohorts

Title: Supplementary Data 2
Description: Significant pathways and associated genes identified in MAGMA gene-set analyses

Title: Supplementary Data 3
Description: Genetic correlations of PTSD with several traits and disorders for data publicly available on LD Hub
